# Multifunctional NIR‐Triggered Nanozyme‐Based Microneedles for Synergistic Eradication of MRSA and Enhanced Wound Healing

**DOI:** 10.1002/advs.202510774

**Published:** 2025-07-23

**Authors:** Wei Qian, Ruixi Li, Xiyan Zheng, Yingliang Li, Haiwei Xiong, Ye Zhang, Dengliang Lei, Qingfeng Shi, Yufeng Xie, Yiting Zhou, Bailong Tao, Kuai Yu, Aiping Le, Boxuan Zhou

**Affiliations:** ^1^ Department of Breast Disease Center General Surgery Medical Center，Key Laboratory of Jiangxi Province for Transfusion Medicine the 1st Affiliated Hospital Jiangxi Medical College Nanchang University Nanchang 330006 P. R. China; ^2^ Department of Cardiology the 1st Affiliated Hospital, Jiangxi Medical College Nanchang University Nanchang 330006 P. R. China; ^3^ Department of Hepatobiliary and Pancreatic Surgery the Eighth Affiliated Hospital Sun Yat‐sen University Shenzhen 518033 P. R. China; ^4^ Laboratory Research Center the First Affiliated Hospital of Chongqing Medical University Chongqing 400016 P. R. China; ^5^ Department of Blood Transfusion the 1st Affiliated Hospital，Jiangxi Medical College, Nanchang University Nanchang 330006 P. R. China; ^6^ Postdoctoral Innovation Practice Base the 1st Affiliated Hospital Jiangxi Medical College Nanchang University Nanchang 330006 P. R. China

**Keywords:** angiogenesis, macrophage polarization, nitric oxide, photothermal effect, synergistic antibacterial property, wound healing

## Abstract

Antibacterial drug delivery for Methicillin‐resistant Staphylococcus aureus (MRSA)‐infected wounds faces challenges in reducing oxidative stress, reprogramming the inflammatory microenvironment, and promoting angiogenesis. Herein, a multifunctional near‐infrared (NIR) laser‐induced nanozymes (CTB) by integrating nitric oxide (NO)‐prodrug (BNN6) into a phenolic network of Cu2+‐tannic acid. The CTB nanozymes effectively eradicate MRSA through the synergistic effect of NIR‐triggered NO release and NIR‐induced local hyperthermia. Furthermore, the CTB nanozymes exhibit strong antioxidant, anti‐inflammatory, and angiogenic properties. To treat MRSA‐infected cutaneous wounds, novel microneedle patches (MN@CTB)are further developed by incorporating CTB nanozymes into hyaluronic acid methacrylate. The MN@CTB successfully eradicates bacterial infections, leveraging the synergistic effects of NO release and NIR‐induced local hyperthermia. MN@CTB regulates antioxidative and anti‐inflammatory effects by activating the Nrf‐2/HO‐1 signaling pathways and inhibiting the NF‐κB signaling pathway. Additionally, MN@CTB upregulates the expression of soluble guanylate cyclase (sGC), which further activates the protein kinase G (PKG) signaling pathway to stimulate angiogenesis. Proteomic analysis demonstrated the underlying mechanism by which the MN@CTB mainly reprogrammed the infected wound microenvironment by inhibiting the NF‐κB signaling pathway and activating the VEGF/TGF‐β signaling pathways. It is envisioned that this MN@CTB can work as a highly effective strategy for expediting the healing of MRSA‐infected cutaneous wounds.

## Graphical abstract

The novel microneedle patches (MN@CTB) are fabricated by integrating CTB nanozymes into HAMA and PVA solution based on a photo‐crosslinking reaction. The MN@CTB microneedle patches present an excellent photothermal effect and NIR‐stimulated release of NO, which is advantageous for the elimination of MRS. Meanwhile, this microneedle patche exhibits strong antioxidant and anti‐inflammatory capacities for remodeling the inflammatory microenvironment. Furthermore, the microneedle patches can significantly expedite wound healing and tissue regeneration by improving antibacterial ability, mitigating oxidative stress, alleviating inflammatory responses, enhancing neovascularization, and facilitating hair follicle regeneration and collagen deposition.

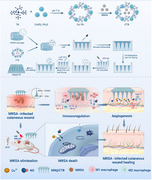

## 1 Introduction

Drug‐resistant bacterial infection, resulting from the widespread and indiscriminate use of antibiotics, poses a severe global threat to human health and life.^[^ ^1^ ^]^ Among those, methicillin‐resistant *Staphylococcus aureus* (MRSA) is the primary etiological agent of infected cutaneous wounds, which is associated with remarkable mortality and challenging recovery processes.^[^ ^2^ ^]^ Infected skin wounds involve a series of intricate stages: hemostasis, inflammation, cell proliferation, and tissue restriction, which often overlap.^[^ ^3^ ^]^ Persistent oxidative stress arises from an imbalance between reactive oxygen species (ROS)/reactive nitrogen species (RNS) and the antioxidant stress system.^[^ ^4^ ^]^ This imbalance can trigger inflammatory responses and further exacerbate the progression of infected cutaneous wounds^[^ ^5^ ^]^ Notably, bacterial deoxyribonucleic acid (DNA) and lipopolysaccharide (LPS) can activate macrophages and neutrophils, leading to the production of reactive oxygen species (ROS).^[^ ^6^ ^]^ Moreover, the overproduction of ROS and inflammatory mediators causes damage to the extracellular matrix (ECM) and hinders proper angiogenesis, thereby exacerbating the delay in wound healing.^[^ ^7^ ^]^ Hence, there is a pressing need to devise strategies that can effectively eradicate bacterial infection, scavenge ROS in the local microenvironment to shield cells from oxidative stress, alleviate inflammatory responses, and induce angiogenesis to treat infected cutaneous wounds.

Nanozymes, characterized by their excellent biocompatibility and stability, are currently being extensively utilized to mimic the functionalities of natural enzymes. They hold great promise across various domains, including neuroprotection, antibacterial treatment, anti‐inflammatory therapies, and cancer treatment.^[^ ^8^ ^]^ Evidence suggests that nanozymes can remove reactive oxygen species (ROS) and reactive nitrogen species (RNS) in inflammatory microenvironments by mimicking antioxidase capacities and promoting cells from oxidative stress damage.^[^ ^9^ ^]^ Leveraging these aforementioned properties, nanozymes have gained considerable attention in the treatment of infected cutaneous wounds. However, the application of single nanozymes is significantly limited due to the dynamic nature of the wound environment: 1) Due to the dynamic nature of infected cutaneous wounds, nanozymes often display burst‐release behavior, making them susceptible to rapid elimination by local immune reactions.^[^ ^10^ ^]^ 2) Most nanozymes present single or double functions, which lack the antibacterial capacity, antioxidative stress management, anti‐inflammatory effects, and angiogenetic requirements necessary for treating infected cutaneous wounds.^[^ ^11^ ^]^ Accordingly, there is a need to fabricate a versatile platform to exert the therapeutic effect of nanozymes on the infected cutaneous wounds, thereby adapting to handle the dynamic progression of infected wounds with multiple functions.

Gas therapy, which includes hydrogen (H_2_), hydrogen sulfide (H_2_S), sulfur dioxide (SO_2_), carbon monoxide (CO), and nitric oxide (NO), is a potential antibacterial approach that generally presents an enhanced antibacterial effect in association with light‐activatable strategies.^[^ ^12^ ^]^ Numerous studies suggested that NO gas is a crucial biological messenger molecule involved in vascular regulation, neurotransmission, inflammation, immune response, and antibacterial processes.^[^ ^13^ ^]^ Our previous studies revealed that the synergistic effect of NO‐medicated photothermal therapy (PTT) and NO gas therapy effectively eradicates MRSA biofilms.^[^ ^14^ ^]^ It was revealed that the combination of NO‐medicated immunotherapy and PTT presents promising applications in the management of MRSA‐infected cutaneous wounds. The bactericidal effectiveness of NO arises from inducing ROS generation, damaging protein and DNA molecules, which leads to changes in bacterial membrane permeability and leakage of cellular components.^[^ ^15^ ^]^ Importantly, it was revealed that NO exhibits an antibacterial capacity against bacterial invasion in mammals and will not be able to induce drug resistance. Moreover, NO gas offers unique advantages in alleviating inflammatory responses through its inhibition of inflammation‐relevant signaling pathways, such as MAPK (p38), JNK, and NF‐κB.^[^ ^16^ ^]^ Importantly, NO gas plays a vital role in promoting the migration, proliferation, and differentiation of endothelial cells. It stimulates the activation of stimulation of soluble guanylate cyclase (sGC), which leads to the up‐regulation of cyclic guanosine phosphate (cGMP) and protein kinase G (PKG), thereby facilitating angiogenesis.^[^ ^17^ ^]^ However, the imprecise therapeutic delivery, uncontrolled release behavior, and insufficient gas generation are extremely limiting the application in the treatment of infected wounds. Consequently, there is an urgent need to load NO‐prodrug into photo‐responsive nanomaterials, thereby achieving the “on‐demand” release of NO gas under NIR irradiation.

Apart from the direct antibacterial capacities, Cu^2+^ has been shown to enhance the expression of hypoxia‐inducible factor‐1α (HIF‐1α), consequently up‐regulating the expression of downstream angiogenesis‐related growth factors such as platelet endothelial cell adhesion molecule‐1 (CD31) and vascular endothelial growth factor (VEGF), thereby promoting angiogenesis.^[^ ^18^ ^]^ Consequently, Cu‐based nanozymes are anticipated to promote the angiogenesis of wounds. Tannic acid (TA), a natural polyphenol known for its potent antioxidant properties, functions as a superoxide dismutase (SOD) mimic, mitigating inflammatory responses and oxidative‐induced damage.^[^ ^19^ ^]^ In our previous studies, the introduction of TA/gallium ion (TA/Ga^3+^) on titanium (Ti) substrates, which possess excellent anti‐oxidative capacity and anti‐inflammatory effects for peri‐implants osseointegration.^[^ ^20^ ^]^ Additionally, we further functionalized Ti implants with TA/ferric ion (TA/Fe^3+^) coating, which possesses excellent antibacterial effects through multimodal and synergistic photothermal therapy.^[^ ^21^ ^]^ Our group successfully synthesized a nitric oxide (NO) donor molecule, N,N'‐di‐sec‐butyl‐N,N'‐dinitroso‐1,4‐phenylenediamine (BNN6), which presented a responsive NO release profile upon NIR irradiation.^[^ ^22^ ^]^ It was implied that this characteristic can be employed as a trigger to develop the responsive release of NO, thereby effectively impeding the progression of infection.

In this study, a microneedle patch system (MN@CTB) was first developed by loading a NO‐prodrug (BNN6) into Cu^2+^‐tannic acid (TA) nanozymes (CTB), followed by integrating CTB nanozymes into hyaluronic acid methacrylate (HAMA). This system had the following functions to combat MRSA‐infected cutaneous wounds: a) Synergistic antibacterial capacities: The photothermal effect of CTB nanozymes and NIR‐triggered release NO from MN@CTB microneedle patches system presented the synergistic antibacterial efficacy against commonly associated with infected cutaneous wounds, such as methicillin‐resistant *Staphylococcus aureus* (MRSA). b) Multiple ROS scavenging properties: CTB nanozymes are part of an antioxidative cascade metal‐phenolic nanozyme incorporating both enzymatic and nonenzymatic molecules, harnessing the superior nanozyme and natural antioxidant capacities of tannic acid (TA), enabling multiple reactive oxygen species (ROS) scavenging properties.^[^ ^23^ ^]^ TA, a natural polyphenol with antioxidant properties, can reduce the damage caused by oxidative stress via eliminating hydroxyl radicals (•OH), superoxide anions (O_2_^•−^), and hydrogen peroxide (H_2_O_2_).^[^ ^24^ ^]^ c) alleviation of inflammatory responses. Through the down‐regulation of NF‐κB and up‐regulation of Nrf‐2 and HO‐1, the MN@CTB microneedle patches system could activate macrophage polarization toward M2 phenotype, down‐regulation of pro‐inflammatory mediators as well as up‐regulation of the anti‐inflammatory mediators, thus contributing to the alleviation of inflammatory responses. d) Promoting human umbilical vein endothelial cell migration and angiogenesis: Through the transwell assay and proteomic analysis, the MN@CTB microneedle patches system could enhance cell migration and promote the expression of angiogenesis‐related genes and proteins by activating the VEGF and TGF‐β signaling pathways, further accelerating the regeneration of MRSA‐infected cutaneous wounds. Therefore, the MN@CTB microneedle patches system is anticipated to facilitate the effective healing of MRSA‐infected cutaneous wounds through a comprehensive approach. The photothermal MN@CTB microneedle patches offer promising potential for using photothermal nanozymes in the management of dynamic MRSA‐infected skin wounds, providing effective therapy without the risk of drug resistance.

## 2 Results and Discussion

### 2.1 Synthesis and Characterization of CTB Nanozymes

**Figure 1:**
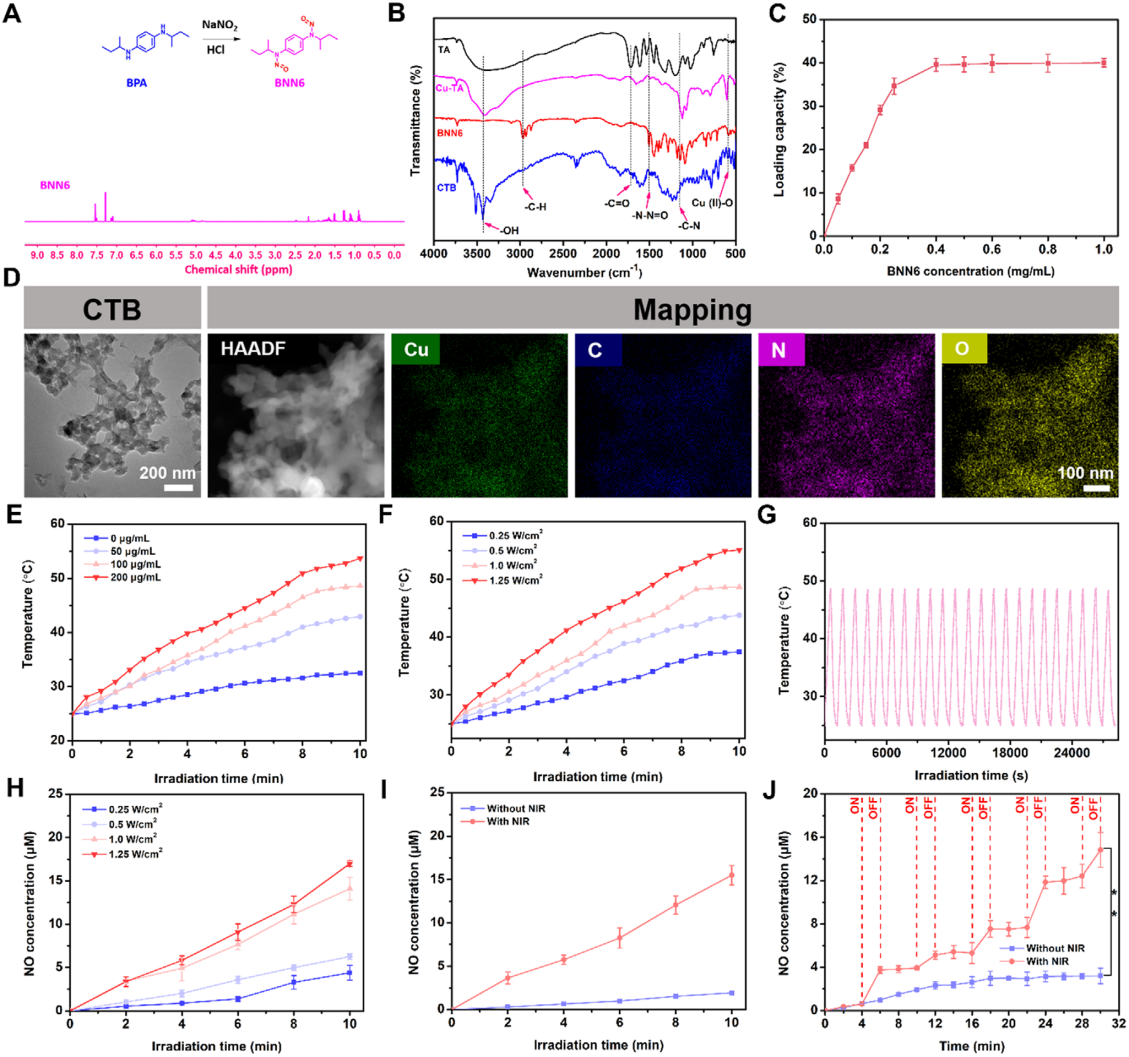
Characterization of CTB nanozymes. A) ^1^H NMR spectra of BNN6. B) FTIR spectra of TA, Cu‐TA, BNN6, and CTB nanozymes. C) Loading rates of various ratios of BNN6 on CTB nanozymes (*n* = 3). D) TEM image and mapping of CTB nanozymes. E) Temperature changes of various concentrations of CTB nanozymes (1.0 W cm^−2^). F) Temperature changes of CTB nanozymes with different power‐intensity NIR irradiation (0.25, 0.5, 1.0, and 1.25 W cm^−2^). G) Curves of the on/off test for MN@CTB nanozymes. H) NO‐releasing kinetics of CTB nanozymes with different power‐intensity NIR irradiation (0.25, 0.5, 1.0, and 1.25 W cm^−2^) (*n* = 3). I) The release assay of NO from CTB nanozymes with/without NIR irradiation (*n* = 3). J) On/off effect of the NO‐releasing profiles (*n* = 3).

The Cu‐TA nanozyme was synthesized by the oxidative coupling assembly of copper ions (Cu^2+^) and tannic acid (TA), which can coordinate with Cu^2+^ to establish a metal‐phenolic network due to its strong chelating properties.^[^ ^25^ ^]^ Next, the Cu‐TA nanozyme was utilized to serve as a drug delivery system for NO donor molecules by loading BNN6. Following a previously established procedure, the NO pro‐drug BNN6 was synthesized by replacing the hydrogen atoms of the secondary amino groups with nitroso groups on p‐phenylenediamine molecules.^[^ ^26^ ^]^ The results from nuclear magnetic resonance (^1^HNMR) analysis demonstrated that the characteristic peaks of hydrogen atoms were found in 7.2–7.6 ppm on the BNN6 benzene ring (**Figure** **1**A). This finding confirms the successful synthesis of the BNN6 molecule, as also noted in our earlier study.^[^ ^22^ ^]^

Subsequently, the CTB nanozymes were analyzed using several techniques, including Fourier Transform Infrared Spectroscopy (FTIR), X‐ray Diffraction (XRD), and Transmission Electron Microscopy (TEM) imaging. The FTIR spectrum of Cu‐TA displayed the characteristic stretching vibration peaks at 3405, 1714, and 601 cm^−1^, which corresponded to the υ‐OH, υ‐C = O, and infrared activation vibration mode of Cu(II)−O, respectively, indicating the Cu‐TA nanozyme was successfully synthesized.^[^ ^27^ ^]^ Absorption peaks of CTB nanozymes at 1136, 1506, and 2950 cm^−1^ attributed to the ─C─N bond, ─N─N ═ O bond, and ─C─H bond of BNN6, respectively, reveal its successful loading onto the Cu‐TA nanozyme (Figure 1B). The loading efficiency of BNN6 within the Cu‐TA nanozyme was evaluated using UV–vis spectroscopy. A standard curve of BNN6 was determined by measuring the absorbance of various concentrations of BNN6 (Figure S1, Supporting Information). As illustrated in Figure 1C, BNN6 loading efficiency attained saturation at concentrations of 0.4 mg and above, with the optimal loading efficiency of 39.6%. Therefore, a 0.4:1 ratio of BNN6 to Cu‐TA nanozyme was adopted for the synthesis of CTB nanozymes. The TEM image of CTB nanozymes, depicted in Figure 1D, presented mainly irregular and spherical structures. TEM elemental mappings revealed that the N element was uniformly distributed in CTB nanozymes (Figure 1D). Subsequently, the zeta potential of Cu‐TA shifted from −11.64 to −15.41 mV after the loading of BNN6 (Figure S2, Supporting Information). This phenomenon resulted from the electronegativity of BNN6.^[^ ^28^ ^]^ Differential light scattering (DLS) analysis revealed that the average hydrodynamic sizes of Cu‐TA and CTB nanozymes were ≈163 and 176 nm (Figure S3, Supporting Information), respectively. This size augmentation is likely attributed to the incorporation of BNN6 into the Cu‐TA nanozyme through *π‐π* stacking interactions.^[^ ^28^ ^]^

### 2.2 Photothermal Conversion Performance and NO Release of CTB Nanozymes

The in vitro photothermal conversion ability of CTB nanozymes was evaluated using a digital NIR photothermal imaging system. First, aqueous solutions of CTB nanozymes with various concentrations were subjected to NIR irradiation, and the temperature changes were recorded individually. The temperature change of 50, 100, and 200 µg mL^−1^ of CTB nanozymes reached 42.9, 48.6, and 53.7 °C, respectively (Figure 1E; Figure S4, Supporting Information). In comparison, the temperature change of PBS (0 µg mL^−1^ of CTB nanozymes) barely rose to 32.5 °C. In Figure 1F, the power density‐dependent temperature change of CTB nanozymes solution was observed, and the temperature reached 37.5, 43.8, 48.7, and 55.1 °C within 10 min when the power intensity ranged from 0.25 to 1.25 W cm^−2^, which implied that CTB nanozymes possess good photothermal performance under NIR light irradiation.^[^ ^29^ ^]^ Next, the photothermal stability of CTB nanozymes was estimated by evaluation of the temperature variation in five healing and cooling cycles under irradiation (100 µg mL^−1^, 1 W cm^−2^). It was implied that no remarkable temperature change was found during the adjacent peaks in each cycle, suggesting the high photothermal stability of the CTB nanozymes (Figure 1G). According to the heating‐cooling curve (Figure S5, Supporting Information) and corresponding thermal time constant (τ_s_) (Figure S6, Supporting Information), the photothermal conversion efficiency (η) of 35.8% for CTB nanozymes under 808 nm. These results revealed that the CTB nanozymes can efficiently convert laser radiation into thermal energy, which could be a competitive candidate for PTT.

Moreover, the thermoresponsive properties of the BNN6 donor molecule were employed to utilize the NIR‐irradiated NO controllable release system.^[^ ^28^ ^]^ Cu‐TA can effectively convert light energy into local hyperthermia, thereby activating BNN6 to release NO. The NO release profile of CTB nanozymes (100 µg mL^−1^) was assessed under NIR irradiation at different laser power densities, and the NO production was quantified using the Griess reagent. As shown in Figure 1H, the NO generation exhibited power density‐dependent characteristics for NIR‐triggered CTB nanozymes. The concentrations of NO released from CTB nanozymes were 4.39, 6.26, 14.10, and 16.99 µm after 10 min of irradiation at laser power densities of 0.25–1.25 W cm^−2^, respectively. In contrast, only a small amount of NO was released, with the concentration of 1.92 µm (Figure 1I), indicating that the BNN6 is stable in CTB nanozymes without NIR irradiation. Also, the production of NO exhibited concentration‐dependent capacities for NIR‐triggered CTB nanozymes (Figure S7, Supporting Information). In Figure 1J, the NO was rapidly released upon NIR laser irradiation, and the release rate slowed down once the irradiation was turned off, which implied “on‐demand” release properties of NO from CTB nanozymes under NIR irradiation.

### 2.3 Antibacterial Properties of CTB Nanozymes

**Figure 2:**
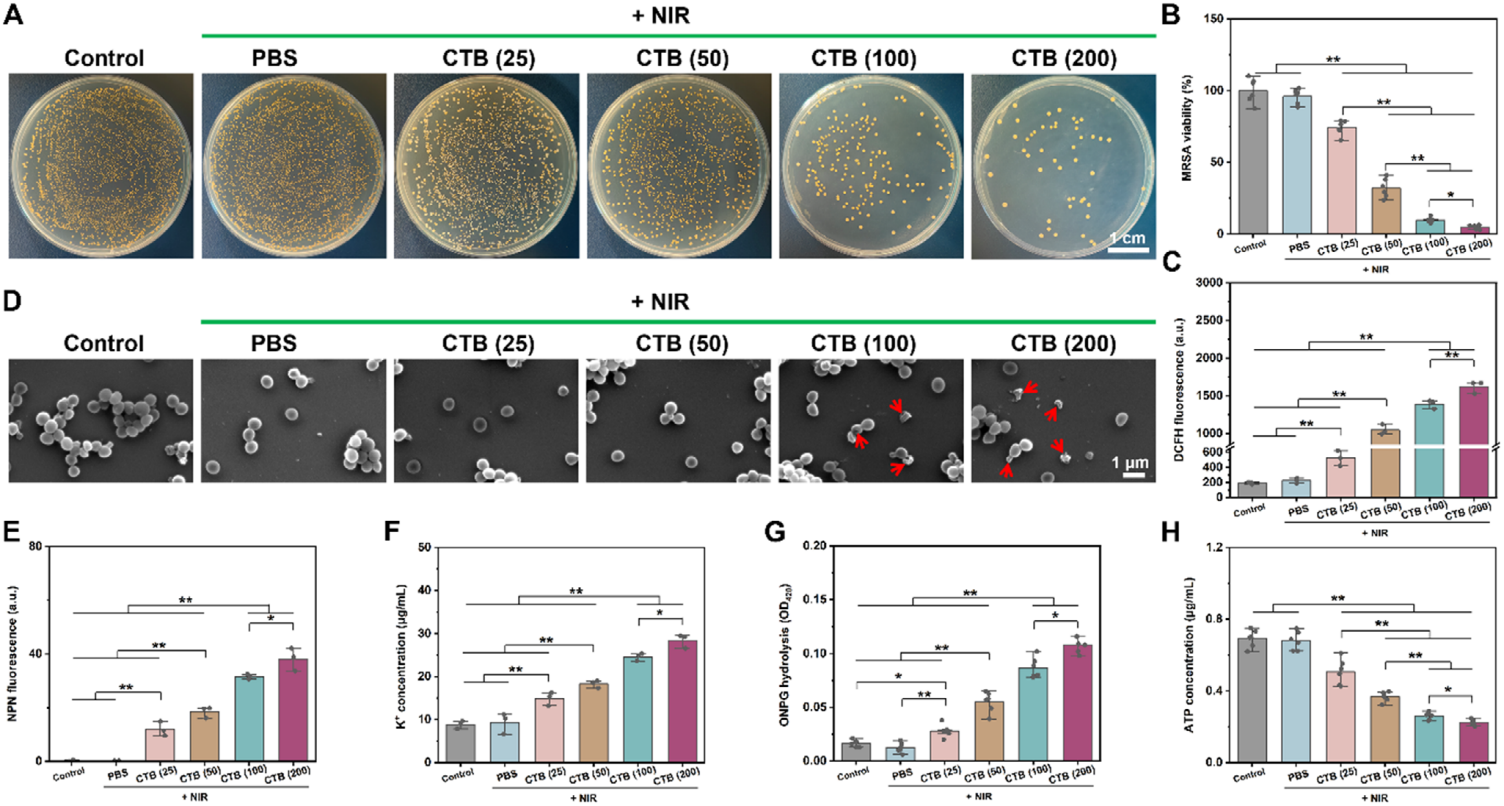
The antibacterial capacity of various concentrations of CTB nanozymes. A) Representative images of bacterial colonies formed by MRSA after different treatments. B) The corresponding statistical analysis of the MRSA viability (*n* = 6). C) ROS intensity of MRSA after incubation with various samples (*n* = 3). D) SEM images of MRSA after different treatments, the red arrows indicate the damaged morphology of MRSA. E) NPN fluorescence intensity (*n* = 3), F) K^+^ concentration (*n* = 3), G) ONPG hydrolysis (*n* = 6), and H) ATP concentration of MRSA after incubation with various samples (*n* = 6), ^*^ *p* < 0.05, ^**^ *p* < 0.01.

Inspired by the remarkable photothermal conversion of CTB nanozymes, we assessed their corresponding antimicrobial effects in vitro. No remarkable difference was observed between bacteria treated with PBS upon NIR irradiation and untreated bacteria (control), suggesting that the bare NIR irradiation presented almost no antibacterial ability. Due to their good photothermal effects, CTB nanozymes can convert the laser energy to local hyperthermia to kill bacteria effectively. Consequently, after culturing MRSA in the presence of CTB nanozymes, antibacterial ratios were positively correlated with CTB nanozymes concentration under NIR irradiation (**Figure** **2**A). Compared with control and PBS + NIR groups, the MRSA viabilities were remarkably inhibited after adding CTB nanozymes, and even when CTB nanozymes concentration reached 200 µg mL^−1^, MRSA was essentially killed (Figure 2B).

The exceptional antibacterial efficacy of CTB nanozymes under NIR irradiation likely arises from several mechanisms. First, the generation of reactive oxygen species (ROS) alongside the release of nitric oxide (NO) and copper ions (Cu^2+^) is pivotal in the antibacterial action of CTB nanozymes under NIR irradiation. These ROS can damage the integrity of the bacterial membrane, causing leakage of intracellular contents such as potassium ions (K^+^), proteins, and nucleic acids. Additionally, this process enhances ONPG hydrolysis and reduces ATP intensity, thereby altering the permeability of bacterial membranes and ultimately resulting in bacterial death. To verify this hypothesis regarding the antibacterial mechanism, we further studied the ROS intensity and Cu^2+^ concentration in the presence of CTB nanozymes under NIR irradiation. ROS accumulation was investigated by using a fluorescent probe dye (DCF‐DA). The results revealed that the content of ROS gradually increased, accompanied by the increased concentration of CTB nanozymes, whereas there were almost no changes in the control and PBS + NIR groups (Figure 2C). Scanning electron microscopy (SEM) was used to examine the structural characteristics of MRSA following various treatments. MRSA exhibited its typical smooth shape and intact cytoplasmic membranes without any change in the control and PBS + NIR groups (Figure 2D). In contrast, partial collapses of the cytoplasmic membrane were found in the CTB (25) and CTB (50) + NIR groups. Especially, the cell membranes of MRSA treated with 100 and 200 µg mL^−1^ CTB nanozymes with NIR irradiation were shrank and ruptured (indicated by red arrows), suggesting the strong antibacterial effect resulted from the photothermal therapy of the CTB (200) group.

Apart from SEM observation, a phenylnapthylamine (NPN) assay was also performed to evaluate the integrity of the cell membrane. As a lipophilic fluorescent probe, NPN can permeate into the intact cell membrane, once the bacterial membrane permeability is changed. The higher intensity of the fluorescence signal by NPN was found in the CTB (200) group under NIR irradiation, indicating greater destruction of bacterial integrity (Figure 2E). In Figure 2F, the extracellular released K^+^ concentration also corresponded to the trend of NPN fluorescence. Moreover, the integrity of the cell membrane was investigated by detecting the leakage of intra‐bacterial protein and nucleic acid. The levels of cellular component leakage in the CTB (200) group were significantly higher compared to the other groups (Figures S8 and S9, Supporting Information).

Subsequently, the membrane damage of MRSA was further investigated by measuring the ONPG hydrolysis levels and intracellular ATP, respectively. It was demonstrated that OPNG can penetrate into the intact bacteria and react with intracellular β‐D galactosidase, which is tailored for the assessment of membrane damage. In comparison to the control group, the level of ONPG hydrolysis in the PBS + NIR group was negligible, indicating that the NIR irradiation alone was not highly bactericidal. In contrast, higher levels of ONPG hydrolysis were observed in the CTB (25), CTB (50), CTB (100), and CTB (200) + NIR groups. More importantly, upon NIR irradiation, the CTB (200) group presented the highest level of ONPG hydrolysis (Figure 2G), implying more destruction of the membrane. In terms of intracellular ATP levels, a notable decrease was observed in the CTB (100) and CTB (200) groups upon NIR irradiation (Figure 2H), implying that the energy supply chain of MRSA was severely disrupted in the presence of these concentrations of CTB nanozymes. Besides, gram‐negative *E. coli* and gram‐positive *S. aureus* were chosen as the representative non‐resistant bacteria for investigating the antibacterial effect of various groups. As shown in Figures S10 and S11 (Supporting Information), similar trends of *E. coli* and *S. aureus* were found, compared with the antibacterial effect of MRSA. These in vitro results strongly confirmed that the CTB nanozymes possessed remarkable antibacterial ability against *E. coli*, *S. aureus*, and MRSA under NIR light irradiation.

Additionally, the CT (200), CTB (200) + NAC, and CTB (200) + EDTA groups were utilized to investigate the antibacterial effect of NO, ROS, Cu^2^⁺, and hyperthermia. As shown in Figure S12 (Supporting Information), the MRSA viability of CT (200) and CTB (200) was significantly lower than that of the control group. However, no statistical difference was found between the CT (200) and CTB (200) groups. After the addition of ROS scavenger (N‐acetylcysteine, NAC), the relative MRSA viability of CTB (200) + NAC group reached 82.0%, which was significantly higher than that of the CTB (200) group. Similarly, with the addition of Cu^2^⁺ chelator (ethylene diamine tetraacetic acid, EDTA), the same phenomenon was found, indicating that the generation of ROS and Cu^2+^ played a vital role in the improvement of antibacterial viability in CTB nanozymes. Upon NIR irradiation, the MRSA viabilities in the CT (200) and CTB (200) groups were 36.5% and 5.6%, respectively, revealing that the release of NO could enhance the antibacterial viability of CTB nanozymes. Furthermore, MRSA viability was 66.5% in the CTB group, it significantly decreased to 5.6% in the CTB (200) + NIR group, implying that the generation of hyperthermia could effectively inhibit the MRSA viability.

**Scheme 1:**
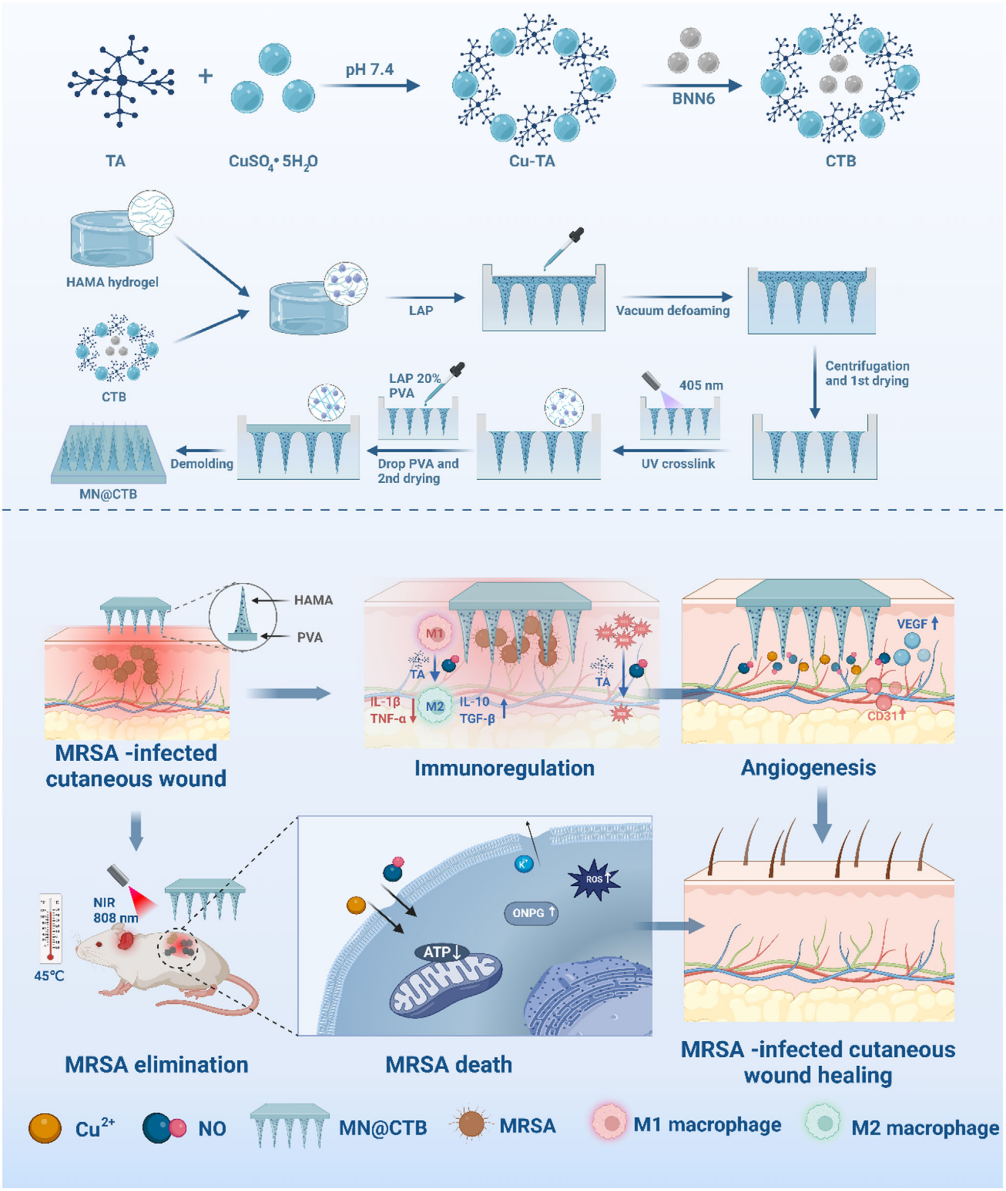
Schematic illustrations of the fabrication of MN@CTB microneedle patches and the application in the synergistic treatment of MRSA‐infected cutaneous wounds.

Based on the aforementioned results, the antibacterial mechanism of CTB nanozymes is derived from local hyperthermia, ROS accumulation, and release of NO and Cu^2+^, as illustrated in **Scheme** **1**. First, the local hyperthermia induced by the photothermal effect of CTB nanozymes sensitized the bacterial membrane to the external environment. Second, the release of NO and Cu^2+^ can induce ROS generation, and disrupt the integrity of the cell membrane, leading to the “out‐diffusion” of K^+^ and the leakage of cellular components such as intracellular proteins and nucleic acids. Third, the ONPG hydrolysis was remarkably enhanced, and the ATP level was markedly decreased once the bacterial membrane was damaged, ultimately leading to cell death. Consequently, these synergistic effects elucidated from multiple antibacterial pathways endowed CTB nanozymes with excellent antibacterial activity upon NIR irradiation.

### 2.4 Antioxidant and Anti‐Inflammatory Capacities of CTB Nanozymes

**Figure 3:**
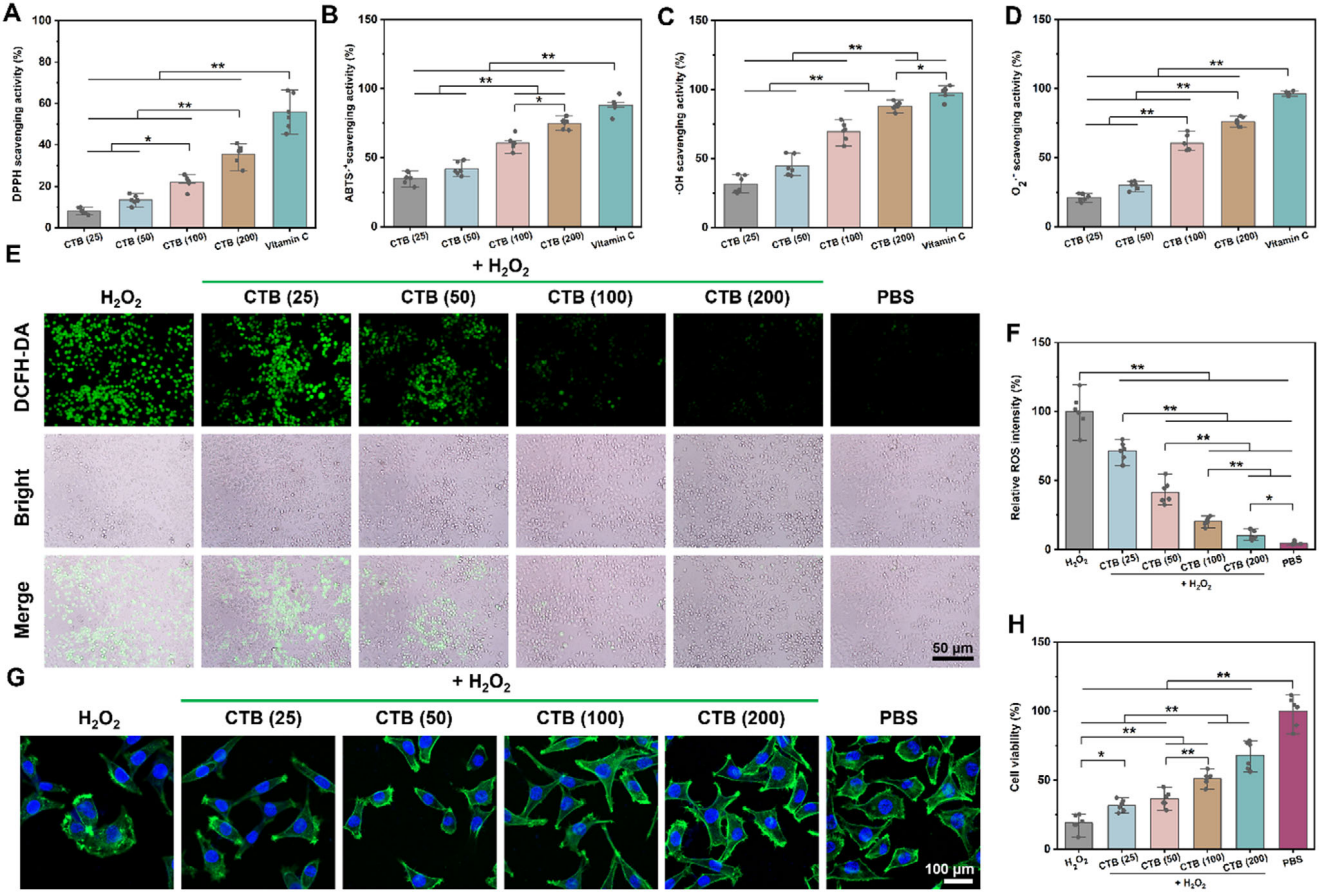
Antioxidation ability and biocompatibility of various concentrations of CTB nanozymes in vitro. A) DPPH scavenging activity, B) ABTS^•+^ scavenging efficiency, C) •OH scavenging activity, and D) O_2_^•−^ scavenging activity for each group. E) ROS fluorescence images after different treatments. F) Statistical analysis of the ROS fluorescence intensity. G). Cytoskeleton staining images of L929 cells after incubation with H_2_O_2_ (0.1 mm) and treated with various concentrations of CTB nanozymes. H) Relative cell viability of L929 cells after 24 h of coincubation with various concentrations of CTB nanozymes and 0.1 mm H_2_O_2_ (*n* = 6), ^*^ *p* < 0.05, ^**^ *p* < 0.01.

Excessive free radicals were acknowledged to be the dominant cause of harmful ROS and reactive nitrogen species (RNS) generation, which resulted in continuous oxidative stress, delaying the wound healing process. Our previous studies revealed that the abundant phenolic hydroxyl groups of TA could scavenge a series of free radicals via electron transfer or hydrogen atom donation.^[^ ^30^ ^]^ Therefore, the antioxidant abilities of CTB nanozymes were analyzed by monitoring the DPPH and ABTS^+^ free radical scavenging activities of CTB nanozymes. In **Figure** **3**A, the DPPH scavenging activity (%) results revealed that CTB nanozymes presented excellent DPPH clearance in a dose‐dependent manner. Besides, the ABTS^•+^ scavenging ability (%) exhibited a similar trend (Figure 3B).

Furthermore, the ROS scavenging abilities of the CTB nanozymes were investigated using two representative ROS (O_2_^•−^ and •OH). The 3,3,5,5‐tetramethylbenzidine (TMB) probe was employed to assess the scavenging efficiency of •OH by CTB nanozymes. As the concentration of CTB nanozymes increased, the •OH scavenging activity (%) gradually improved. The •OH scavenging activity reached 88.0% when the concentration of CTB nanozymes exceeded 200 µg mL^−1^ (Figure 3C). Compared with the CTB (25), CTB (50), and CTB (100) groups, the CTB (200) group exhibited higher O_2_^•‐^ scavenging activity and removed 79.2% of O_2_^•−^ (Figure 3D). Consequently, the CTB nanozymes possessed significant free radical scavenging activity, elaborating their antioxidative capacity during the early stage of the infected wound healing, thus alleviating the persistent oxidative stress in infected chronic wounds.

The cellular ROS scavenging capacity of CTB nanozymes was investigated using L929 cells under an oxidative stress microenvironment created by H_2_O_2_ in our previous reports.^[^ ^30^ ^]^ Compared with the control and PBS groups, the green fluorescence intensity was lower in L929 cells co‐cultured with various concentrations of CTB nanozymes. Importantly, the relative ROS intensity result revealed that CTB nanozymes effectively relieved intracellular oxidative stress in a dose‐dependent manner (Figure 3E,F). Moreover, the protective effect of CTB nanozymes on L929 cells was investigated by cytoskeleton staining and CCK‐8 assays under oxidative stress conditions. The H_2_O_2_ group exhibited dramatic negative effects on cytoskeletal damage to L929 cells (Figure 3G). In comparison, the L929 cells exhibited spindle‐shaped morphology after treatment with CTB nanozymes. Especially, the morphology of L929 cells treated with both H_2_O_2_ and 200 µg mL^−1^ CTB nanozymes closely resembled that of the control group. For CCK‐8 assay, the H_2_O_2_ group exhibited significant negative effects on cell viability (19.1%), but the addition of CTB nanozymes effectively relieved the H_2_O_2_‐induced damage on L929 cells. Importantly, the relative cell viability in the CTB (25), CTB (50), CTB (100), and CTB (200) reached 31.6%, 36.4%, 51.2%, and 68.0%, respectively (Figure 3H). This phenomenon might be attributed to the H_2_O_2_ was not completely removed by CTB nanozymes (200 µg mL^−1^), and residual H_2_O_2_ could consequently induce cytotoxicity in L929 cells through mechanisms including lipid peroxidation and DNA damage.^[^ ^30^ ^]^ To further evaluate oxidative stress responses, the observation period was extended to 48 h. Notably, L929 cells treated with 0.1 mm H_2_O_2_ alone exhibited a significant reduction in viability to 35.5%, whereas the survival rate of L929 cells rose to 93.8% in the CTB (200) group (Figure S13, Supporting Information). Taken together, these results revealed that CTB nanozymes possess good antioxidant capacities, effectively scavenging free radicals, mitigating oxidative stress, and thereby alleviating damage to cell proliferation induced by oxidative stress.

### 2.5 Angiogenesis Effects of CTB Nanozymes

**Figure 4:**
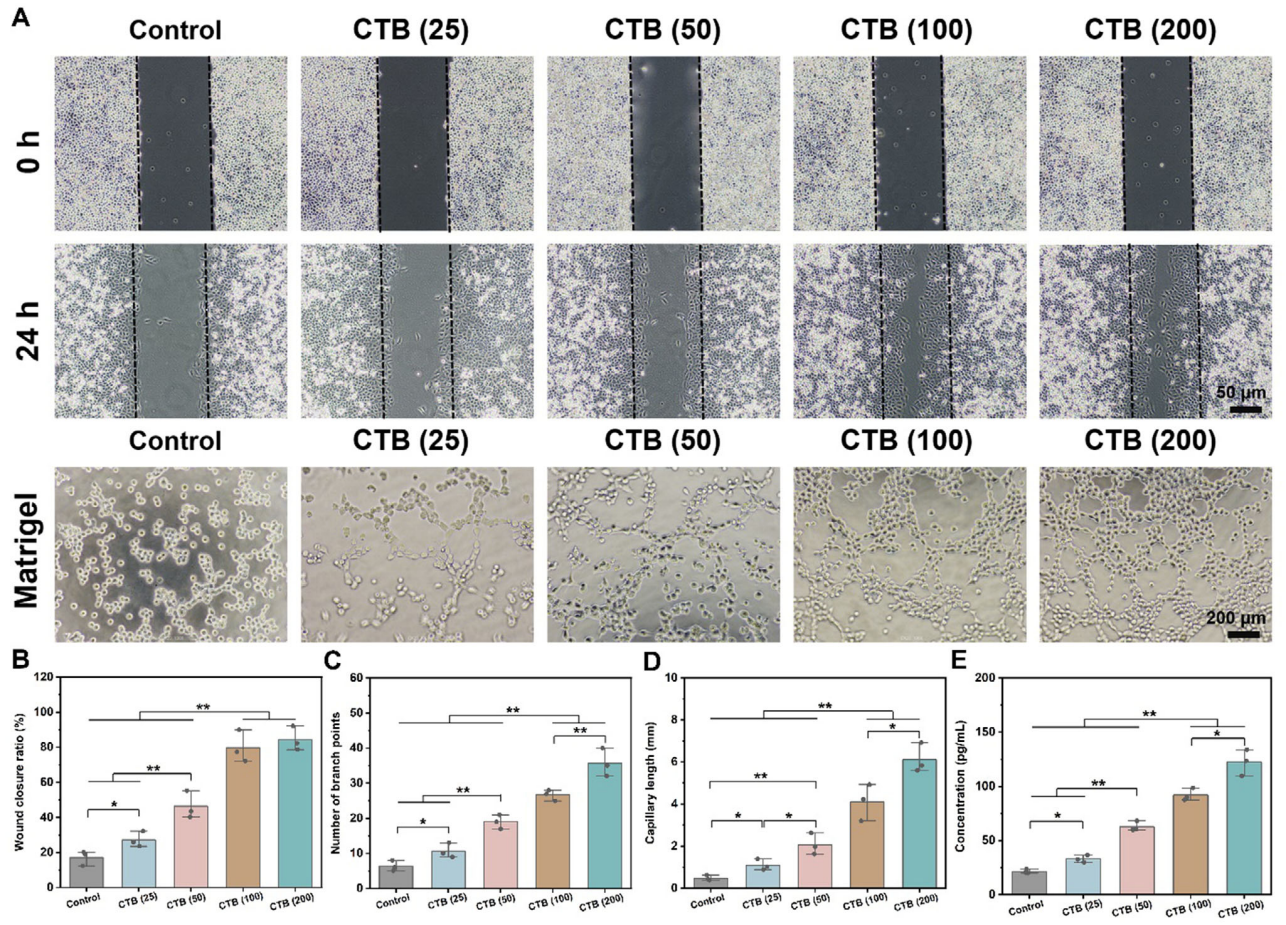
Angiogenic properties of various concentrations of CTB nanozymes. A) Representative images of migration and tube formation assay of HUVECs in each group. B) Statistical analysis of the wound closure ratio (%) (*n* = 3). C) Statistical analysis of the number of branch points (*n* = 3) and D) capillary length (*n* = 3), respectively. E) Expression level of VEGF in each group (*n* = 3), *p < 0.05, **p < 0.01.

Previous studies implied that the capacities to promote cell migration and angiogenesis are vital for skin regeneration.^[^ ^31^ ^]^ The wound width in each group was comparable at 0 h, in comparison, HUVECs gradually migrated to the center of wound sites at 24 h (**Figure** **4**A). In Figure 4B, the quantitative analysis results demonstrated that the wound closure ratio (%) of CTB (25), CTB (50), CTB (100), and CTB (200) nanozymes was 27.2%, 46.4%, 79.8%, and 84.3%, respectively, compared to 17.1% in the control group. This phenomenon could be attributed to the fact that the release of Cu^2+^ from the CTB nanozymes activated cell migration. Previous revealed that the Cu^2+^ exerted a positive effect on improving angiogenesis via activating the expression of angiogenesis‐related growth factors, such as platelet endothelial cell adhesion molecule‐1 (CD31) and vascular endothelial factor (VEGF), in HUVECs.^[^ ^7^ ^]^ Subsequently, we have investigated the effect of CTB nanozymes on angiogenesis via a tube formation assay. Compared with the control group, CTB nanozymes remarkably induced in vitro angiogenesis, and more branch points and longer capillary lengths were found in CTB nanozymes‐treated HUVECs, particularly CTB (200) group (Figure 4A). Quantitative analysis confirms that the tube formation in the CTB nanozymes groups has a larger number of branch points (Figure 4C) and longer capillary length (Figure 4D). In addition, the angiogenic cytokine VEGF in the CTB (200) group increased to 122.4 pg mL^−1^ from 21.3 pg mL^−1^ in the control group (Figure 4E). These results imply that CTB nanozymes not only promoted cell migration but also improved vessel formation of HUVECs in the later stage.

### 2.6 Preparation and Characterization of MN@CTB Microneedle Patches

**Figure 5:**
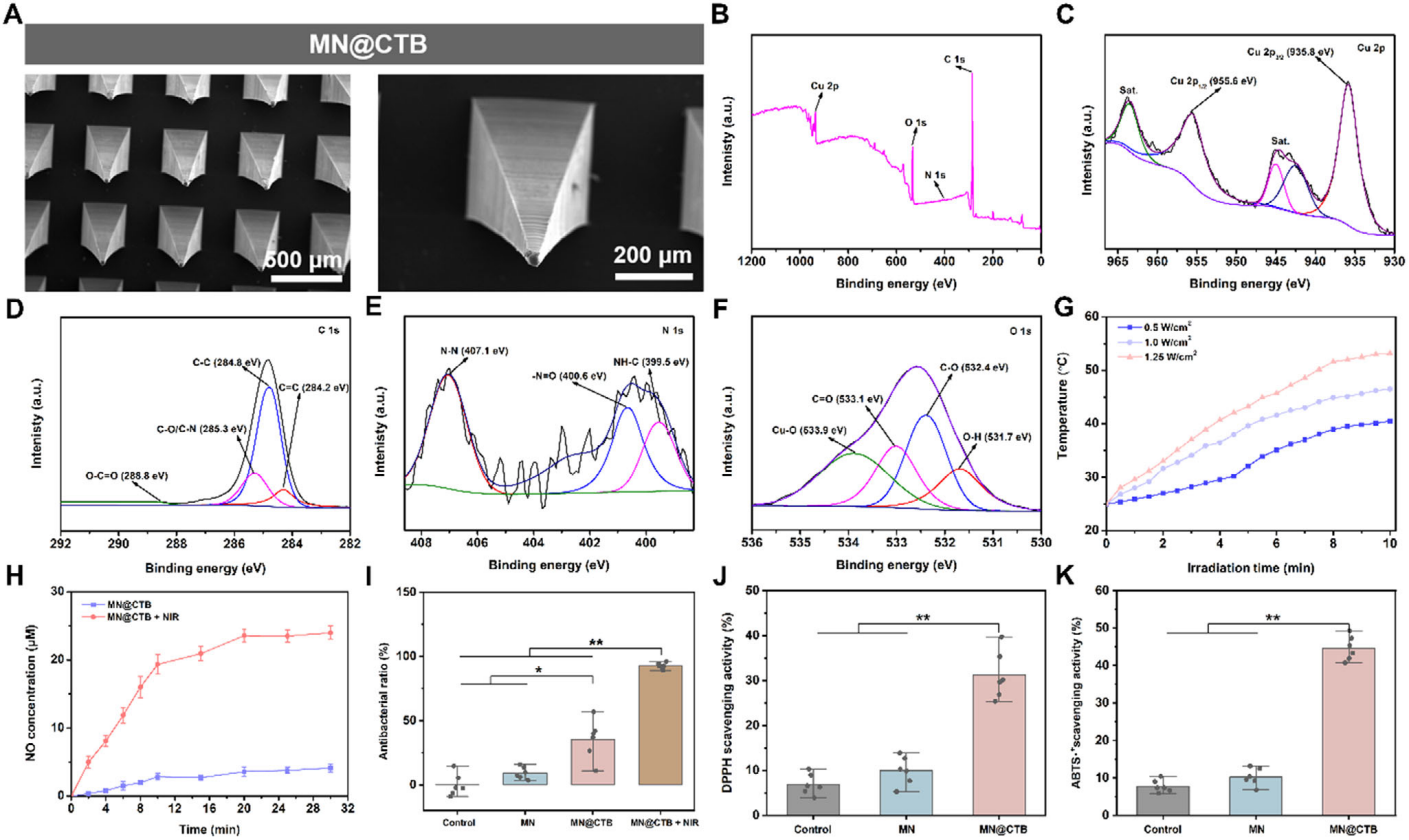
Characterization of MN@CTB microneedle patches. A) SEM images of MN@CTB microneedle patches. B) XPS spectra of MN@CTB microneedle patches. C) XPS survey of Cu 2p spectra. D) C 1 s, E) N 1s, and F) O 1s fine spectrums. G) Temperature changes of MN@CTB microneedle patches after different density‐power of NIR irradiation (808 nm, 0.5, 1.0, and 1.25 W cm^−2^) for 10 min. H) Cumulative release of NO from MN@CTB microneedle patches with/without NIR irradiation (1.0 W cm^−2^) for 30 min (*n* = 3). I) Antibacterial effect (*n* = 6), (J) DPPH scavenging activity (%) (*n* = 6), and ABTS^•+^ scavenging efficiency of MN@CTB microneedle patches (*n* = 6), ^*^ *p* < 0.05, ^**^ *p* < 0.01.

Subsequently, CTB nanozymes mixed in HAMA precursor were poured into polydimethylsiloxane (PDMS) molds with highly ordered microneedle (MN) patterns. After vacuum de‐bubbling and drying, the needle tip structures were fabricated by the photo‐crosslinking reaction. Thereafter, a polyvinyl alcohol (PVA) solution was added for casting the substrates and dried overnight to make MN@CTB microneedle patches.^[^ ^32^ ^]^ The MN@CTB microneedle patches had a center‐to‐center distance of 550 µm between adjacent tips, while the microneedle density was 20 × 20. The MN@CTB microneedle patches have a height of ≈700 µm and a base radius of 300 µm (**Figure** **5**A; Figure S14A, Supporting Information). The storage stability of MN@CTB microneedle patches was investigated by retrieving them from the backs of mice with predetermined time intervals. As shown in Figure S14B (Supporting Information), the needle tips of MN@CTB microneedle patches were nearly dissolved within 10 min. Furthermore, mechanical and adherent strength tests suggested that the mechanical penetration force and adhesive capacities in the MN@CTB microneedle were enhanced after loading of CTB nanozymes (Figure S14C,D, Supporting Information), compared with the MN microneedle. This phenomenon was attributed to the interactions between the polyphenol groups of TA, HAMA, and PVA, which bolstered the mechanical strength. EDS‐mapping of the MN@CTB implied a uniform distribution of elements including Cu, C, N, and O (Figure S15, Supporting Information). The XPS results revealed the full spectrum and binding energies of Cu 2p, C 1s, N 1s, and O 1s for MN@CTB. As displayed in Figure 5B, four characteristic peaks for C, N, O, and Cu were found. The high‐resolution spectrum of Cu 2p revealed the characteristic peaks at 935.8 eV (Cu 2p_3/2_) and 955.6 eV (Cu 2p_1/2_) (Figure 5C), indicating the presence of Cu^2+^ in MN@CTB.^[^ ^27^ ^]^ Besides, the XPS results exhibited typical peaks of O─C ═ O (288.8 eV) and C─O/C─N (285.3 eV) in the C 1 s spectrum (Figure 5D), the peaks of N─N (407.1 eV) and ─N ═ O (400.6 eV) in the N 1 s spectrum (Figure 5E), and the peak of Cu─O (533.9) and O─H (531.7 eV) in the O 1 s spectrum(Figure 5F), which implied the existence of CTB nanozymes in MN@CTB microneedle patches.^[^ ^27^ ^]^

Subsequently, the photothermal effect, NO release profile, antioxidant capacity, and antibacterial properties of the MN@CTB microneedle patches were investigated. As shown in Figure 5G, the 0.5 W cm^─2^ NIR light could elevate the MN@CTB temperature to 40.5 °C within 10 min. In comparison, the MN@CTB temperature increased to 45.9 and 53.2 °C, with the NIR light intensity ranged from 1.0 W cm^─2^ to 1.25 W cm^─2^. Given these findings and the potential risk of elevated temperatures, 1.0 W cm^─2^ was employed as the appropriate NIR power‐intensity. The NIR‐triggered NO release profiles of the MN@CTB microneedle patches under various conditions were evaluated by UV–vis spectrum. The cumulative NO release of the MN@CTB microneedle patches under NIR irradiation was ≈23.9 µm, which was higher than that without NIR irradiation (4.1 µm) within 30 min (Figure 5H).

Drug‐resistant bacterial infection and accompanying persistent oxidative stress could induce the generation of ROS in the infected wound tissues, which leads to irreversible damage to DNA/RNA and enzyme vitalities, and delays the infected wound healing process.^[^ ^33^ ^]^ Accordingly, the antibacterial ability of MN@CTB microneedle patches was evaluated using the plate colony‐counting approach. Importantly, compared with MN and MN@CTB groups, the antibacterial ratio of MN@CTB + NIR group was significantly increased (Figure 5I). The MN@CT and MN@CT + NIR groups were employed to further investigate the antibacterial effect of Cu^2+^, NO, and hyperthermia in MN@CTB microneedle patches. Compared with MN group, the antibacterial capacity of MN@CT group was significantly enhanced, which could be due to the release of Cu^2+^ in MN@CT microneedle patches. Meanwhile, the antibacterial ratio of MN@CT + NIR group (77.2%) was significantly higher than that of MN@CT (41.1%), indicating that the NIR‐induced local hyperthermia could kill bacteria effectively. After loading of BNN6, the antibacterial capacity of MN@CTB + NIR group was 94.7%, while the antibacterial capacity of MN@CT + NIR group was 77.2% (Figure S16, Supporting Information), suggesting that the synergistic effect of photothermal capacity and NIR light‐controlled release of NO could effectively inhibit the MRSA viability. Similarly, the MN@CTB microneedle patches exhibited strong antibacterial effects against *E. coli* and *S. aureus*, respectively (Figure S17, Supporting Information). According to our previous studies, the plentiful phenol groups on TA molecules endow it possess excellent antioxidant capacity, which assures the antioxidant effect of MN@CTB microneedle patches.^[^ ^34^ ^]^ The DPPH scavenging and ABTS^•+^ scavenging activities were significantly improved, with scavenging percentages of 31.2 for DPPH and 44.6% observed in the MN@CTB group (Figure 5J,K).

Live/dead staining and CCK‐8 assay were utilized to investigate the cytocompatibility with the microneedle patches extracts with L929 cells and HUVECs. As shown in Figure S18 (Supporting Information), almost all of the L929 cells were alive (green) after 4 days of incubation and dead cells (red) rarely appeared in all groups. The results implied the good cytocompatibility of the MN and CTB@MN groups, as further validated by the CCK‐8 assay (Figure S19, Supporting Information). Similarly, the CTB@MN microneedle patches also showed good cytocompatibility toward HUVECs (Figures S20 and S21, Supporting Information). The migration of HUVECs cultured with various microneedle patches extractions was investigated by transwell co‐culture system. The results revealed that the CTB@MN group was able to promote the migration of HUVECs (Figure S22, Supporting Information), revealing the presence of Cu^2+^ and TA played a potential role in improving the recruitment of HUVECs.^[^ ^35^ ^]^

### 2.7 Antioxidant, Anti‐Inflammatory, and Angiogenic Mechanisms of MN@CTB Microneedle Patches

**Figure 6:**
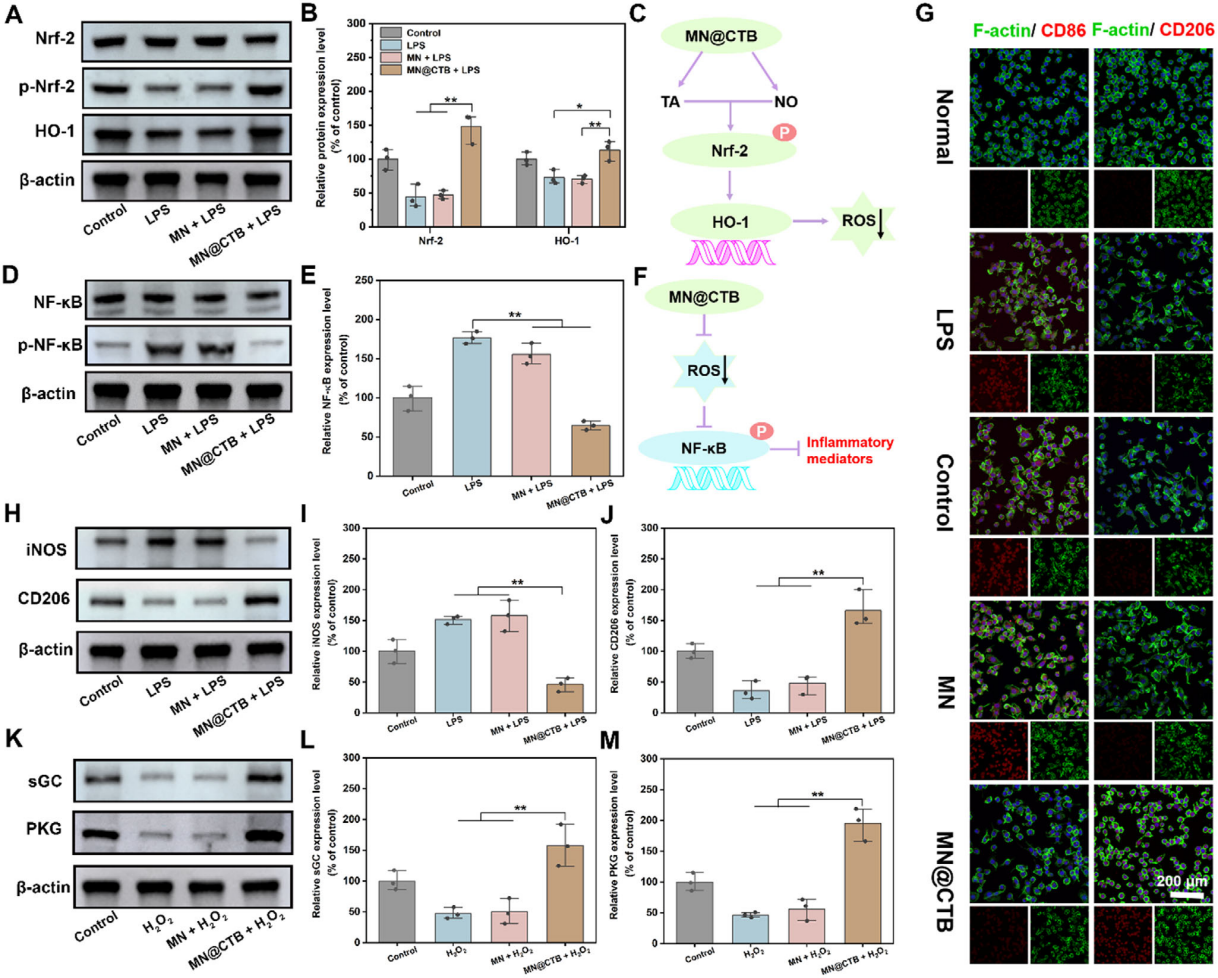
Antioxidant, anti‐inflammatory, and angiogenic mechanisms of MN@CTB microneedle patches. A) WB assay on the expression level of Nrf‐2 and HO‐1 proteins. B) The quantitative analysis of Nrf‐2 and HO‐1 protein (*n* = 3). E) Schematic illustration of the mechanism of MN@CTB activating the Nrf‐2 and HO‐1 signaling pathways against ROS. D) The protein expression level of NF‐κB was determined by western blot. C) Relative expression of NF‐κB in macrophages after different treatments (*n* = 3). F) Schematic illustration of the mechanism of MN@CTB inhibiting the NF‐κB signaling pathway to downstream inflammatory factor expression. G) CD86 and CD206 immunofluorescence staining of RAW 264.7 cells on different samples. H) The protein expression level of iNOS and CD206 in macrophages was determined by western blot. I,J) The quantitative analysis of iNOS and CD206 proteins (*n* = 3). K) The expression level of sGC and PKG proteins in HUVECs after different treatments. L,M) Relative expression of sGC and PKG proteins in each group (*n* = 3), ^*^ *p* < 0.05, ^**^ *p* < 0.01.

The Nrf‐2 signaling pathway is a well‐established antioxidative mechanism in macrophages and can be weakened by the persistent oxidative stress induced by ROS.^[^ ^30^ ^]^ Previous studies confirmed that NO and TA can activate Nrf‐2 and promote the heme expression of oxygenase‐1 (HO‐1), thus coupling to eliminate ROS in the oxidative stress environment.^[^ ^35^ ^]^ Immunofluorescent staining results showed that Nrf‐2 and HO‐1 signaling pathways of macrophages in the control, LPS, and MN groups were not activated. In comparison, the fluorescence intensity of Nrf‐2 and HO‐1 in the MN@CTB group was remarkably increased, implying a substantial upregulation of Nrf2 and HO‐1 (Figure S23, Supporting Information). Western blot assay indicated that the expression levels of Nrf‐2 and HO‐1 were significantly decreased with lipopolysaccharide (LPS) treatment, suggesting that the cellular antioxidative mechanism was weakened (**Figure** **6**A). However, with the treatment of MN@CTB, the expression levels of Nrf‐2 and HO‐1 were significantly increased compared with LPS and MN groups (Figure 6B). The aforementioned results reveal that the upregulations of the Nrf‐2 and HO‐1 signaling pathways could be attributed to the strong ROS scavenging capacities caused by the synergistic effect of TA and NO (Figure 6C).

As a pivotal transcriptional factor, NF‐κB plays a vital role in innate immunity and inflammatory cascades.^[^ ^35^ ^]^ The NF‐κB signaling pathway can be activated by ROS, and excessive ROS can upregulate NF‐κB signing by inducing the translocation of NF‐κB dimers to the nucleus to initiate transcription of multiple genes, thereby promoting the expression levels of pro‐inflammatory mediators.^[^ ^36^ ^]^ Immunofluorescent staining results indicated that treatment of LPS can induce the nuclear translocation of the NF‐κB, suggesting LPS activates NF‐κB signaling pathway (Figures S24 and S25, Supporting Information). In the MN group, the fluorescence of NF‐κB remains exhibited high expression levels, indicating that MN microneedle patches could not decrease the NF‐κB signaling pathway. In comparison, the expression levels of NF‐κB were significantly suppressed due to the phenomenon of lower fluorescence and the inhibition of NF‐κB translocation in the MN@CTB group. Western blot assay demonstrated that LPS treatment potently stimulated an up‐regulation of NF‐κB compared with the control group, suggesting that the cellular inflammatory cascades were activated. After treatment with MN@CTB microneedle patches, NF‐κB expression was significantly lower than in the LPS and MN groups, with a relative ratio of 64.5% (Figure 6D,E). These findings revealed that MN@CTB microneedle patches could inhibit the expression of NF‐κB signaling pathway, induce macrophage polarization, and modulate the inflammatory mediators' expression. It was indicated that the MN@CTB microneedle patches remarkably decreased ROS levels in RAW264.7 cells, subsequently suppressing the activation of the NF‐κB pathway (Figure 6F), and down‐regulating macrophage polarization toward M1 phenotype and inflammatory mediators expression.

The infected chronic wounds, apart from inhibiting bacterial infection and ROS clearance, face the challenge of effectively modulating the pro‐inflammatory phenotype (M1‐type) to the anti‐inflammatory phenotype (M2‐type), and ameliorating the imbalance of immune regulation and suboptimal angiogenesis.^[^ ^36^ ^]^ Consequently, the modulation effects of these microneedle patches on macrophage phenotype were investigated by immunofluorescence staining for CD86 (an M1‐type macrophage marker) and CD206 (an M2‐type macrophage marker). After LPS induction, RAW264.7 macrophages were incubated with different microneedle patches for 24 h. Notably, the MN@CTB group showed significantly reduced CD86 (red) expression compared to the normal, LPS, control, and MN groups, while CD206 (red) expression remarkably increased (Figure 6G). To further elucidate the anti‐inflammatory effects of the MN@CTB microneedle patches, the expression levels of inflammation‐related cytokines in RAW264.7 cells were quantified using cytokine secretion array, WB, and ELISA. We evaluated 20 common inflammatory cytokines at the protein level by a mouse cytokine array, and all data were normalized and graphically represented as a heat map (Figure S26, Supporting Information). The MN@CTB group presented a remarkedly distinct cytokine secretion profile, particularly for GM‐CSF, IFN‐𝛾, IL‐1𝛼, IL‐1𝛽, IL‐2, IL‐4, IL‐5, IL‐6, IL‐9, IL‐10, IL‐13, TNF‐𝛼, and VEGF. Importantly, MN@CTB significantly dampened the expression of pro‐inflammatory cytokines (IFN‐𝛾, IL‐1𝛼, IL‐ IL‐1𝛽, IL‐2, IL‐6, and TNF‐𝛼), and efficaciously improved the expression of anti‐ inflammatory cytokines (IL‐10 and VEGF). In MN@CTB microneedle patches, the expression level of pro‐inflammatory cytokine, IL‐1β, was statistically lower than those in LPS, control, and MN groups. Conversely, the concentration of anti‐inflammatory factors, such as IL‐10, was significantly enhanced (Figure S27, Supporting Information). Western blot results indicated that the iNOS (M1 marker) expression levels in the MN@CTB group were remarkably down‐regulated, while the expression levels of CD206 (M2 marker) were significantly up‐regulated (Figure 6H). Notably, quantitative analysis of the WB results revealed that iNOS levels in the MN@CTB group were 56.5% lower than the control group, while the CD206 levels were 166.4% (Figure 6I,J). These results validate the hypothesis that the phenol hydroxyl group of TA in the MN@CTB discloses efficacy in scavenging ROS, promoting macrophage polarization through the secretion of inflammatory cytokines,^[^ ^30^ ^]^ thereby alleviating the inflammatory reactions, which is advantageous for accelerating the infected wound healing process.

In the wound healing process, angiogenesis is crucial for guaranteeing the transportation of nutrients and oxygen to the wound sites to reprogram the wound microenvironment, which is advantageous for repair.^[^ ^37^ ^]^ Elevated NO expression activates downstream cascades that synergistically promote angiogenesis during wound healing. However, NO can react with ROS to generate reactive peroxynitrite (ONOO^−^) molecules, which have harmful effects on the angiogenic capacities of HUVECs. Consequently, we tested whether MN@CTB microneedle patches could significantly activate downstream signaling in HUVECs under oxidative stress conditions.^[^ ^38^ ^]^ It was demonstrated that the relative expression of soluble guanylyl cyclase (sGC) and protein kinase G (PKG) in HUVECs significantly down‐regulated after H_2_O_2_ treatment due to the elevation in oxidative stress, while the addition of MN@CTB microneedle patches could remarkably up‐regulate the expression levels of sGC and PKG, compared with the control group (Figure 6K–M). Moreover, the expression of CD31 and VEGF in HUVECs was investigated by immunofluorescence staining, qRT‐PCR, ELISA, and Western blot assay, respectively. The immunofluorescence staining images showed that the MN@CTB microneedle patches significantly increased CD31 and VEGF expression in HUVECs after various interventions over a period of 2 days (Figure S28, Supporting Information). The mRNA expression of CD31 and VEGF in MN@CTB microneedle patches was statistically higher compared to those in the control and MN groups (Figure S29A, Supporting Information). As shown in Figure S29B,C (Supporting Information), the relative expression of CD31 and VEGF was further investigated by WB analysis. In the MN@CTB group, the expression of VEGF and CD31 was significantly increased, which was in great agreement with immunofluorescence staining results (Figure S28, Supporting Information). Those upregulations of angiogenic‐related cytokines implied that MN@CTB microneedle patches were conducive to angiogenesis of HUVECs.

### 2.8 In Vivo Wound Healing of MN@CTB Microneedle Patches

**Figure 7:**
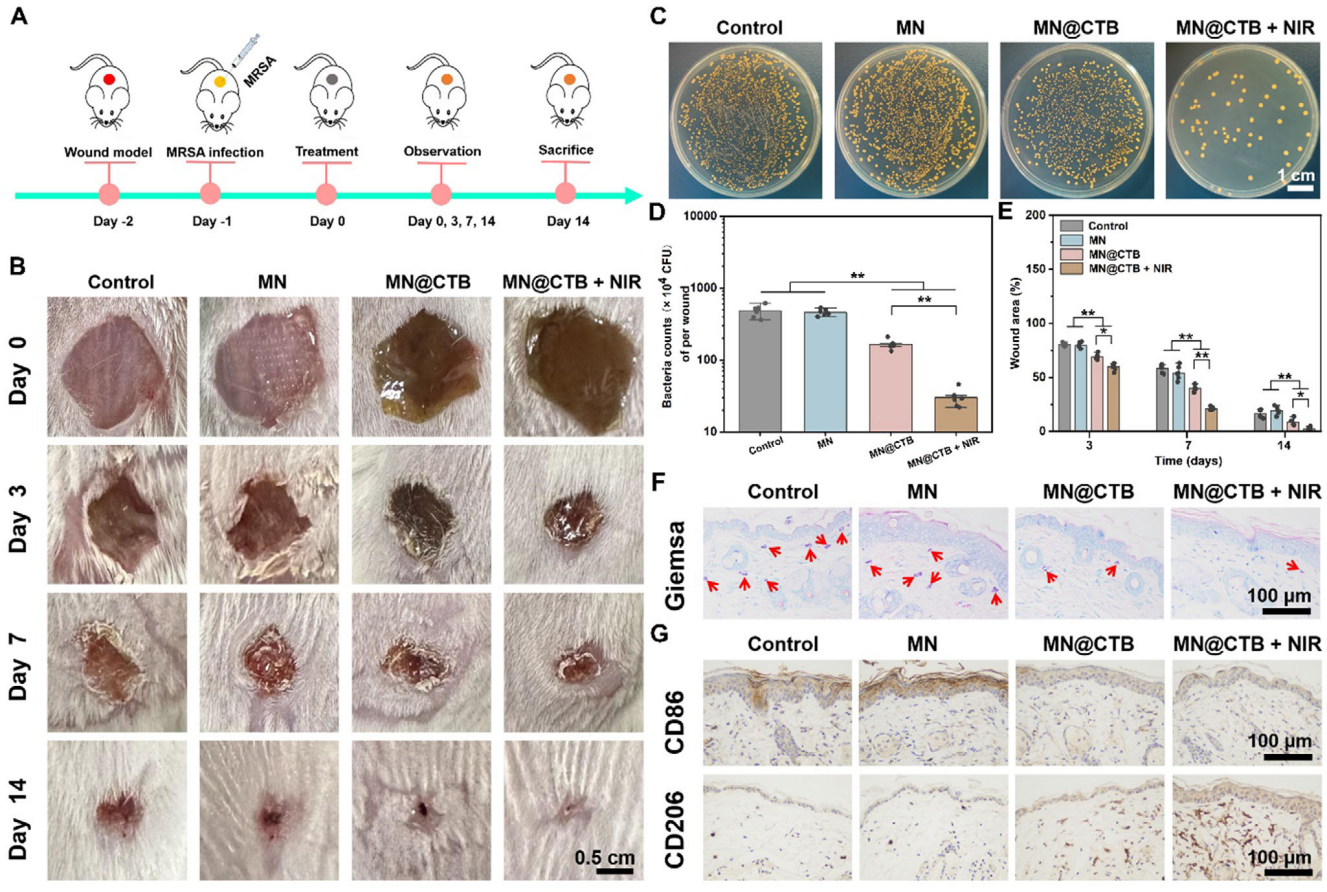
In vivo antibacterial and anti‐inflammatory capacities of MN@CTB microneedle patches for accelerating MRSA‐infected cutaneous wounds. A) Experimental timeline of the establishment of the MRSA‐infected wounds and infected wound healing. B) Representative images of the healing process of wounds in various groups. C) Photographs of bacterial colonies by standard spread plate assay were stripped from the infected wounds after different treatments on day 3. D) Statistical analysis of bacteria number of wound tissues after various treatments on day 3 (*n* = 6). E) Quantification of the remaining wound area after different treatments during the healing process (*n* = 6). F) Representative Giemsa staining image of wound tissues (red arrows) on day 3. G) Immunohistochemistry staining images of the wound sections on day 7 with CD86 and CD206 in each group, ^*^ *p* < 0.05, ^**^ *p* < 0.01.

After demonstrating the excellent antibacterial capacity of the fabricated MN@CTB microneedle patches in vitro, we evaluated their therapeutic effects in animal models.^[^ ^38^ ^]^ Specifically, the antimicrobial properties and wound healing efficacy of these microneedle patches were tested in a mouse model with full‐thickness skin defects infected with MRSA (**Figure** **7**A). After 2 days, the MRSA‐infected mice were randomly assigned to four groups: 1) no treatment (control), 2) MN patches (MN), 3) MN patches loaded with CTB nanozymes (MN@CTB), and MN patches loaded with CTB nanozymes (MN@CTB) under NIR irradiation (MN@CTB + NIR). Representative real‐time thermal images and the relevant photothermal heating curves of the control and MN@CTB groups under NIR irradiation were exhibited in Figures S30 and S31 (Supporting Information), respectively. It was revealed that the local temperature of the MN@CTB + NIR group increased rapidly from 31.1 to 40.5 within 4 min, and was maintained at 45.3 °C until 10 min, suggesting the favorable photothermal performance of the microneedle patches for infected wound healing.^[^ ^39^ ^]^ As shown in Figure 7B, these images of the wounds were captured to investigate the therapeutic effect of each group on 0, 3, 7, and 14 days. Notably, compared with other groups, the MN@CTB and MN@CTB + NIR groups exhibited smaller wound sizes on day 3. Meanwhile, the effectiveness of MN@CTB in fighting bacteria was assessed by retrieving the remaining bacteria from infected wounds using the standard plate count method.^[^ ^40^ ^]^ The antibacterial effectiveness against MRSA in the MN, MN@CTB, and MN@CTB + NIR groups corresponded closely with the in vitro antibacterial results (Figure 7C,D), implying the synergistic effect combining NO‐mediated PTT and NO gas therapy for eliminating MRSA. On day 7, the relative wound area (%) for MN@CTB and MN@CTB + NIR were 39.9% and 21.1%, which were statistically lower than those of other groups (Figure 7E). Following a 14‐day treatment period, the infected wounds treated with MN@CTB + NIR presented complete healing, those treated with MN@CTB displayed substantial healing, whereas those treated with MN alone exhibited minimal improvement. In the control and MN groups, a larger amount of residual bacteria (red arrows) was observed with Giemsa staining, showing a reduction of 65% in bacterial counting for the MN@CTB group and ≈89% reduction in the MN@CTB + NIR group (Figure 7F; Figure S32, Supporting Information). The MN@CTB + NIR group exhibited a robust antibacterial effect in vivo, which was attributed to the synergistic impact of NO‐mediated PTT and NO gas therapy for MRSA elimination.

**Figure 8:**
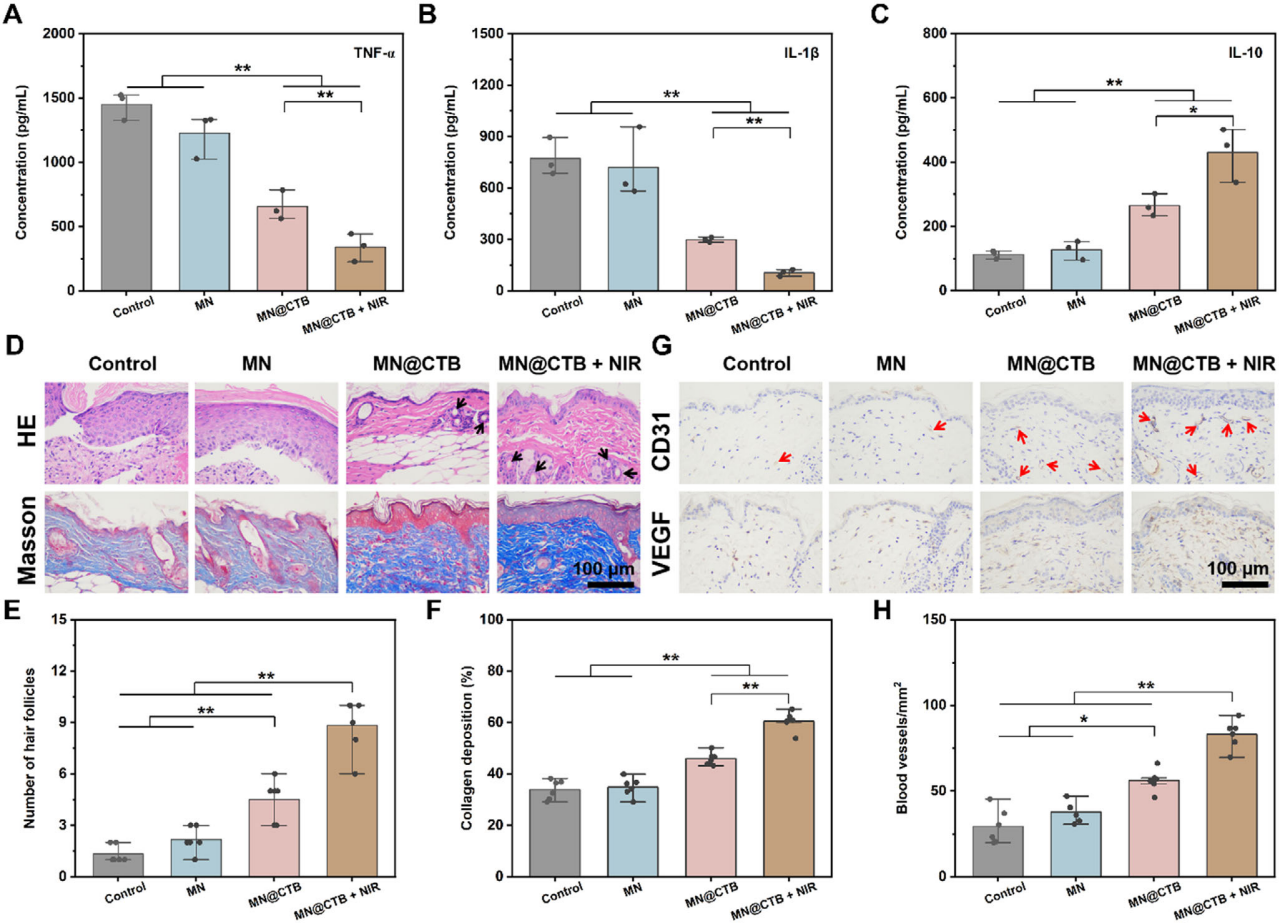
In vivo wound healing efficacy of the MN@CTB microneedle patches. A–C) ELISA of TNF‐α, IL‐1β, and IL‐10 from the infected wounds (*n* = 3). D) Representative H&E and Masson's trichrome staining image of regenerated skin with various treatments. Black arrows indicate the hair follicle in the H&E staining. E,F) The corresponding quantification of hair follicles (*n* = 6) and collagen deposition (n = 6), respectively. G) Images of immunohistochemistry staining labeling of the wound sections with CD31 and VEGF. Red arrows indicate the neovascularization of CD31‐ immunohistochemistry staining. H) Quantitative analysis of the intensity of blood vessels (*n* = 6), ^*^ *p* < 0.05, ^**^ *p* < 0.01.

The healing process of infected wounds often features a severe and persistent inflammatory response caused by the over‐activation of M1‐type macrophages, which can lead to undesirable outcomes in wound repair.^[^ ^41^ ^]^ In comparison, promoting macrophages from the M1 to the M2 phenotype and reprogramming of the inflammatory microenvironment were beneficial for tissue repair and regeneration. Consequently, the expression of M1 and M2‐type macrophages was analyzed by immunohistochemistry staining.^[^ ^42^ ^]^ In Figure 7G and Figure S33 (Supporting Information), the expression levels of CD86 and CD206 exhibited no statistical difference between the control and MN groups. However, the MN@CTB + NIR‐treated group presented a low level of CD86 and high expression of CD206, indicating a weak inflammatory response due to the MRSA elimination by the synergistic impact of NO‐medicated PTT and NO gas therapy. In addition, concentrations of the pro‐inflammatory cytokine (TNF‐α and IL‐1β) and anti‐inflammatory cytokines (IL‐10 and TGF‐β) in the regenerated skin tissues were determined by the ELISA on day 7. Infected wounds treated with MN@CTB + NIR group exhibited significant inhibition of pro‐inflammatory cytokine expression, thereby enhancing the expression of anti‐inflammatory cytokines (**Figure** **8**A–C; Figure S34, Supporting Information). The aforementioned results demonstrated that the MN@CTB + NIR alleviates inflammatory reaction by promoting the transformation of macrophages from a pro‐inflammatory to an anti‐inflammatory phenotype, thereby reprogramming the inflammatory microenvironment for infected wound healing.

Moreover, the effectiveness of infected cutaneous wound regeneration was assessed using skin tissues collected from wound sites, which underwent histomorphological analysis through hematoxylin‐eosin (HE), Masson's trichrome, and immunohistochemistry staining.^[^ ^43^ ^]^ The MN@CTB + NIR group presented obvious sectional reepithelialization accompanied by more hair follicles (black arrows) of the wound (Figure 8D,E), revealing the significant efficiency of promoting wound healing. Furthermore, MN@CTB + NIR‐treated wounds presented an organized skin layer structure with nascent and orderly‐arranged collagen deposition (Figure 8D). Especially, the collagen deposition (%) in the control, MN, MN@CTB, and MN@CTB + NIR was 33.9%, 34.6%, 46.0%, and 60.5%, respectively (Figure 8F). Collagen deposition was further analyzed by measuring the hydroxyproline (HYP) content, a commonly used marker for evaluating wound healing.^[^ ^44^ ^]^ Compared with control, MN, and MN@CTB groups, the HYP concentration in the MN@CTB + NIR group was remarkably increased (Figure S35, Supporting Information). These findings suggest that the synergistic effect of NO‐medicated PTT and NO gas therapy of MN@CTB is able to accelerate infected wound healing by suppressing the inflammatory response and enhancing tissue regeneration. In addition, considering the significant role of angiogenesis on wound tissue regeneration, the expression of CD31 and VEGF was detected by immunohistochemistry staining.^[^ ^45^ ^]^ After 7 days of healing, more blood vessels (marked with CD31, red arrows) were found in the MN@CTB and MN@CTB + NIR groups. Similarly, lower VEGF expression was observed in the control and MN groups, however, the highest VEGF expression was found in the MN@CTB + NIR group (Figure 8G). Noticeably, MN@CTB + NIR‐treated group presented the highest blood vessel density (83.1 blood vessels/mm^2^) as compared to control (29.3 blood vessels/mm^2^), MN (37.8 blood vessels/mm^2^), MN@CTB (56.2 blood vessels/mm^2^) (Figure 8H). Moreover, blood flow at the wound sites in mice suggested that MN@CTB + NIR group presented the higher blood perfusion volume (PU) (Figure S36, Supporting Information), which was attributed to the fact that NO‐medicated PTT and NO gas therapy were conducive to promoting angiogenesis and promoting wound healing.

### 2.9 Pro‐Healing Mechanism of MN@CTB Microneedle Patches

**Figure 9:**
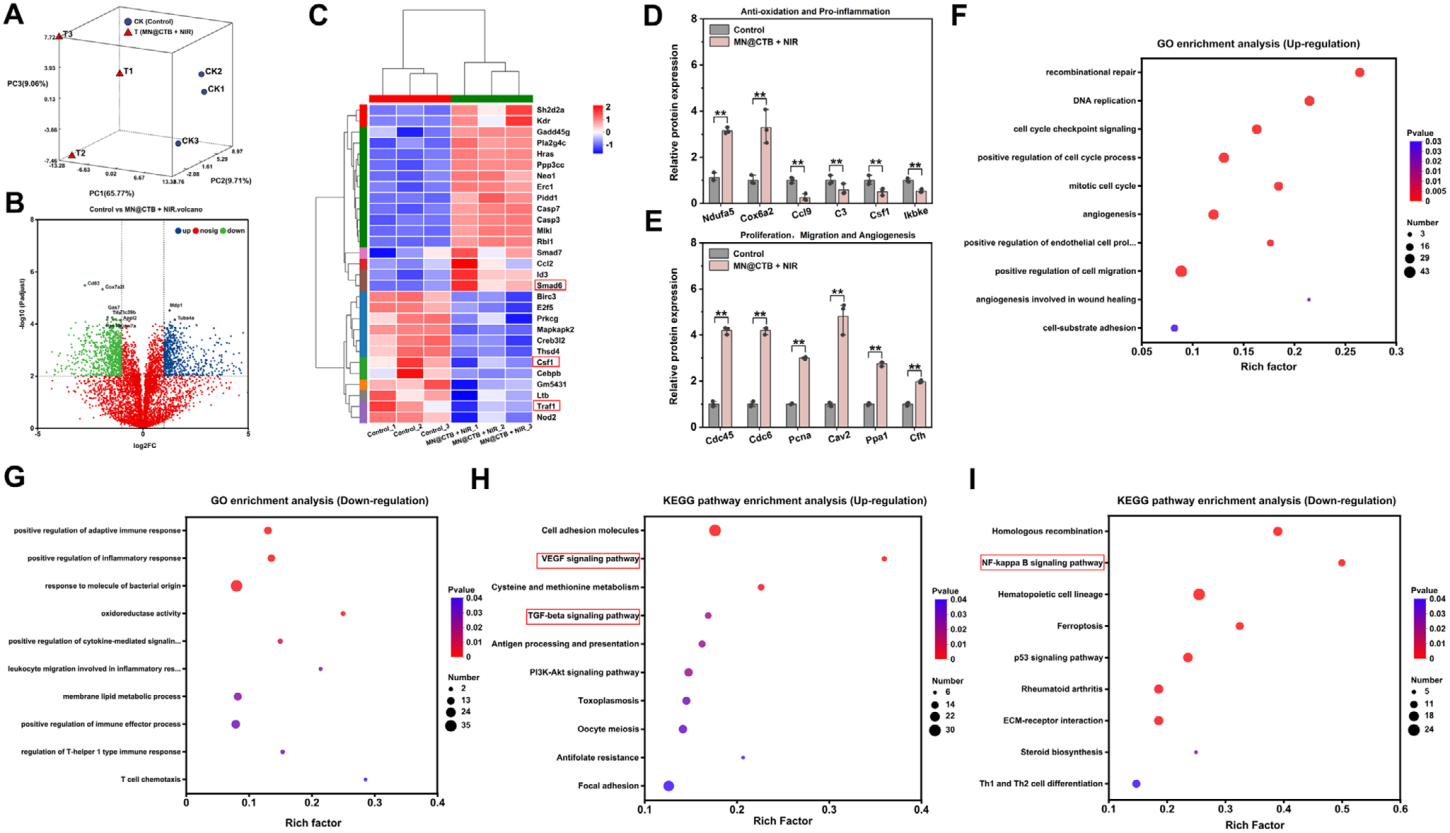
Pro‐healing mechanism of the MN@CTB microneedle patches. A) Principal component analysis (PCA) of a distinct separation between the groups based on the proteins expressed differently (*n* = 3). B) Volcano plot showing upregulated and downregulated proteins after treatment with the MN@CTB microneedle patches. C) A heatmap displaying proteins expressed differently in the control and MN@CTB groups (*n* = 3). D,E) Relative expression of proteins related to biological functions, including oxidative stress, inflammation, cell proliferation, migration, and angiogenesis (*n* = 3). F,G) Gene Ontology (GO) enrichment analysis of up‐regulated and downregulated proteins in MN@CTB + NIR‐treated wounds versus control wounds. H,I) Kyoto encyclopedia of genes and genomes (KEGG) pathway analysis of differentially expressed proteins related to molecular signaling pathways in MN@CTB + NIR‐treated wounds versus control wounds, ^*^ *p* < 0.05, ^**^ *p* < 0.01.

To further delve into the pro‐healing mechanisms of wound healing associated with MN@CTB microneedle patches, proteomic analysis was conducted.^[^ ^46^ ^]^ Protein expression disparities between the control and MN@CTB + NIR groups were evident in **Figure** **9**A, as delineated by principal component analysis (PCA). As shown in Figure 9B, various protein expressions in mice treated with MN@CTB microneedle patches + NIR compared with the control group, as depicted in a volcano plot. The heat map presents significant changes in protein expression, highlighting that anti‐inflammatory mediators such as Smad6 were highly expressed. Conversely, the pro‐inflammatory mediators like Traf1 and Csf1 were reduced in the MN@CTB + NIR group (Figure 9C). According to the relative expression of protein analysis, the potential therapeutic effects of MN@CTB + NIR treatment may stem from the upregulation of antioxidation‐related proteins such as Ndufa5 and Cox6a2, and the down‐regulation of inflammatory‐related proteins like Ccl9, C3, Csf1, and Ikbke (Figure 9D). Furthermore, compared with the control group, proteins related to cell proliferation, migration, and angiogenesis, including Cdc45, Cdc6, Pcna, Cav2, Ppa1, and Cfh, were significantly upregulated in the MN@CTB + NIR group (Figure 9E). Additionally, Gene ontology (GO) enrichment analysis demonstrated that the reregulated proteins were associated with the functions of recombinational repair, positive regulation of cell migration, and angiogenesis involved in wound healing, In contract, the downregulated proteins were linked to inflammatory reactions, including the positive regulation of inflammatory response, response to molecule of bacterial origin, and positive regulation of immune effector process (Figure 9F,G). The Kyoto encyclopedia of genes and genomes (KEGG) pathway analysis suggested that treatment of MN@CTB + NIR activated the VEGF and TGF‐β signaling pathways while inhibiting the NF‐κB signaling pathway (Figure 9H,I). The results indicate that MN@CTB microneedle patches can modulate inflammation responses by inhibiting the NF‐κB signaling pathway, which helps to normalize the pathological microenvironment and improves angiogenesis via VEGF and TGF‐β signaling pathways, ultimately accelerating wound healing.

### 2.10 Biosafety of MN@CTB Microneedle Patches

Clinical anaphylaxis symptoms were intuitive indicators that reflected the degree of allergy, including allergy scores and body surface temperatures.^[^ ^47^ ^]^ In contrast, with MN@CTB microneedle patches treatment, no significant differences were found in allergy symptom score, body surface temperature, IgE, and HIS concentration in mice (Figure S37A–D, Supporting Information), which indicated that MN@CTB microneedle patches do not induce an allergic reaction. Moreover, the inductively coupled plasma (ICP) assay was estimated to determine the content of Cu^2+^ in the main organs and blood of MRSA‐infected wound mice.^[^ ^47^ ^]^ The ICP detection results suggested that MN@CTB microneedle patches with no obvious toxicity (Figure S37E, Supporting Information). A hemolysis assay was performed to investigate the hemocompatibility of MN@CTB microneedle patches, using TritonX‐100 as the positive control and the PBS as the negative control. Compared with PBS group, MN@CTB microneedle patches presented a similar extent of hemolysis rate (%) (Figure S38A, Supporting Information). The biochemical indicators of CREA, ALB, ALT, ALP, AST, and UREA in the MN@CTB group remained with normal levels (Figure S38B–G, Supporting Information), and the hematological parameters felled with normal limits (Figure S38I–M, Supporting Information). Furthermore, no pathological abnormalities in major organs were observed in the MN@CTB group (Figure S38N, Supporting Information). These results revealed that MN@CTB microneedle patches exhibited low toxicity and potential biosafety, which is advantageous for therapeutic applications for enhanced wound healing.

## 3 Conclusion

In summary, we have developed versatile microneedle patches (MN@CTB) to enhance wound healing and skin tissue regeneration within an MRSA‐infected cutaneous wound model. The synergistic effect of NO‐mediated PTT and NO gas therapy from the MN@CTB microneedle patches induces the demise of MRSA through the generation of reactive oxygen species (ROS), alterations in membrane permeability, K^+^ efflux, ATP reduction, ONPG hydrolysis, and leakage of intracellular components (DNA/RNA and proteins). Moreover, the MN@CTB microneedle patches exhibit superior photothermal conversion effects, controlled release of NO gas, antioxidant properties, and the capacity to restore intracellular redox homeostasis. Additionally, these patches expedite wound healing and tissue regeneration by mitigating oxidative stress, promoting M2 macrophage polarization, enhancing neovascularization, and facilitating hair follicle regeneration and collagen deposition. The proteomic analysis indicated that the MN@CTB microneedle patches primarily inhibited the NF‐𝜅B, while activating the VEGF and TGF‐β signaling pathways, leading to anti‐inflammatory effects and improving angiogenesis to promote infected wound healing. Overall, the microneedle patches engineered to address the specific characteristics of MRSA‐infected cutaneous wounds and hold significant promise for the clinical management of bacterial‐associated wounds.

## Conflict of Interest

The authors declare no conflict of interest.

## Supporting information



Supporting Information

## Data Availability

The data that support the findings of this study are available from the corresponding author upon reasonable request.
