# Supplementary material for: Multifunctional NIR‐Triggered Nanozyme‐Based Microneedles for Synergistic Eradication of MRSA and Enhanced Wound Healing
Source: Adv Sci (Weinh). 2025 Jul 23;12(38):e10774. doi: 10.1002/advs.202510774 (PMC12520562; doi:10.1002/advs.202510774)
Supplement: Supplementary file 1 — Supporting Information [file ADVS-12-e10774-s001.docx]

**Multifunctional NIR-Triggered Nanozyme-Based Microneedles for Synergistic Eradication of MRSA and Enhanced Wound Healing**

*Wei Qian^#^, Ruixi Li^#^, Xiyan Zheng^#^, Yingliang Li, Haiwei Xiong, Ye Zhang, Dengliang Lei, Qingfeng Shi, Yufeng Xie, Yiting Zhou, Bailong Tao*, Kuai Yu*, Aiping Le*, Boxuan Zhou**

Y. Li, H. Xiong, Q. Shi, Y. Xie, Y. Zhou,Prof. B. Zhou

Department of Breast Disease Center, General Surgery, the 1^st^ Affiliated Hospital, Jiangxi Medical College, Nanchang University, Nanchang, 330006, PR China

1. mail: [zhoubx@ncu.edu.cn](mailto:zhoubx@ncu.edu.cn)

Prof. A. Le, Prof. K. Yu

Department of Blood Transfusion, Key Laboratory of Jiangxi Province for Transfusion Medicine, the 1^st^ Affiliated Hospital, Jiangxi Medical College, Nanchang University, Nanchang,330006, PR China

E-mail: ndyfy00973@ncu.edu.cn, yukuai1949@foxmail.com

W. Qian

Department of Cardiology, the 1^st^ Affiliated Hospital, Jiangxi Medical College, Nanchang University, Nanchang, 330006, PR China

R. Li, X. Zheng

Department of Hepatobiliary and Pancreatic Surgery, the Eighth Affiliated Hospital, Sun Yat-sen University, Shenzhen, 518033, PR China

Y. Zhang, D. Lei, B. Tao,

Laboratory Research Center, the First Affiliated Hospital of Chongqing Medical University, Chongqing, 400016, PR China

E-mail: taobailong@hospital.cqmu.edu.cn

Prof. B. Zhou

Postdoctoral Innovation Practice Base, the 1^st^ Affiliated Hospital, Jiangxi Medical College, Nanchang University, Nanchang, 330006, PR China

**Experimental methods**

*Materials**:* N,N'-di-sec-butyl-p-phenylenediamine (BPA), sodium nitrite (NaNO2), N-Phenyl-1-naphthylamine (NPN) were sourced from Aladdin Biochemical Technology Co., Ltd (Shanghai, China). Cupric sulfate, tannic acid, methacrylate anhydride, hyaluronic acid (HA, Mw = 150000–200000), fluorescein diacetate (FDA), and propidium iodide (PI) were purchased from Sigma-Aldrich Chemical Co. (USA). Bicinchoninic acid (BCA) assay kit, Adenosine triphosphate hematoxylin (ATP) kit, Hoechst 33258, and β-galactosidase kit were purchased from Beyotime Biotechnology Co. (Shanghai, China). CCK-8, Luria-Bertani (LB) medium, hematoxylin and eosin (H&E) staining kit, Masson’s trichrome staining kit, and Giemsa staining kit were supplied with Solarbio Co. Ltd (Beijing, China). IL-1β, IL-10, CD31, and VEGF were purchased from ABclonal Biotechnology. Co., Ltd (Wuhan, China). All reagents were used as received without further purification.

*Preparation of copper-tannic acid (Cu-TA) nanozymes**:* Cu-TA nanozymes were prepared through an oxidative coupling assembly approach. Briefly, 50 mg of tannic acid (TA) was fully dissolved in deionized (DI) water, and the CuSO4·5H2O solution (20 mL, 1.75g) was added dropwise to the TA solution. Then, the pH of the above mixture was adjusted to 7.4 by 2M NaOH and the reaction was maintained at 50 °C for 3 h. The resulting product was collected by centrifugation and washed several times with DI water to remove impurities. Lastly, the purified light green Cu-TA nanozyme was dried at 50 °C under vacuum for 24 h.

*The Synthesis and Loading of* *N, N'-di-sec-butyl-N, N'-dinitroso-1, 4-phenylenediamine (BNN6):* BNN6 was fabricated based on the protocol previously described in our study.^[1]^ First, the mixture of N,N′-bis-sec-butylamino-p-phenylenediamine (BPA) (4.68 mL, 20.0 mM) to 36 mL with ethanol. 40 mL of degassed NaNO_2_ solution (6.0 M) was stirred for 30 min under N_2_ atmosphere and 40 mL of degassed HCl (6.0 M) was added to the above mixture by a constant pressure separatory funnel. After being reacted for 4 h, the beige precipitate was collected *via* centrifugation and washed repeatedly with 50% aqueous ethanol to remove excess reagents. The BNN6 product was obtained by lyophilization under dark conditions and stored at -20 ℃ for further use. For the BNN6 loading, 5 mg Cu-TA nanozymes were dispersed in 5 mL DI water, then 2 mL BNN6 solution (0.05, 0.1, 0.15, 0.2, 0.3, 0.4, 0.5, 0.6, 0.8, and 1.0 mg/mL in ethanol) was added and stirred for 24 h under dark conditions. Lastly, the BNN6-loaded Cu-TA (CTB) nanozymes were centrifugated, rinsed with DI water, and stored at -20 ℃ for further use.

*Chemical composition and morphology characterization of CTB nanozymes:* The samples were subjected to various analytical techniques to characterize their chemical composition and observe their morphology. The chemical structure of BNN6 was characterized by high-resolution NMR (AVANCE NEO Ascend 600, Bruker, Switzerland). The chemical structures of TA, Cu-TA, BNN6, and CTB were characterized by Fourier transform infrared (FT-IR) spectroscopy (Nicolet iS50, Thermo Fisher, USA). The BNN6 loading capacity (%) was characterized by a UV-vis spectrophotometer (Lambda1050, PerkinElmer, England). The microscopic morphology of CTB sample was observed by transmission electron microscopy (TEM, LIBRA 200CS, Carl Zeiss, Germany).

*Photothermal performance assessment of CTB nanozymes:* Various concentrations of CTB nanozyme solutions were placed in a 24-well plate and irradiated with 808 nm laser (1.0 W/cm2, 10 min). The temperature change of the mixture was recorded by a digital thermometer (recorded once every 30 s). Meanwhile, the temperature change of 100 μg/mL CTB nanozyme solution under different power densities (0.25-1.25 W/cm^2^) was further recorded.^[2]^ To further evaluate the thermal stability of the CTB nanozyme, the sample was irradiated with 808 nm laser light (1.0 W/cm2, 10 min), and then the sample was cooled (10 min). This process was repeatedly evaluated for four times. Lastly, the photothermal conversion efficiency (η) of CTB was determined by the following formula, as described in our previous study.^[2]^

$\eta=\frac{hS\left( T_{max}-T_{surr} \right)-Q_{0}}{I(1-{10}^{-A808})}$ (1)

*τs =* $(m_{d}c_{d})/(hS)$ (2)

*Q0 = hS (Tmax, water - T surr)* (3)

Where h indicates the heat transfer coefficient, S represents the sample container surface area. The value of hS is calculated according to equation (2). Tmax represents the temperature change of CTB at the maximum steady-state temperature, Tmax, water is the steady state maximum temperature of water, Tsurr indicates the ambient room temperature. Q0 represents the background energy input without CTB and calculated form equation (3). I is the laser power density of the 808 nm light irradiation; A refers to the absorbance of CTB at the wavelength of 808 nm; Cs and ms are the specific heat capacity and the total mass of H_2_O, respectively; τs is the time constant quantity of CTB. Consequently, the τ was calculated from the linear regression curve in the cooling period of CTB.

*Characterization of NO release profile*: To investigate the release of NO from the CTB with/without NIR irradiation, UV-vis spectrometry was employed.^[2]^ The CTB (100 μg/mL) solution was irradiated with various power density (0.25-1.25 W/cm^2^) for 10 min, and 100 μL supernatant was obtained every 2 min by centrifugation for NO detection. After incubation of the collected supernatant with Greiss reagent under dark conditions for 10 min, the OD value was detected at the wavelength of 540 nm. Meanwhile, CTB (100 μg/mL) solution without NIR laser irradiation was also investigated with a similar approach. Similarly, the NIR light-stimulated NO release from CTB was further evaluated. The concentration of NO was determined by the standard NO calibration curve.

*Antibacterial activity test of CTB nanozymes:* Gram-negative *E. coli* (ATCC25922)*,* Gram-positive *S. aureus* (ATCC25922)*, and Methicillin-resistant Staphylococcus aureus* (MRSA, ATCC33591) was employed to evaluate the antibacterial effect of CT (without BNN6 loading) and CTB nanozymes. The antibacterial capacity of various concentrations of CTB nanozymes, various concentration (25-200 μg/mL) of CTB nanozymes solutions (150 μL) or PBS (150 μL) was mixed with bacterial suspension (50 μL, 1 × 106 CFU/mL). Then, the bacterial suspensions were irradiated with laser light (1.0 W/cm2, 10 min). Following this, suspension (100 μL) was smeared onto LB agar plates and cultured for 24 h. Lastly, the photographs of colony-forming units (CFUs) were taken and the Bacterial viability (%) was determined by the following equation: Bacterial viability (%) = m/n × 100%, where m is the average CFUs following different treatments, and n represents the average CFUs of the control group.

*Morphological observation of the bacteria:* The morphological features of the bacteria after different treatments were observed via SEM. Briefly, the bacterial suspension in various groups was collected by centrifugation (5000 rpm, 10 min), and then fixed at 4 °C overnight with 4% paraformaldehyde (PFA). The fixed samples were rinsed with PBS, and then dehydrated by gradient ethanol solutions (30%, 50%, 70%, 90%, 100%, and 100%) for 10 min. Next, 10 μL of the obtained bacterial samples were dropped on the silicon and dried in a drying oven. Finally, the dried bacterial samples were sputtered-coated with a layer of gold for SEM observation.

*Assessment of outer membrane (OM)* *permeabilization:* The OM permeabilization change of various concentrations of CTB (25, 50, 100, and 200 μg) was evaluated by the 1-N-phenylnaphthylamine (NPN) uptake assay.^[3]^ The bacterial suspension was co-cultured with different concentrations of CTB for 3 h, and the cultured medium was collected by centrifugation. Afterward, the obtained supernatant was rinsed twice with PBS and re-suspended into NaCl solution (0.5%). Therefore, the NPN probe (10 mM) was added into the bacterial suspension and co-cultured for 10 min. Lastly, the NPN fluorescence signals were determined by a fluorescence spectrophotometer (RF5301PC, Shimadzu, Japan) at the excitation wavelength of 350 nm and the emission wavelength of 420 nm, respectively.

*Detection of K^+^ out-diffusion:* Bacterial suspension was co-cultured with different concentrations of CTB nanozymes solutions (150 μL) and incubated at 37 °C for 1 day. Then, the cultured medium was collected with centrifugation, and the obtained supernatant was re-suspended into DI water (1 mL). Lastly, the amount of released K^+^ was determined by using a inductively coupled plasma optical emission spectrometry (ICP-OES, Vista AX, Varian, USA).

*Assessment of inner membrane (IM) permeabilization:* The IM permeabilization change of different concentrations of CTB was determined by o-nitrophenyl-β-d-galactopyranoside (ONPG) hydrolysis assay.^[4]^ Briefly, bacterial suspension was incubated with various concentrations of CTB nanozymes for 6 h. Afterward, the bacteria suspension was co-cultured with ONPG solution (0.5 mL, 0.75 M) for 3 h. Lastly, the absorbance of the yellow product was determined by a spectrophotometric microplate reader (Bio-Rad 680, USA) at 420 nm.

*Determination of intracellular ATP concentration**:* The intracellular ATP level of bacteria in each group was assessed using the Enhanced ATP assay kit (Beyotime, China). Briefly, bacterial suspension was cultured with various concentrations of CTB nanozymes (25-200 μg/mL) for 1 day. Thereafter, the intracellular ATP level was detected using a fluorescence spectrophotometer at the wavelength of 562 nm. The bacterial suspension without 808 nm light irradiation was used as the control group.

*Leakage of intracellular components:* The leakage of intracellular components (including proteins and nucleic acid) was investigated to evaluate the membrane integrity of bacteria.^[5]^ Briefly, bacteria (1 mL, 1 × 10^6^ CFU/mL) was cultured with different concentrations of CTB for 24 h. Afterward, the concentrations of leaked proteins were determined by the Enhanced BCA protein assay kit (Beyotime, China). Meanwhile, the concentrations of leaked DNA and RNA from bacteria were detected with a UV-Vis spectrophotometer at 260 nm. Besides, the CT (200), CTB (200), CTB (200) + NAC, and CTB (200) + EDTA groups were utilized to investigate the antibacterial effects of the individual roles (ROS, NO, Cu²⁺, and hyperthermia). Especially, the effects of ROS and Cu^2+^ was neutralized by the addition of ROS scavengers (N-acetylcysteine, NAC) and Cu²⁺ chelators (ethylene diamine tetraacetic acid, EDTA), respectively.

*Anti-oxidant activity of the CTB nanozyme:* The anti-oxidant capacity of various concentrations of CTB nanozyme was determined using 2,2-diphenyl-1-picrylhydrazyl (DPPH) free radical scavenging assay. Different concentrations of CTB nanozyme (25, 50, 100, and 200 μg/mL) were mixed with 2 mL DPPH solution (0.1 mM) at 37 ℃ for 30 min under dark conditions. The absorbance of CTB (25), CTB (50), CTB (100), and CTB (200) specimens was determined using a UV-Vis spectrophotometer (Lambda1050, PerkinElmer, England) at the wavelength of 519 nm. The DPPH scavenging activity was calculated as follows: DPPH scavenging activity (%) = (A_0_ - A_1_/A_0_) × 100%, where A_0_ indicate the absorbance of the CTB nanozyme specimen, A_1_ represent the absorbance of DPPH solution. Ascorbic acid was used as the positive control.

The ABTS^•+^-scavenging ability of various concentrations of CTB nanozyme was measured through the following method. Briefly, the ABTS^•+^ radical cations was obtained according to the manufacturer’s instructions. Therefore, the working solution was diluted and the characteristic absorbance at 734 nm was further confirmed. Afterward, the diluted mixture was incubated with various concentrations of CTB nanozyme at 37 ℃ for 1 h. Lastly, the absorbance of each group was determined by a UV–vis spectrophotometer at the wavelength of 517 nm. The scavenging activity of ABTS^•+^ free radicals was calculated using the following equation: ABTS^•+^ scavenging activity (%) = (C_0_ –C_1_)/C_0_ × 100%, where C_0_ is the absorbance of ABTS free radical solution, and C_1_ is the absorbance of samples in the experimental groups, including CTB (25), CTB (50), CTB (100), and CTB (200). Ascorbic acid was used as the positive control.

The •OH-scavenging effect was measured with the Fenton reaction between H_2_O_2_ and ferrous sulfate (FeSO_4_), and the specific probe salicylic acid (SA) was added. Next, various concentrations of CTB nanozyme was added to the above mixture and co-cultured for 30 min. The absorbance of the supernatant in each group was measured using a UV-vis spectrophotometer at the wavelength of 625 nm. The •OH scavenging property was calculated by the following equation: •OH scavenging activity (%) = (A_0_ –A_1_)/A_0_ × 100%, where A_0_ is the absorbance of the working solution without CTB nanozyme (negative control group), and A_1_ is the absorbance of samples in the experimental groups, including CTB (25), CTB (50), CTB (100), and CTB (200). Ascorbic acid was used as the positive control.

The O_2_^•−^-scavenging ability was investigated utilizing the inhibition ratio of the photo-reduction nitrotetrazolium blue (NBT). The working solution containing methionine (3.1 mM), riboflavin (5 μM), and NBT (19 μM) was mixed, and then various concentrations of CTB nanozyme was added to the above mixture. Therefore, the mixture was exposed to UV irradiation (5 min), and the absorbance of the supernatant of each sample was determined at the wavelength of 560 nm. The O_2_^•−^ scavenging activity was calculated according to the following equation: O_2_^•−^ scavenging activity (%) = (A_1_ – A_0_)/(A_1_ – A_2_) × 100%, in which A_0_ is the absorbance of the working solution without UV irradiation (negative control group), A_1_ indicates the absorbance of the working solution with UV irradiation (positive control group), and A_2_ represents the absorbance of the sample in the experimental group, including CTB (25), CTB (50), CTB (100), and CTB (200). Ascorbic acid was used as the positive control.

*In vitro evaluation of* *ROS-scavenging activity of CTB nanozymes:* The mouse fibroblasts (L929) were cultured with 6-well plates for 1 day, and then H_2_O_2_ (0.1 mM) was added to increase intracellular oxidative stress for 6 h.^[6]^ The cells were incubated with different concentrations of CTB nanozymes for 12 h. Then, the L929 cells were treated with 2’,7’-dichlorodihydrofluorescein diacetate (DCFH-DA) under dark conditions. Lastly, the fluorescence photographs of the L929 cells were imaged using laser confocal microscopy (Olympus, Japan), and the relative ROS intensity was further analyzed.

*Cell viability assay of CTB nanozymes*: After different treatments, the L929 cells were washed with PBS twice, fixed with 4% paraformaldehyde (PFA), permeabilized with Triton X-100 (0.2%), and stained with FITC–phalloidin for cytoskeleton (red) and DAPI for cell nuclei (blue), respectively. The stained cells were observed using a confocal laser scanning microscope (CLSM, TCS. SP8, Leica, Germany). Besides, the cell viability of various concentrations of CTB nanozymes was investigated by CCK-8 assay after incubation of 24 and 48 h. Briefly, the medium was withdrawn and an aliquot of prepared CCK-8 solution (10 μL) with fresh medium (90 μL) was added to each sample within different treatments. After another 2 h of incubation, the OD value of each sample was measured at 450 nm using a spectrophotometric microplate reader. The cell viability (%) was calculated according to the following equation: Cell viability (%) = (ODe - ODc) / ODc × 100%, where ODe and OD_C_ represent the optical density value of the experimental and control group, respectively.

*Scratch assay of* *CTB nanozymes:* The impact of different concentrations of CTB nanozymes on the migration of human umbilical vein endothelial cells (HUVECs) was investigated by scratch method. After each well was covered with cells, a straight line was fabricated by the tip of a 20-μL pipette, and the cell debris was removed with PBS solution. The original medium was aspirated and a medium supplemented with various CTB nanozymes. The images of the L929 cells were captured with optical microscopy with the culture time of 24 h. The scratch widths at 0 and 24 h were measured using ImageJ software, and cell migration ratio was determined by the following equation: Wound closure (%) = A_a_/A_b_ × 100%, in which A_a_ is the scratched area at the beginning of time (0 h) and A_b_ is the healed scratch area at the end of time (24 h), respectively.

*Angiogenesis effect on CTB nanozymes:* The angiogenesis effect on CTB nanozymes was evaluated with tube formation assay.^[7]^ HUVECs were co-cultured with various concentrations of CTB nanozymes or PBS for 4 h, followed by 6 h culture in 96-well plates coated with Matrigel (50 μL, Corning, USA). An inverted microscope (Olympus, Japan) was used to capture images of three random fields. The quantification of the number of tube-like structures was evaluated by ImageJ software, such as the number of branch points and capillary length. Additionally, the levels of VEGF in culture supernatant was measured via enzyme-linked immunosorbent assay (ELISA) kit.

*Fabrication of* *MN@CT MN@CTB microneedle patches:* The MN@CT and MN@CTB microneedle patches were fabricated using uniform silicone molds (Hangzhou, China). Briefly, the LAP solution (0.5%) and HAMA aqueous solution (10%) were mixed as the MN solution. The MN solution (500 μL) supplied with CT or CTB nanozymes (200 μg/mL, 500 μL) as CT and CTB nanozymes solution, respectively. To prepare the MN@CT and MN@CTB microneedle patches, the CT or CTB nanozymes solution (150 μL) was injected into a PDMS mold. After vacuum de-bubbling and repeated drying, the MN@CT and MN@CTB microneedle patches were obtained by using UV light irradiation for 20 s. Next, PVA solution (20%) was added to the bottom of the PDMS model and subjected to the drying process.^[8]^ Lastly, MN@CT and MN@CTB microneedle patches were stripped from the PDMS model for further use.

*Characterizations of* *MN@CTB microneedle patches:* The morphology and chemical composition of MN@CTB microneedle was characterized by a scanning electron microscopy (SEM, FEI, Nova 400, USA) and an X-ray photoelectron spectrometer (XPS, ESCALAB250Xi, Thermo Scientific, USA), respectively. The photothermal effect and NO release profile, antibacterial and anti-oxidant capacities of MN@CTB were further evaluated as aforementioned.

*Cytocompatibility of* *MN@CTB microneedle patches:* Live/dead staining and CCK-8 assays were performed on mouse fibroblasts (L929) and human umbilical vein endothelial cells (HUVECs) to evaluate MN@CTB microneedle patch cytocompatibility. For live/dead staining, L929 or HUVECs (2.0 × 10^4^ cells/mL) were incubated with MN and MN@CTB microneedle patches at 37 ℃ for 2 days. FDA/PI solution (10 mM) was used to stained with cells under dark conditions for 10 minutes after the cells were washed with PBS twice. Images were acquired using a fluorescence microscope (Olympus, Japan). The specific step of the CCK-8 assay was described as aforementioned.

*HUVECs migration:* Cell migration was determined by a transwell assay to investigate the recruitment of HUVECs by the MN@CTB microneedle patches. Briefly, HUVECs (2.0 × 10^4^ cells/mL) were cultured in the upper chamber of a 24-well transwell plate (8 μm of pore size; Corning, China), and the underlying culture well was filled with MN and MN@CTB solutions (500 μL). After 24 h of incubation, HUVECs migrating from the transwell chamber to the bottom were fixed with 4% PFA and stained with crystal violet (0.2%). Lastly, the treated cells were viewed under an microscope (Olympus, Japan), and quantitative analysis of transmigrated HUVECs was performed in ImageJ software.

*Immunofluorescence staining**:* RAW264.7 cells were cultured with DMEM supplemented with lipopolysaccharide (LPS, 50 ng/mL) for 16 h, after which they were cultured with different samples. With the culture time of 24 h, cells were fixed with 4% PFA, rinsed with PBS, and then permeabilized with 0.2% Triton-X100. Then, the cells were incubated with anti-CD86 and anti-CD206 antibodies at 4 °C overnight. Afterward, the RAW264.7 were incubated with the primary antibody (CD86 and CD206) at 4 ℃ overnight, and fluorescently labeled secondary antibody (Red). Meanwhile, the skeleton and nuclei of RAW264.7 cells were incubated with FITC-phalloidin and Hoechst 33258. Lastly, the images were captured using a confocal laser scanning microscope (CLSM 800, Zeiss, Germany).

*Anti-inflammatory efficacy assessment-Luminex analysis*: The inflammatory cytokines and chemokines of culture medium were investigated by a Luminex protein biochip testing system. Briefly, the culture medium were harvested and homogenized in the lysate, then centrifuged and the supernatant was collected. The following cytokines in the supernatant were detected using the mouse cytokine antibody array (ab197465, Abcam) and Bio-Plex 200 system (Bio-Rad, Shanghai, China). The targets including GM-SCF, IFN-𝛾, IL -1𝛼, IL-1𝛽, IL-2, IL-3, IL-4, IL-5, IL-6, IL-9, IL-10, IL-12, IL-13, IL-17, KC, MCP-1, MCSF, RANTES, TNF-𝛼, and VEGF.

*Inflammatory factor detection:* The activated RAW264.7 cells were incubated with diverse samples for 2 days. After centrifuging each sample, the supernatants were collected, and the corresponding ELISA kits were used to measure the concentrations of interleukin-1β (IL-1β) and interleukin-10 (IL-10) following the manufacturer’s instructions.

*Angiogenesis properties of MN@CTB microneedle patches:* The angiogenic markers of CD31 and VEGF were evaluated with immunofluorescence staining, qRT-PCR analysis, ELISA, western blot (WB) analysis, and respectively. The particular steps of the immunofluorescence staining and ELISA were described as aforementioned. For quantitative real-time polymerase chain reaction (qRT-PCR) assays, the HUVECs (5.0 × 10^3^ cells/mL) were cultured into a 6-well plates and incubated with various samples for 2 days. Then, the total RNA of HUVECs was extracted using a Total RNA Extract reagent (Takara, Japan), and the concentration of total RNA was quantified by a NanoDrop One reader (Thermo Scientific, USA), respectively. To reverse transcribe the RNA (500 ng) into cDNA, a PrimeScript RT Master Mix reagent kit was used (Takara, Japan). The qRT-PCR assay utilized a 10 µL reaction system, which contained cDNA and TB Green^TM^ Premix Ex Taq^TM^ (Takara, Japan) along with forward and reverse primers. Lastly, the relative expression angiogenesis-related markers were determined by the primers (Sangon, China) in Table S1 (Supporting Information). The GAPDH was used as the housekeeping gene.

For the WB analysis, the treated cells were rinsed with PBS twice, lysed with RIPA lysis buffer in an ice bath, and then the supernatant was collected by centrifugation. The protein concentration in the supernatant was measured, and then the obtained proteins were mixed with loading buffer and denatured. The collected proteins were separated through sodium dodecyl sulfate–polyacrylamide gel electrophoresis (SDS-PAGE) and transferred to the polyvinylidene difluoride membranes (PVDF) membrane. Afterward, the membranes were blocked with skim milk (5%), immersed in TBST solution for 2 h, and incubated with primary antibodies (anti-GAPDH, anti-VEGF, and anti-CD31) at 4 ℃ overnight. The membranes were treated with secondary antibodies after rinsing again with TBST solution. Bolts were measured using enhanced chemiluminescence reagents and imaged by the Fusion imaging system (Ktimes, Germany). Lastly, the protein expression levels of CD31 and VEGF were normalized to GAPDH using ImageJ software.

*In vivo wound healing assays of the MN@CTB* *microneedle patches:* All animals were performed by the Animal Ethics Committees of Nanchang University (Approval No. CDYFY-IACUC-202404QR016). The female Balbc/c mice (20-25 g) were employed to build an MRSA-infected wound model.^[9]^ Briefly, the mice were anesthetized by injection of pentobarbital sodium solution (w/v, 3%), and then the fur of the backside was shaved and disinfected. Next, the full-thickness skin round wounds (approximately 5 mm) were constructed and then infected by MRSA suspension (100 μL, 1.0 × 10^9^) on the back of mice. Afterward, all the infected wounds were fully covered with a special kind of plastic sticker (Tegaderm Film, 3M^TM^, 3662 CU). After 24 h, the twenty-four mice were randomly divided into four groups: control, MN, MN@CTB, and MN@CTB + NIR. These microneedle patches were inserted into MRSA-infected wounds and remained on the wounds for 1 day. All wounds were treated with MN@CTB microneedle patches for 3 days. The images of the wound sites were captured on day 0, 3, 7, and 14 in each group, and the wound contraction (%) was analyzed using ImageJ software. For *in vivo* antibacterial evaluation, the wounds were collected and homogenized with LB broth (2 mL) under aseptic conditions on day 3. With the culture time of 24 h, the diluted suspension (10000×) was smeared onto LB agar plates. The CFUs of each group were captured and the antibacterial capacities were investigated as aforementioned. Besides, the levels of inflammation and angiogenesis-associated cytokines (TNF-α, IL-10, VEGF, and CD31) were measured with ELISA as aforementioned.

*Histological analysis:* For histological analysis, the skin tissue surrounding the wound site was harvested, fixed in 4% formaldehyde, and embedded in paraffin on days 3, 7, and 14. Next, the treated tissues were cross-sectioned into slices (thickness: 5 μm) and the obtained slices were installed on slides. Lastly, the slides were treated with Giemsa, Hematoxylin-Eosin (H&E), Masson’s trichrome, and immunohistochemistry (IHC) staining kits according to the manufacturer’s instructions, respectively. Lastly, On day 7, the laser Doppler-scanned blood flow imager (RWD RFLSI system, Shenzhen, China) was employed to visualize the blood flow of the flap in real-time.

*Proteomic analysis*: The wound tissues of the control and MN@CTB + NIR groups were collected on day 3 postoperatively (n = 3), and protein quantification was performed using the Bradford method (Thermo Fisher Scientific, USA), followed by standard processing.^[10]^ Briefly, samples were mixed with trypsin to collect the peptides. The collected peptides were reconstituted with ammonium solution, subjected to elution, chromatographic separation, and mass spectrometry analysis using the proteomics analysis system (Thermo Fisher Scientific, USA) at Shanghai Majorbio Biotechnology Co., Ltd. Proteomic data were analyzed using MaxQuant software and compared with the UniProt database. The significant differentially expressed proteins were screened using a consistent standard (fold change ≥ 1.5 and P value < 0.05), and the Gene Ontology (GO) and Kyoto encyclopedia of genes and genomes (KEGG) pathway analysis enrichment were employed for further bioinformatics analyses.

*Allergy scores*: Allergy scores were conducted according to the previous approach with minor modifications. After being treated with MN@CTB microneedle patches, allergy scores were observed within 30-60 min (Table S2, Supporting Information). The body surface temperature was recorded using an infrared thermal imager.

*Detection of serum indicators:* The expression levels of IgE and HIS related to allergic reaction in the mice serum was investigated according to the manufacturer's instructions (Jiang Lai, Shanghai, China).

*Evaluation of Cu^2+^ distribution in the main organs and blood in vivo:* After the wounds of the MRSA-infected mice was treated with MN@CTB microneedle patches for 6, 24, and 48 h, respectively. The main organs (heart, liver, Spleen, lung, and kidney) and blood was collected to investigate the Cu^2+^-distribution after administration of MN@CTB microneedle patches.

*Hemolysis analysis of MN@CTB microneedle patches:* To investigate erythrocyte hemolysis in the control, MN@CTB, Triton-X100 groups. The erythrocyte suspensions was prepared by utilizing sterile PBS to dilute the blood specimens. Subsequently, MN@CTB microneedle patches were mixed with 500 μL of erythrocyte suspension as test samples, with PBS and Triton-X100 used as negative and positive control groups, respectively. After incubation at 37°C for 120 min, the hemolysis rate (%) was calculated as (A_m_ - A_p_)/(A_t_ - A_p_) × 100% through centrifugation (10,000 rpm, 10 min) and measuring the supernatant’s absorbance at 540 nm. A_m_ indicates the supernatant uptake OD value of the MN@CTB group, A_p_ indicates the negative control group, and A_t_ indicates the positive control group.

*Blood analysis:* The mice were divided into two groups (control and MN@CTB), which was treated with PBS or MN@CTB microneedle patches respectively. Blood was taken from the ocular vein for analysis after 14-day treatment.

*Statistical analysis:* The results were presented as mean ± standard deviation (SD) from indicated independent replicates (n ≥ 3). The statistical significance was conducted using Origin software (version 8.5) *via* one-way analysis of variance (ANOVA) and post-hoc Tukey test. The statistical significance was considered by the values of *p** < 0.05 and *p*** < 0.01, respectively.

**References**:

[1] G.H. Liu, L.C. Wang, J.J. Liu, L. Lu, D. Mo, K. Li, X. Yang, R. Zeng, J.X. Zhang, P. Liu, K.Y. Cai, *Adv. Healthc. Mater*. **2020**, 9, 2000432.

[2] a) Y.L. Yu, J.J. Wu, C.C. Lin, X. Qin, F.R. Tay, L. Miao, B.L. Tao, Y. Jiao, *Mil. Med. Res*. **2023**, 10, 21; b) Z. Yuan, C.C. Lin, Y. He, B.L. Tao, M.W. Chen, J.X. Zhang, P. Liu, K.Y. Cai, *ACS Nano* **2020**, 14, 3546.

[3] B.L. Tao, W.W. Yi, X. Qin, J.J. Wu, K. Li, A. Guo, J. Hao, L.X. Chen, *J. Mater. Sci. Technol*. **2023**, 146, 131.

[4] Z. Yuan, C.C. Lin, L.L. Dai, Y. He, J.W. Hu, K. Xu, B.L. Tao, P. Liu, K.Y. Cai, *Small* **2021**, 17, 2007522.

[5] S.B. Li, X.M. Wang, Z.Y. Yan, T. Wang, Z.B. Chen, H. Song, Y.B. Zheng, *Adv. Sci*. **2023**, 10, 2300576.

[6] C. Shi, Y. Zhang, G.F. Wu, Z.Y. Zhou, H.P. Zheng, X.M. Sun, Y.Y. Heng, S.W. Pan, H.N. Xiu, J. Zhang, Z.W. Yin, Z.Y. Yu, B. Liang, *Adv. Healthc. Mater.* **2024**, 13, 2302626.

[7] Y.L. Jin, Y. Lu, X. Jiang, M. Wang, Y.N. Zeng, J. Guo, W. Li, *Bioact. Mater.* **2024**, 38, 292-304.

[8] S. Guo, W.L. Zhang, X.X. Zhai, X. Zhao, J.X. Wang, J. Weng, J.S. Li, X.Y. Chen, *Biomater. Sci.* **2023**, 11, 533.

[9] Y.Q. Liang, Z.L. Li, Y. Huang, R. Yu, B.L. Guo, ACS Nano **2021**, 15, 7078.

[10] a) J.Y. Qian, E.H. Lu, H.B. Xiang, P.B. Ding, Z. Wang, Z.Y. Lin, B.L. Pan, C. Zhang, Z.M. Zhao, J. Nanobiotechnol. 2024, 22, 550; b) M.L. Liu, R. Ding, Z. Li, N. Xu, Y.L. Gong, Y. Huang, J.Z. Jia, H.Y. Du, Y.L. Yu, G.X. Luo, Adv. Sci. 2024, 11, 2306602.

**Table S1.** Real-time PCR primers of HUVECs used in the present study

| **Target gene** | **Primers sequences** |
| --- | --- |
| CD31 | F: 5’- ACCGTGACGGAATCCTTCTCT-3’  R: 5’-GCTGGACTCCACTTTGCAC-3’ |
| VEGF | F: 5’-AGCCCATGAAGTGGTGAAGT-3’  R: 5’-GCTCACAGTGATTTTCTGGCTT-3’ |
| GAPDH | F: 5’-TGTCCCTAATGACAGCTCCTT-3’  R: 5’-GCATCCACCCAAATGACACA-3’ |

**Table S2.** The scores of allergy

| **Score** | Allergic symptom |
| --- | --- |
| 0  1  2 | no symptoms  cratching and rubbing around the nose and head  puffiness around the eyes and mouth; reduced activity, and/or decreased activity |
| 3  4  5 | static for more than 2 min; hair shaft; increased respiratory rate  no activity after prodding or tremor and convulsion  death |

**

**

**Figure S1.** The standard curve of BNN6.

**
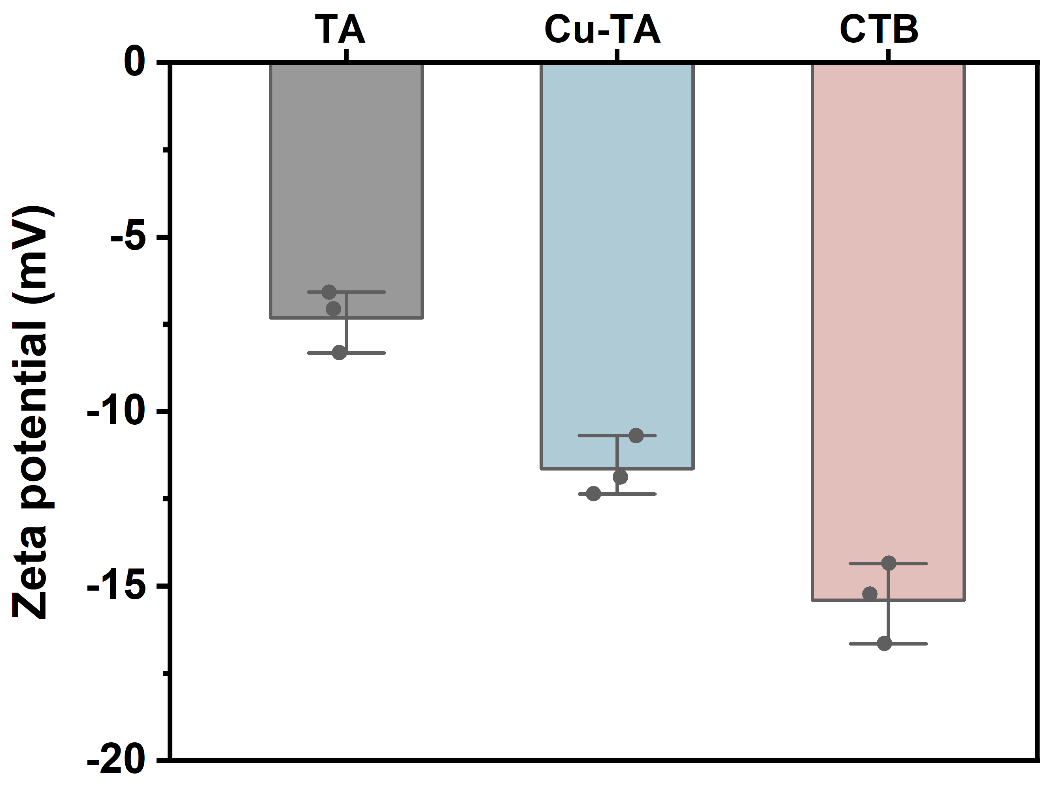
**

**Figure S2.** Zeta potential of TA, Cu-TA, and CTB (n = 3).





**Figure S3.** Particle size of Cu-TA and CTB as determined by dynamic light scattering.

**
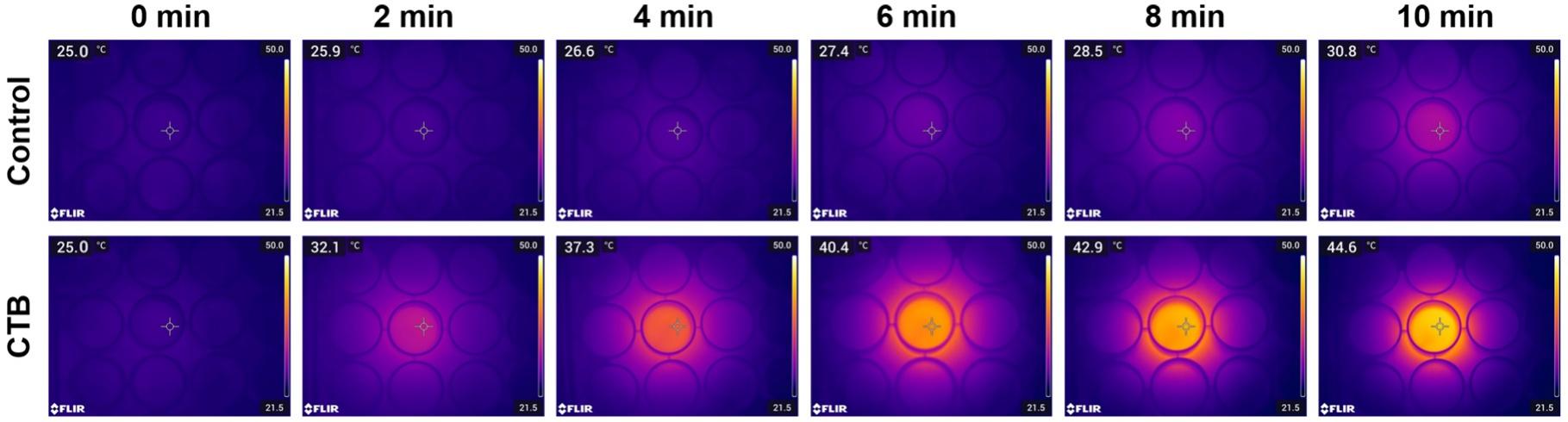
**

**Figure S4.** Infrared thermal images of CTB (100 µg/mL) under 808 nm laser with a power density of 1.0 W/cm at different times.

**

**

**Figure S5**. Heating and cooling profiles of CTB suspension (0 and 100 µg/mL) exposed to laser irradiation (808 nm, 1 W/cm^2^).

**

**

**Figure S6.** The cooling time plot versus negative natural logarithm of driving force temperature (-lnθ) with τ_s_ = 274.68 of the concentration of 100 µg/mL.

**

**

**Figure S7.** NO release from different concentration of CTB upon NIR irradiation (1.0 W/cm^2^) (n = 3).

**
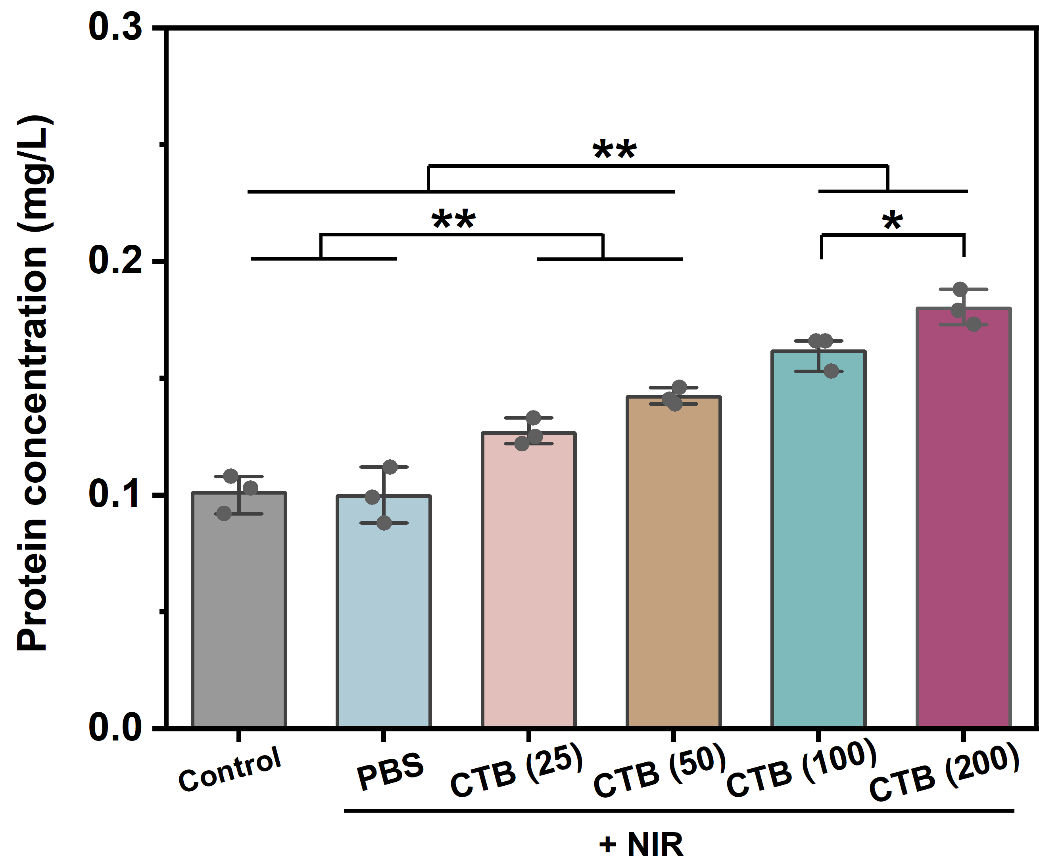
**

**Figure S8.** Leakages of protein from bacteria after incubation for 12 h (n = 3), **p* < 0.05, ***p* < 0.01.


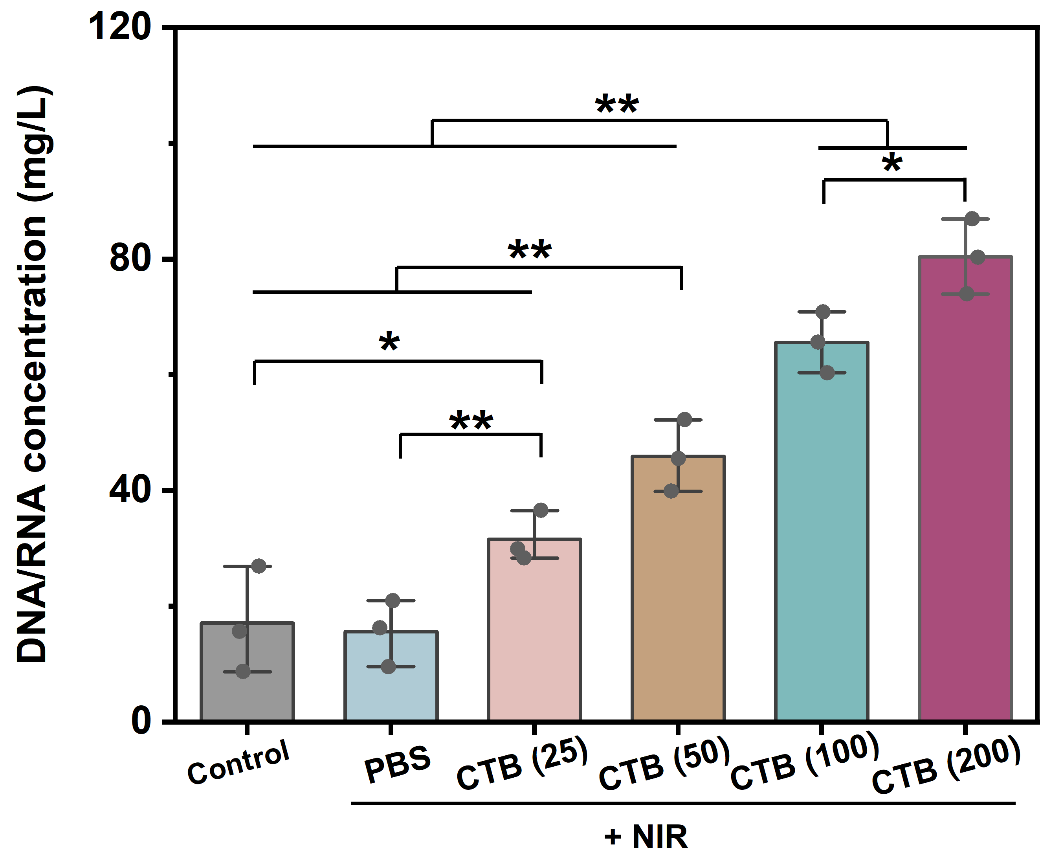


**Figure S9.** Leakage of DNA and RNA from MRSA after incubation for 12 h (n = 3), **p* < 0.05, ***p* < 0.01.

**
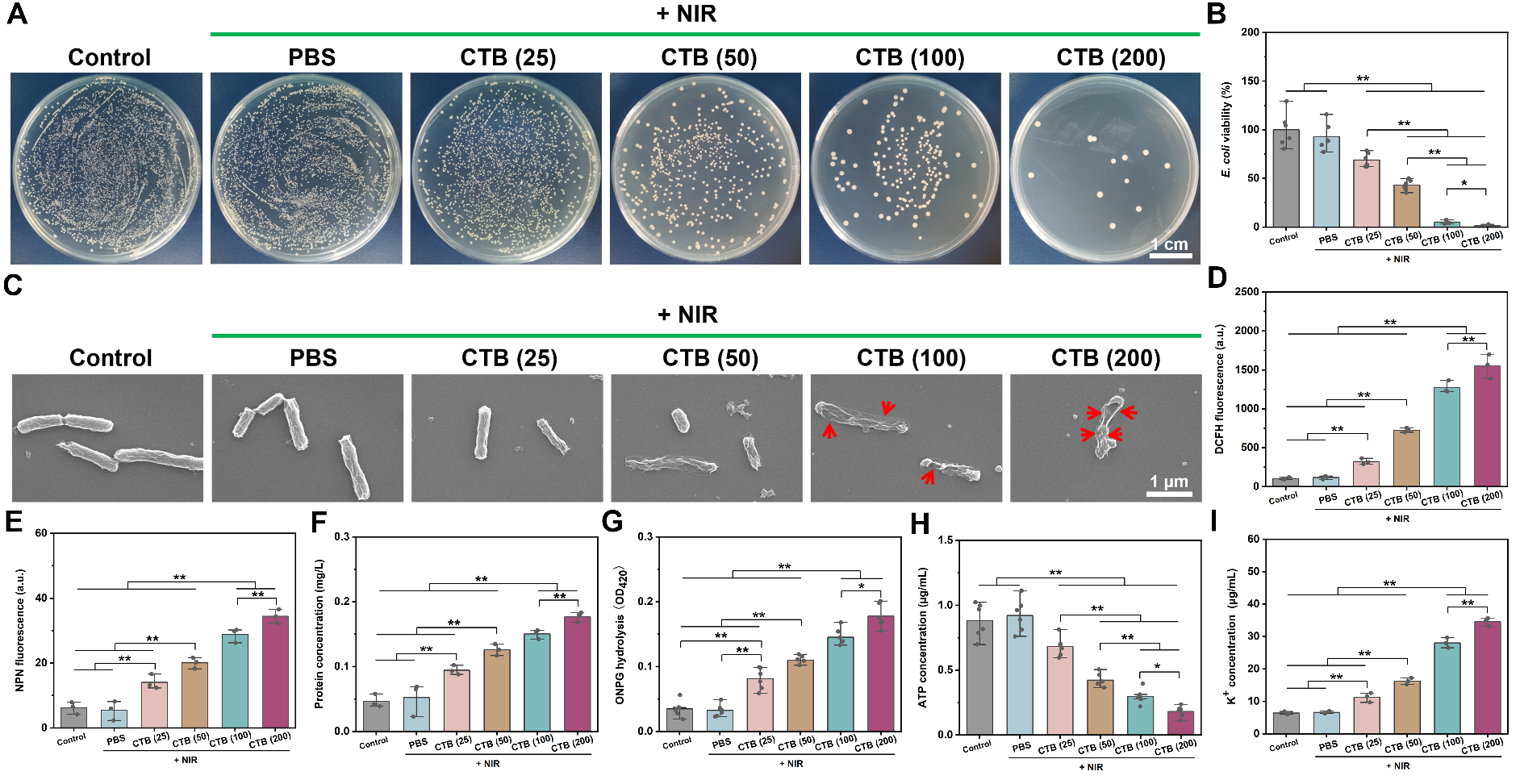
Figure S10**. The antibacterial capacity of various concentrations of CTB nanozymes. (A) Representative images of bacterial colonies formed by *E. coli* after different treatments. (B) The corresponding statistical analysis of the *E. coli* viability (n = 6). (C) SEM images of *E. coli* after different treatments, the red arrows indicate the damaged morphology of *E. coli*. (D) ROS intensity of *E. coli* after incubation with various samples (n = 3). (E) NPN fluorescence intensity (n = 3), (F) Leakage of protein (n = 3), (G) ONPG hydrolysis (n = 6), (H) ATP concentration (n = 6), and (I) extracellular released K^+^ concentration of *E. coli* after incubation with various samples (n = 3), **p* < 0.05, ***p* < 0.01.

**
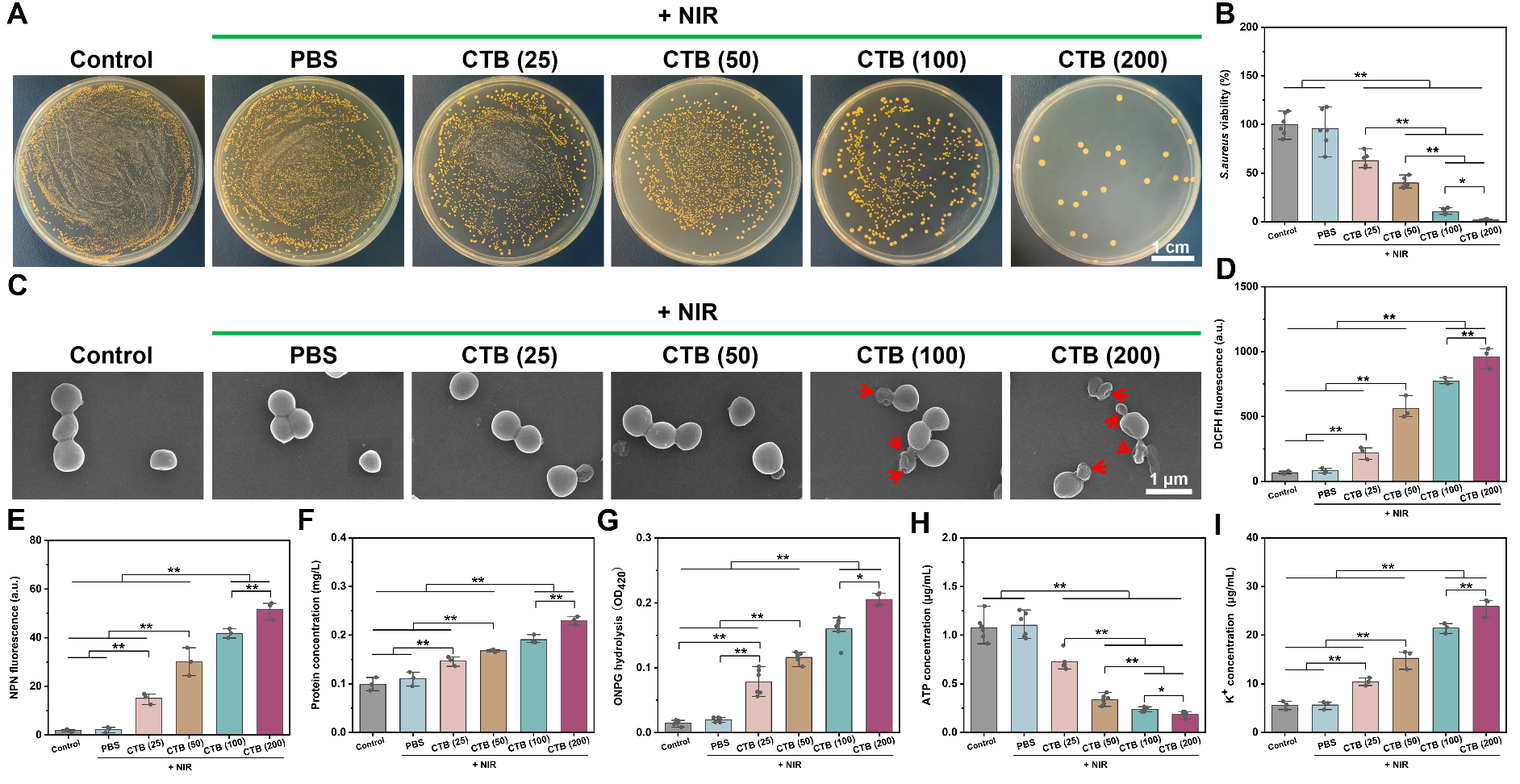
Figure S11**. The antibacterial capacity of various concentrations of CTB nanozymes. (A) Representative images of bacterial colonies formed by *S. aureus* after different treatments. (B) The corresponding statistical analysis of the *S. aureus* viability (n = 6). (C) SEM images of *S. aureus* after different treatments, the red arrows indicate the damaged morphology of *S. aureus*. (D) ROS intensity of *S. aureus* after incubation with various samples (n = 3). (E) NPN fluorescence intensity (n = 3), (F) Leakage of protein (n = 3), (G) ONPG hydrolysis (n = 6), (H) ATP concentration (n = 6), and (I) extracellular released K^+^ concentration of *S. aureus* after incubation with various samples (n = 3), **p* < 0.05, ***p* < 0.01.


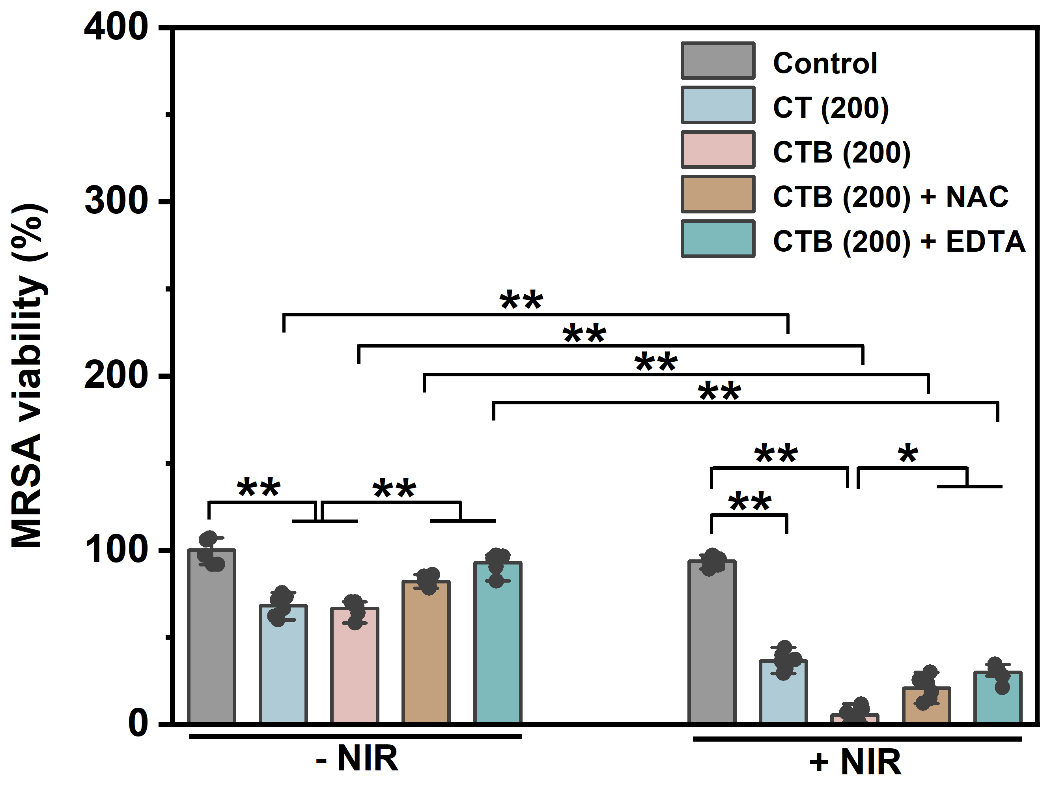


**Figure S12.** Statistical analysis of the relative viability of MRSA after incubation with various samples (n = 6), **p* < 0.05, ***p* < 0.01.


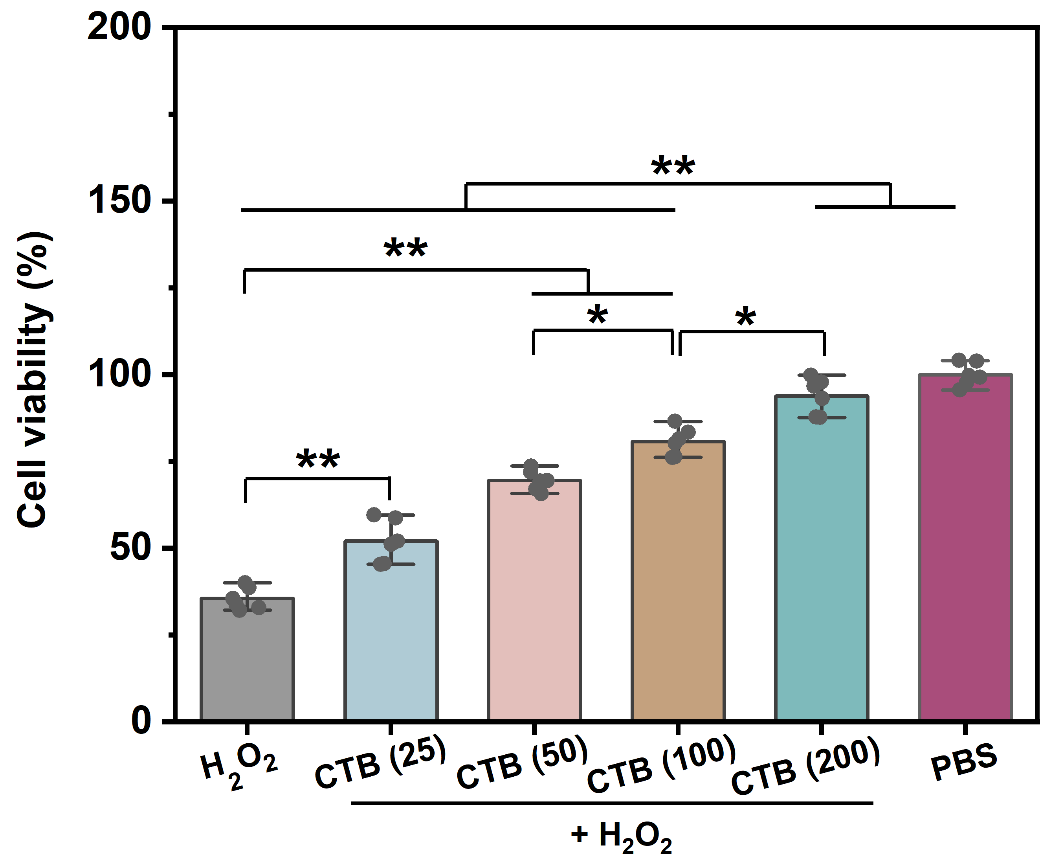


**Figure S13**. Relative cellular viability of L929 cells after 48 h of coincubation with various concentrations of CTB nanozymes and 0.1 mM H_2_O_2_ (n = 6) , **p* < 0.05, ***p* < 0.01.


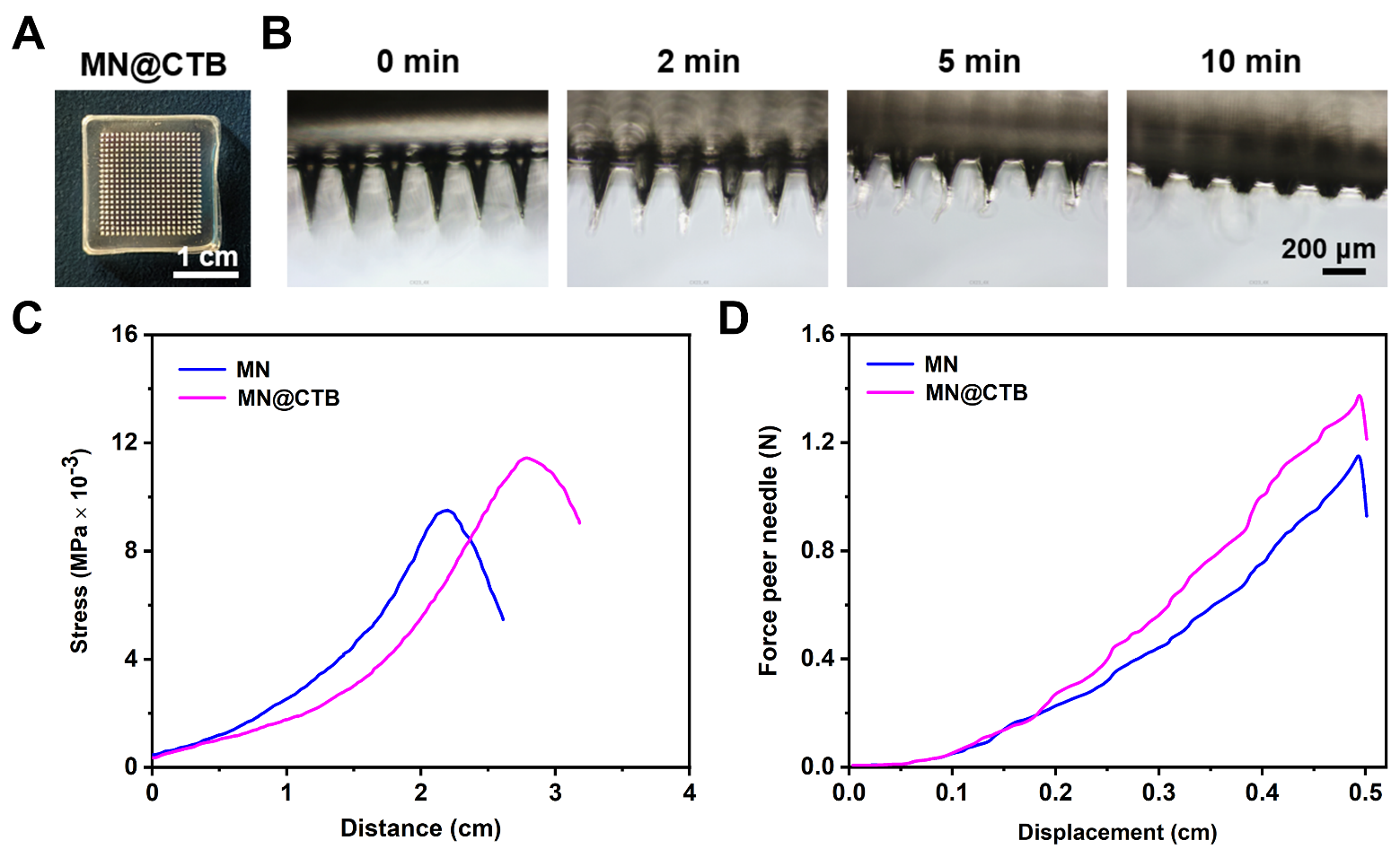


**Figure S14.** Characterization of MN@CTB microneedle patches. (A) Photographic image of MN@CTB microneedles patches. (B) *In vitro* dissolution image of MN@CTB microneedles patches. (C-D) The adhesion strength and mechanical strength of the MN@CTB microneedles patches.


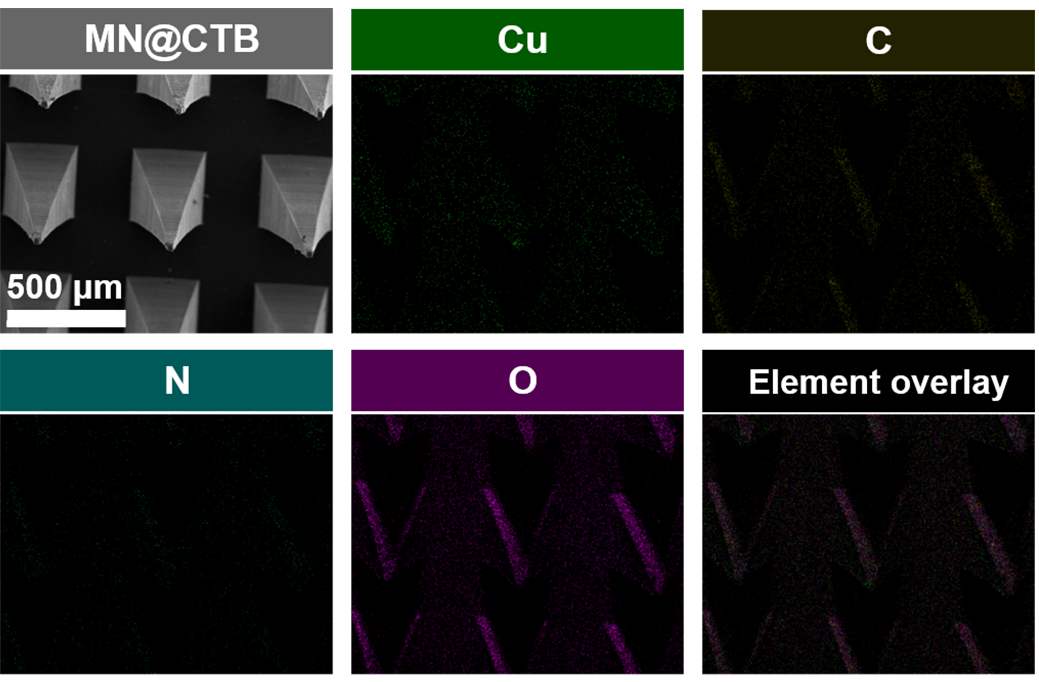


**Figure S15.** Elemental mapping of Cu, C, N, and O in MN@CTB.


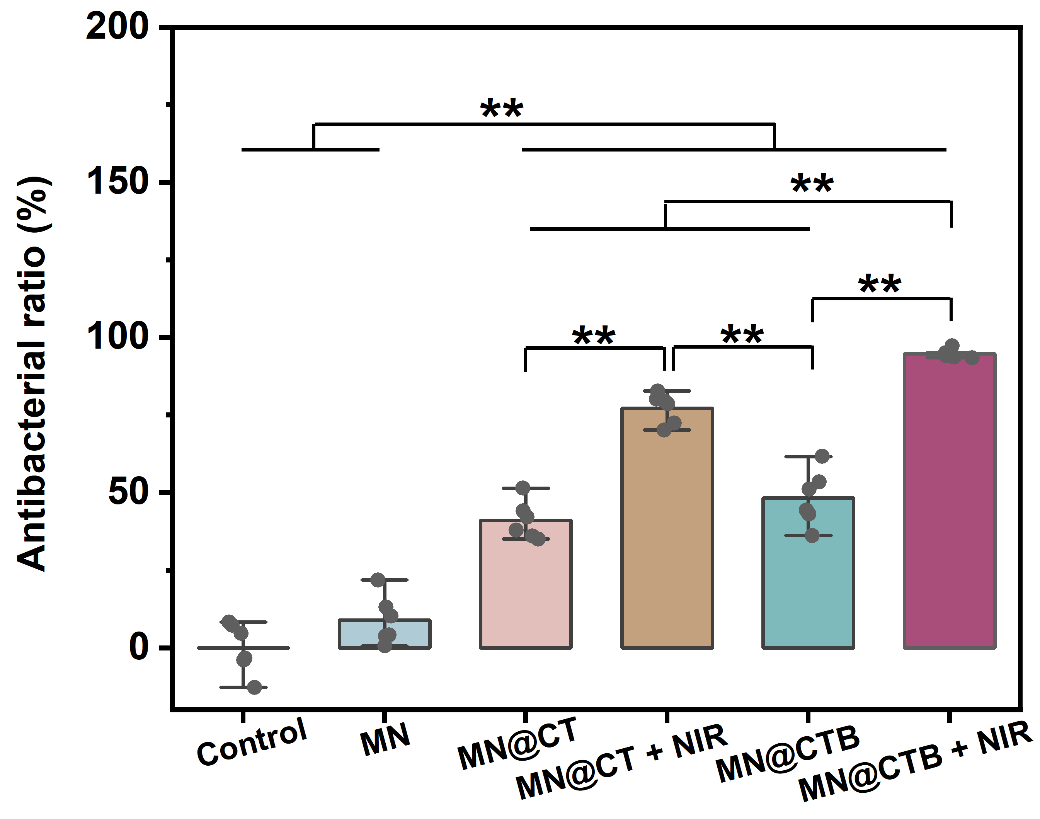


**Figure S16**. Antibacterial effect against MRSA with different treatments (n = 6), **p* < 0.05, ***p* < 0.01.


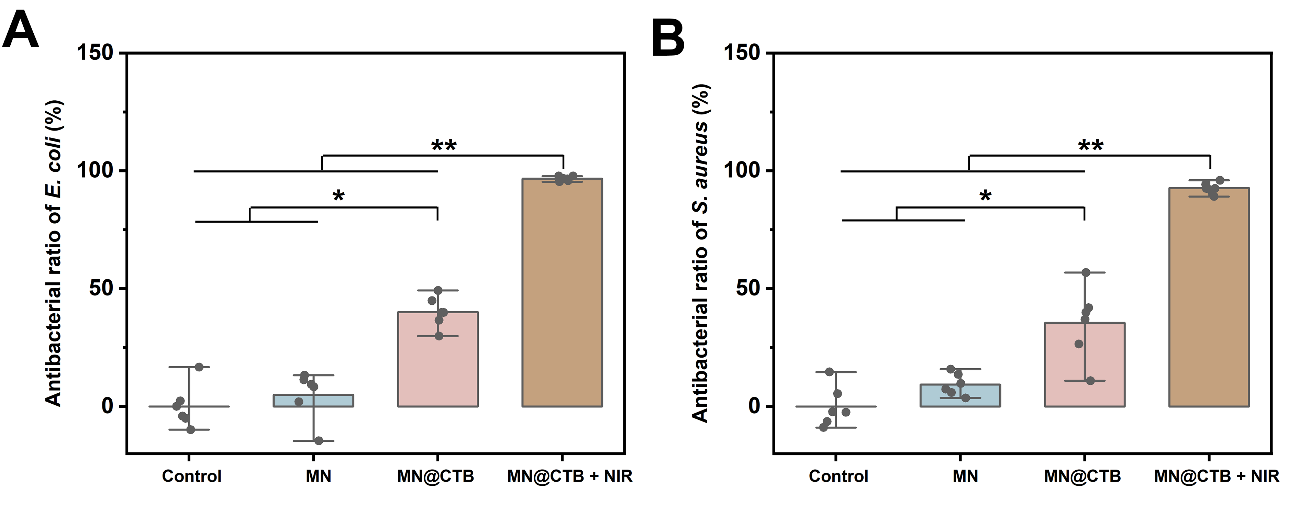


**Figure S17.** Antibacterial effect of MN@CTB microneedle patches against (A) *E. coli* (n = 6) and (B) *S. aureus* (n = 6), ***p* < 0.01.


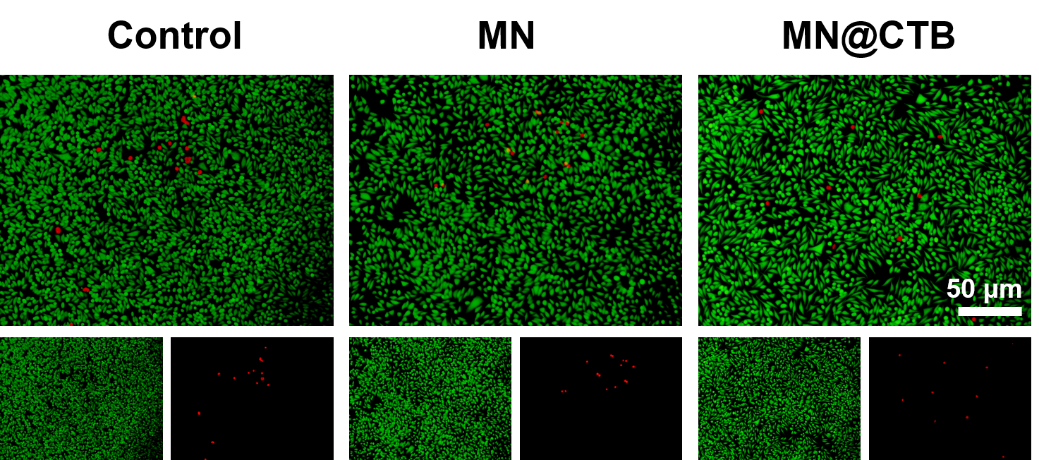


**Figure S18.** Live/dead staining of the L929 cells after treatment with MN and MN@CTB microneedle patches for 48 h.


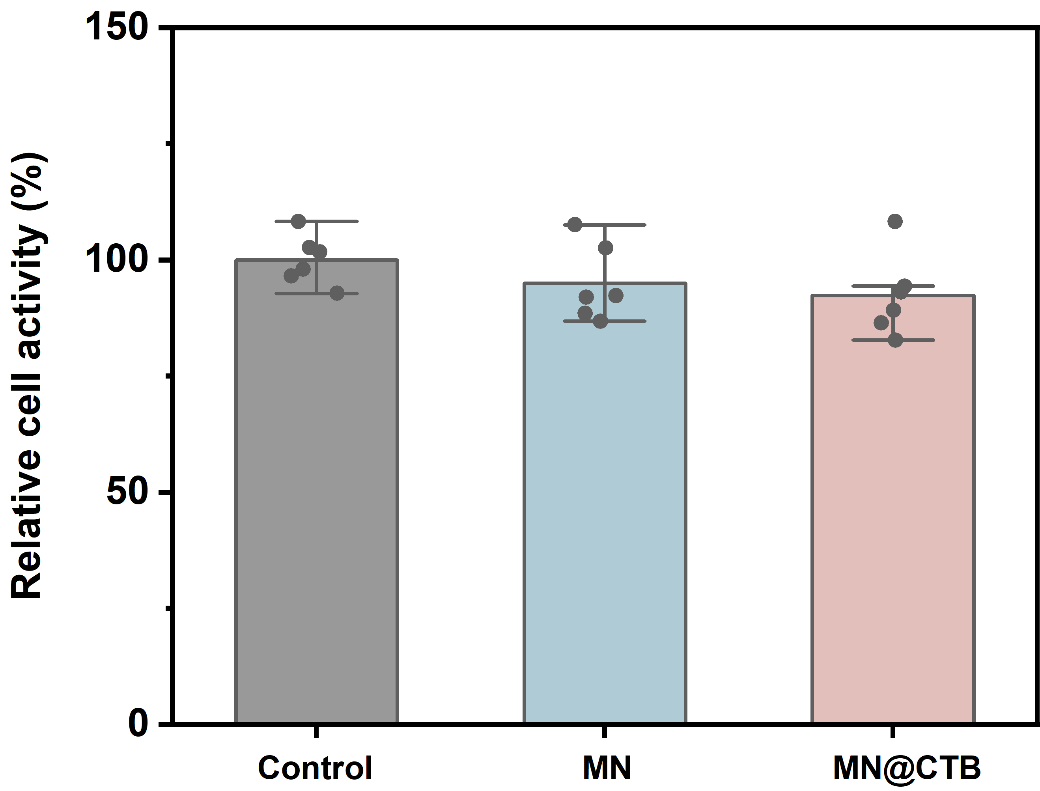


**Figure S19.** CCK-8 assay of L929 cells in each group (n = 6).


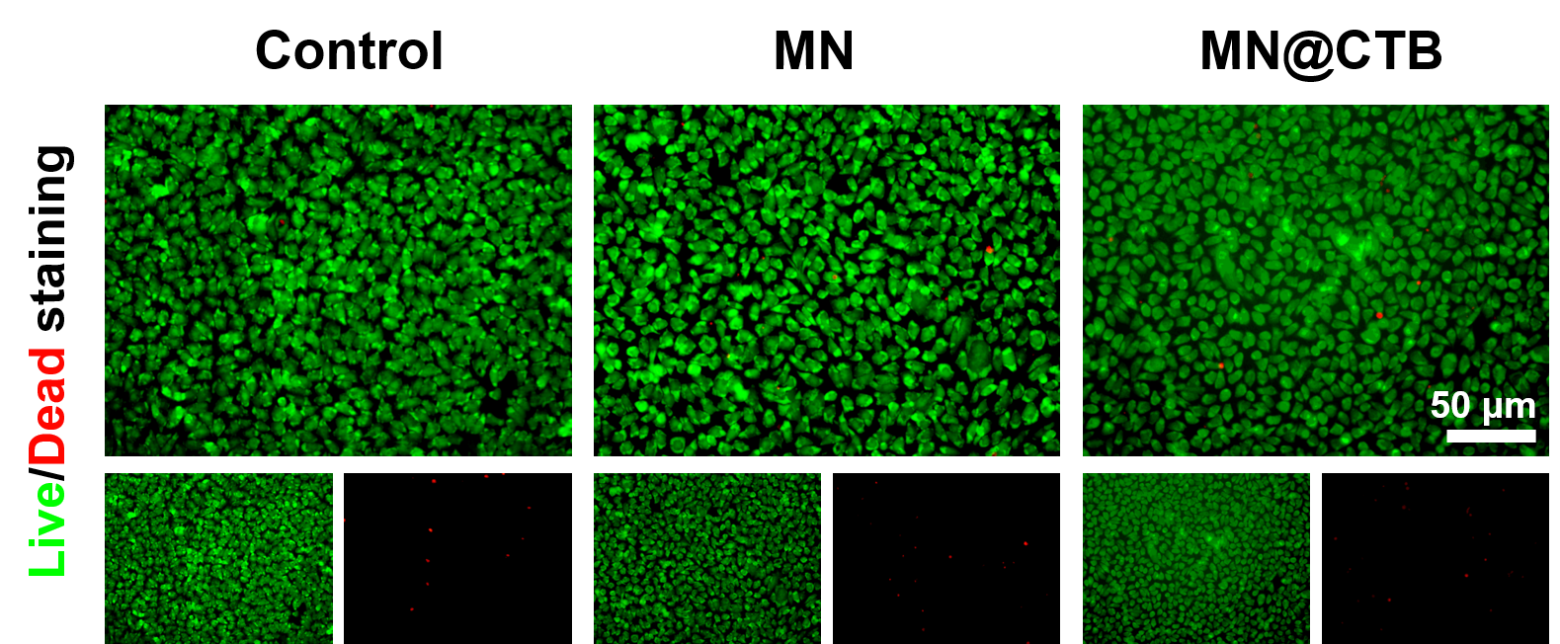


**Figure S20.** Live/dead staining of the HUVECs after treatment with MN and MN@CTB microneedle patches for 48 h.

**
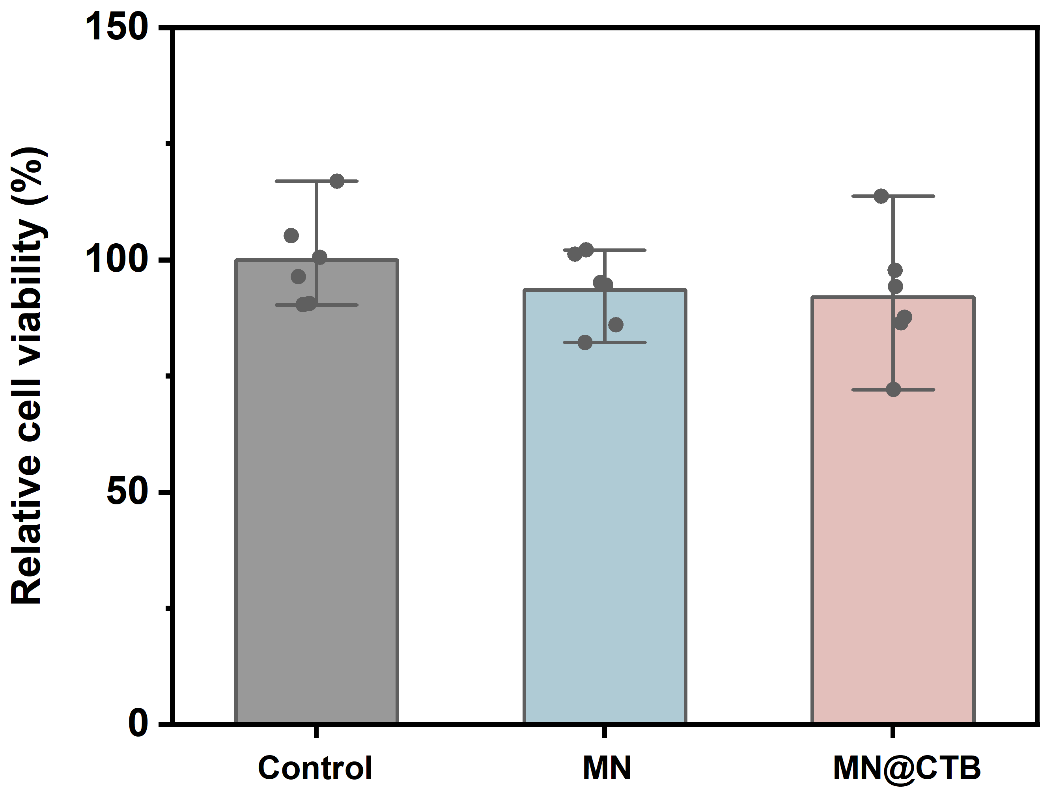
**

**Figure S21.** CCK-8 assay of HUVECs in each group (n = 6).


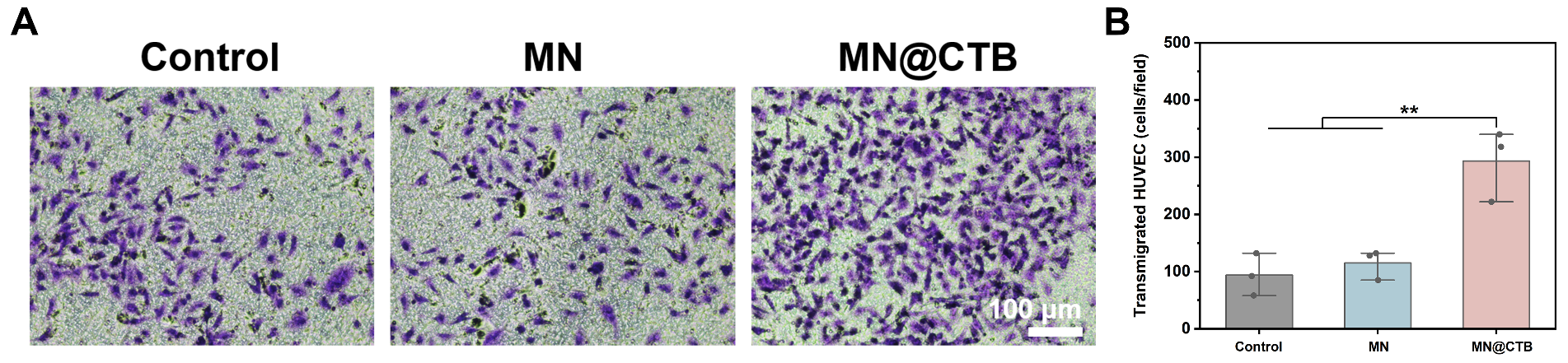


**Figure S22.** Cell migration capacities of MN@CTB microneedle patches *in vitro*. (A) HUVECs migration of MN and MN@CTB by transwell co-culture system for 24 h. (B) Statistical analysis of transmigrated HUVECs (n = 3), **p* < 0.05, ***p* < 0.01.


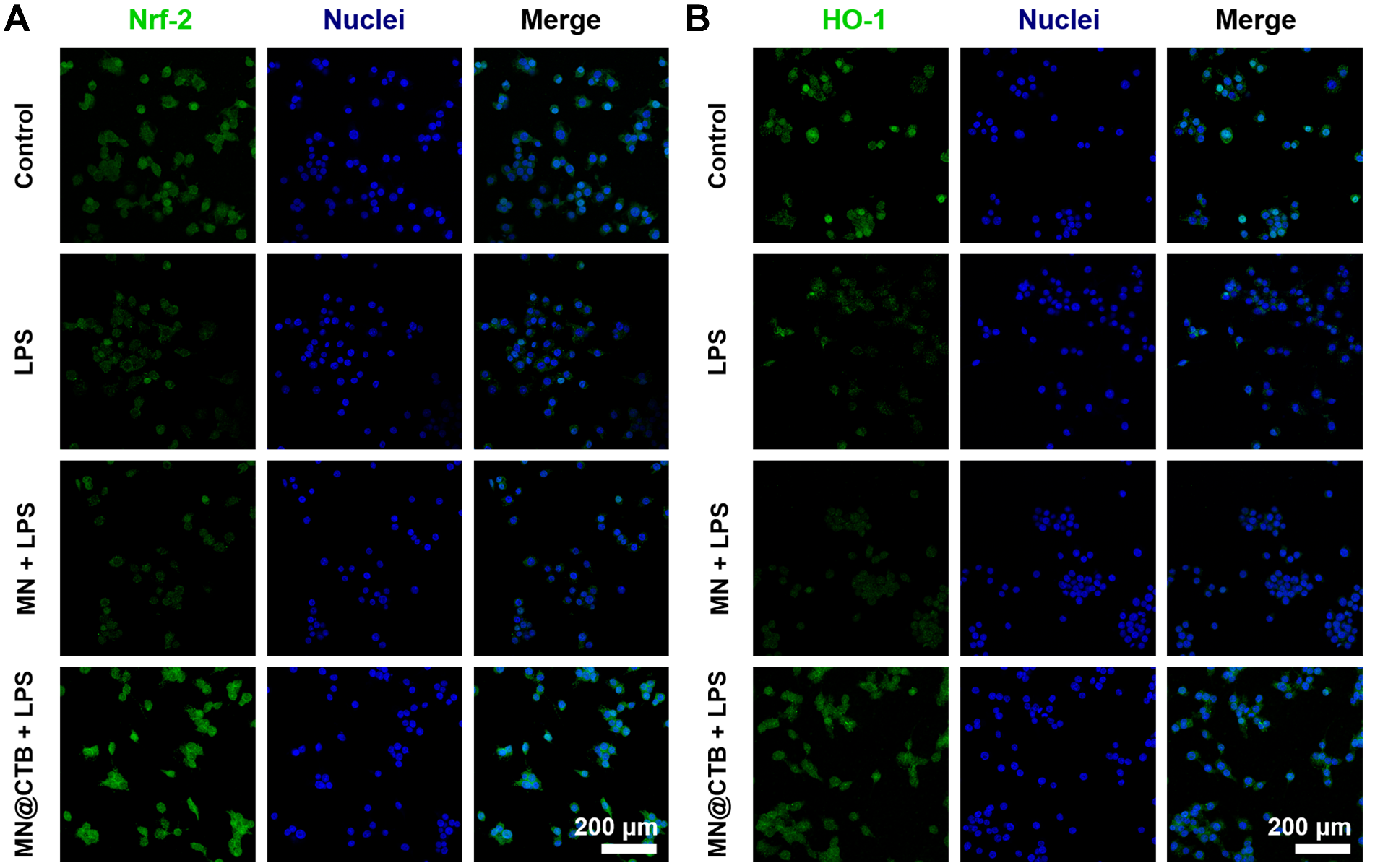


**Figure S23.** Antioxidant mechanisms of MN@CTB microneedle patches. (A-B) The immunofluorescence images of Nrf-2 and HO-1 in RAW 264.7 cells on different samples.


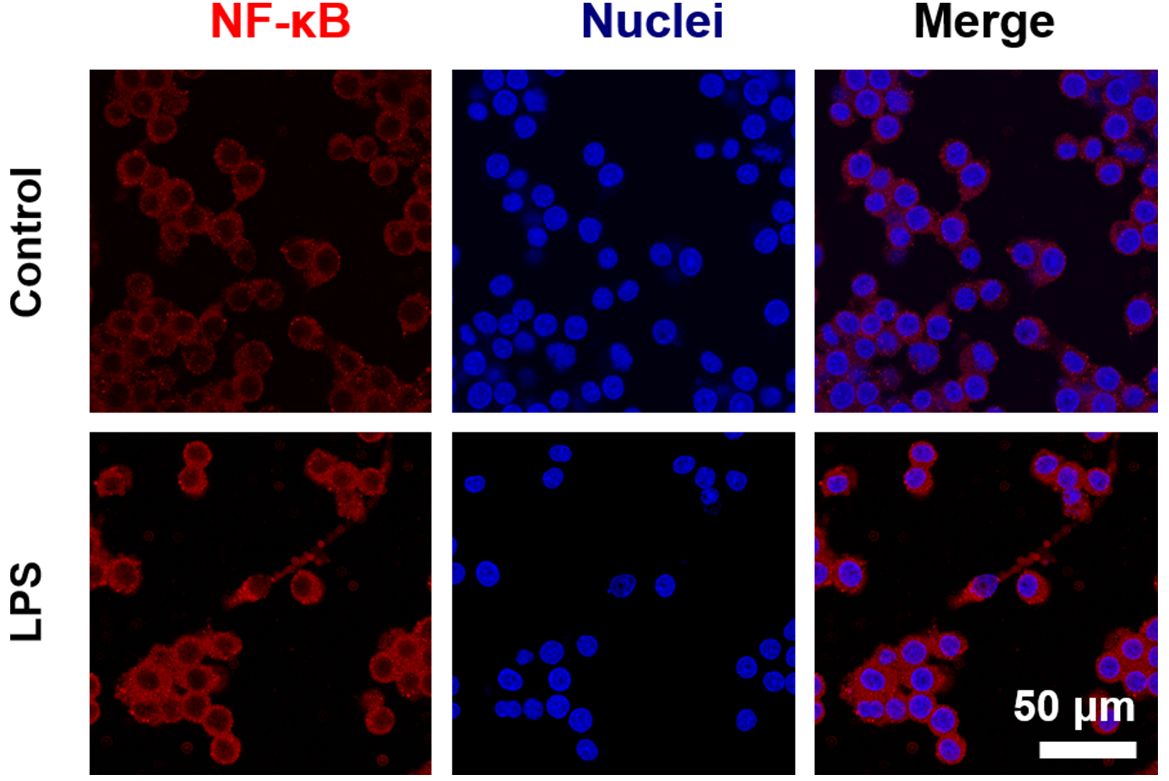


**Figure S24**. The nuclear translocation of NF-κB after treated with LPS within a short time (60 min).


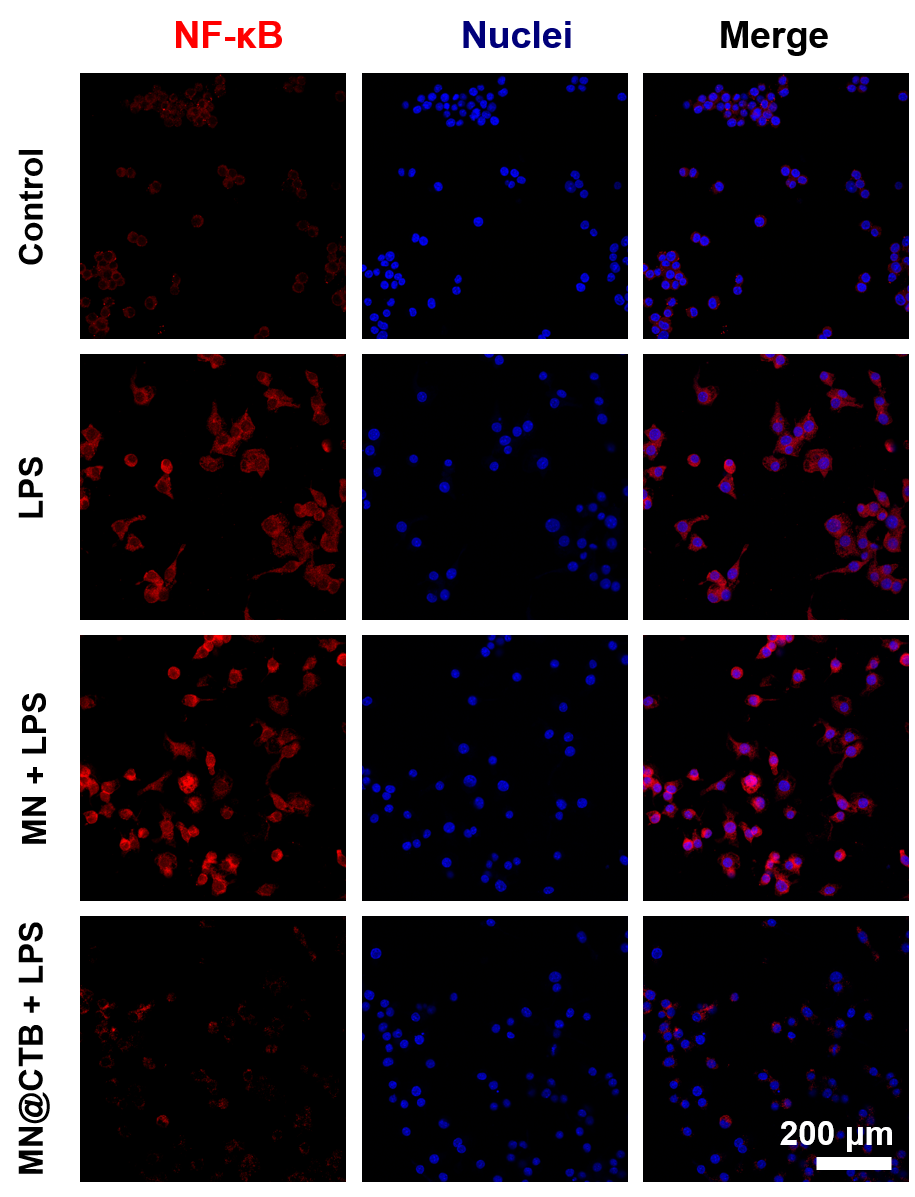


**Figure S25.** The immunofluorescence images of NF-κB in RAW 264.7 cells on different samples.


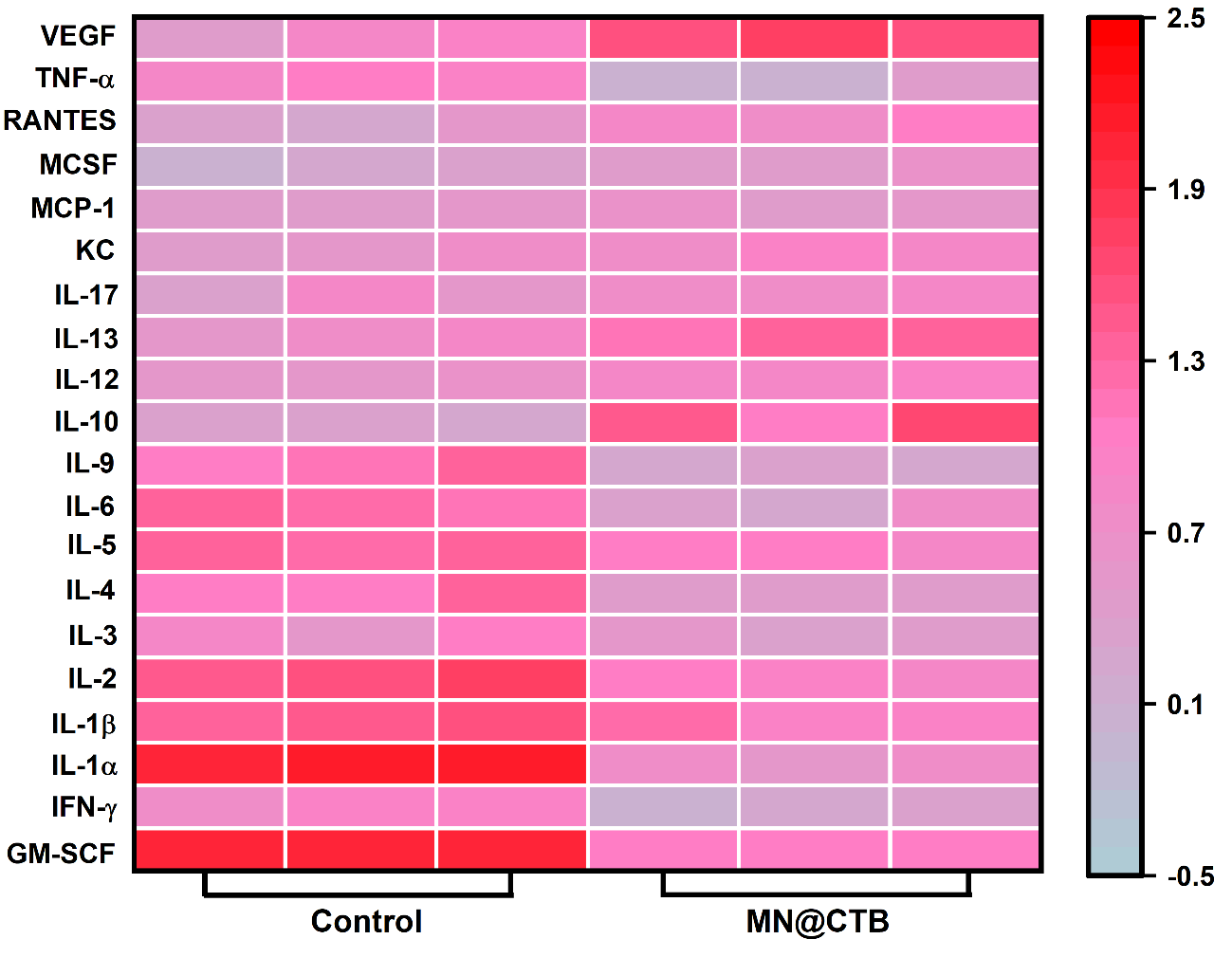


**Figure S26**. Protein expression levels of 20 cytokines and chemokines in culture medium were detected by Luminex (n = 3).


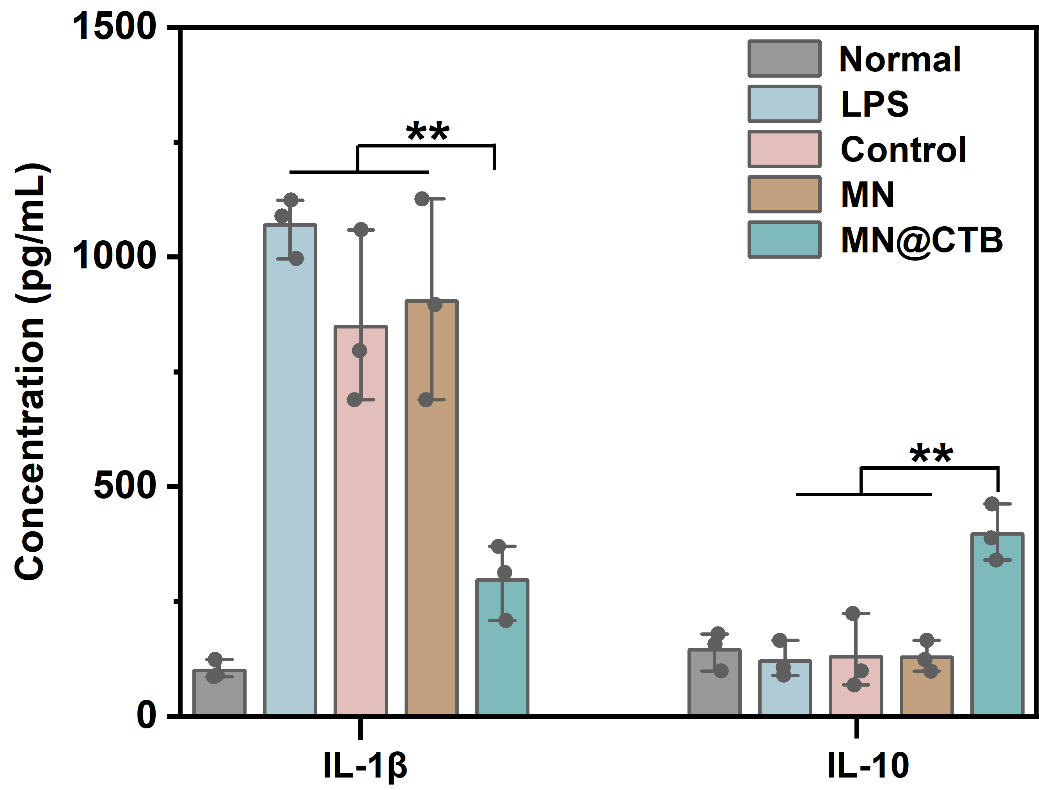


**Figure S27**. Statistical analysis of ELISA results of IL-1β and IL-10 (n = 3).


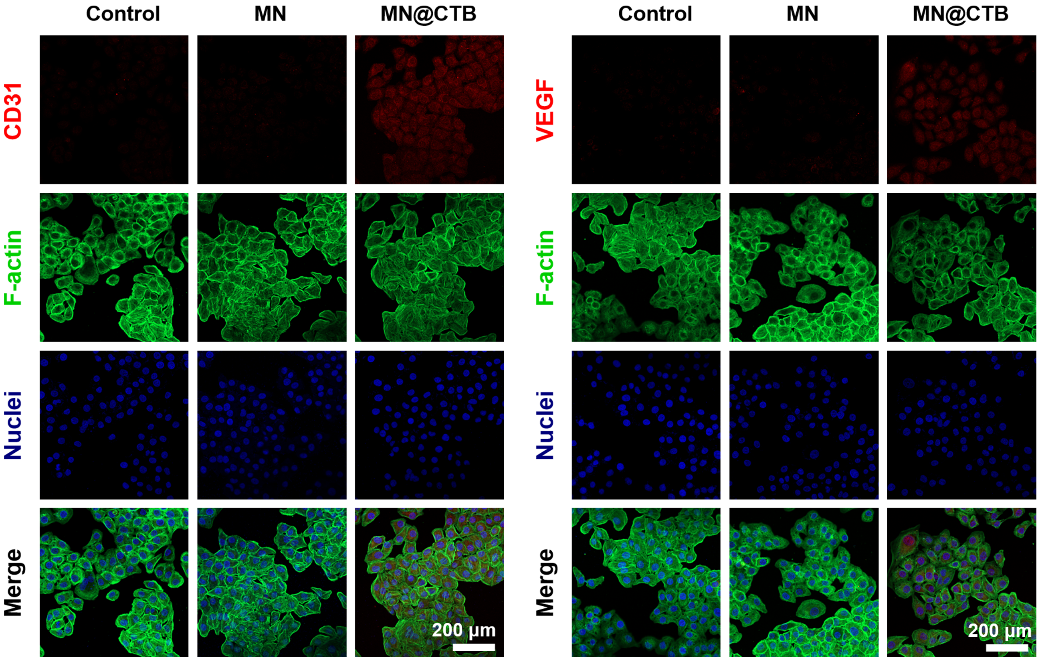


**Figure S28.** CD31 and VEGF immunofluorescence staining of HUVECs on different samples.

**
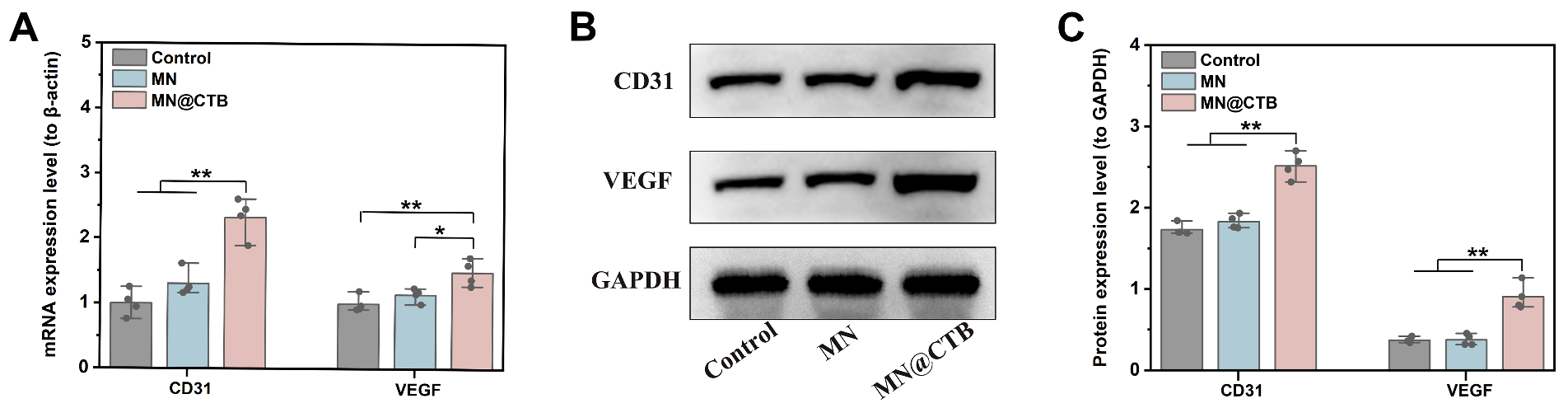
**

**Figure S29.** Angiogenic capacities of MN@CTB microneedle patches *in vitro*. (A) mRNA levels of angiogenesis-related genes CD31 and VEGF for 2 days (n = 4). (B-C) The protein expressions of CD31 and VEGF in each group were investigated by western blot (n = 4), **p* < 0.05, ***p* < 0.01.

**
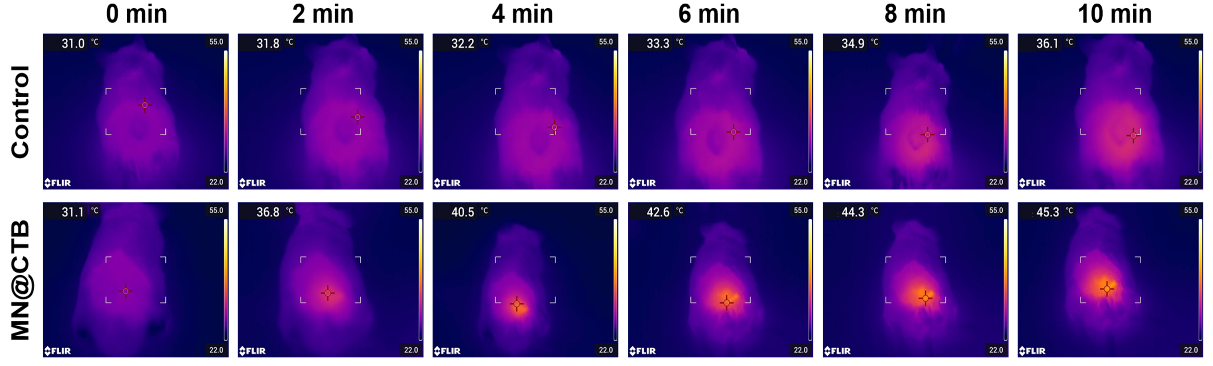
**

**Figure S30**. Representative real-time thermographic images under NIR irradiation for 10 min.





**Figure S31**. The corresponding photothermal heating curves (n = 3), ***p* < 0.01.


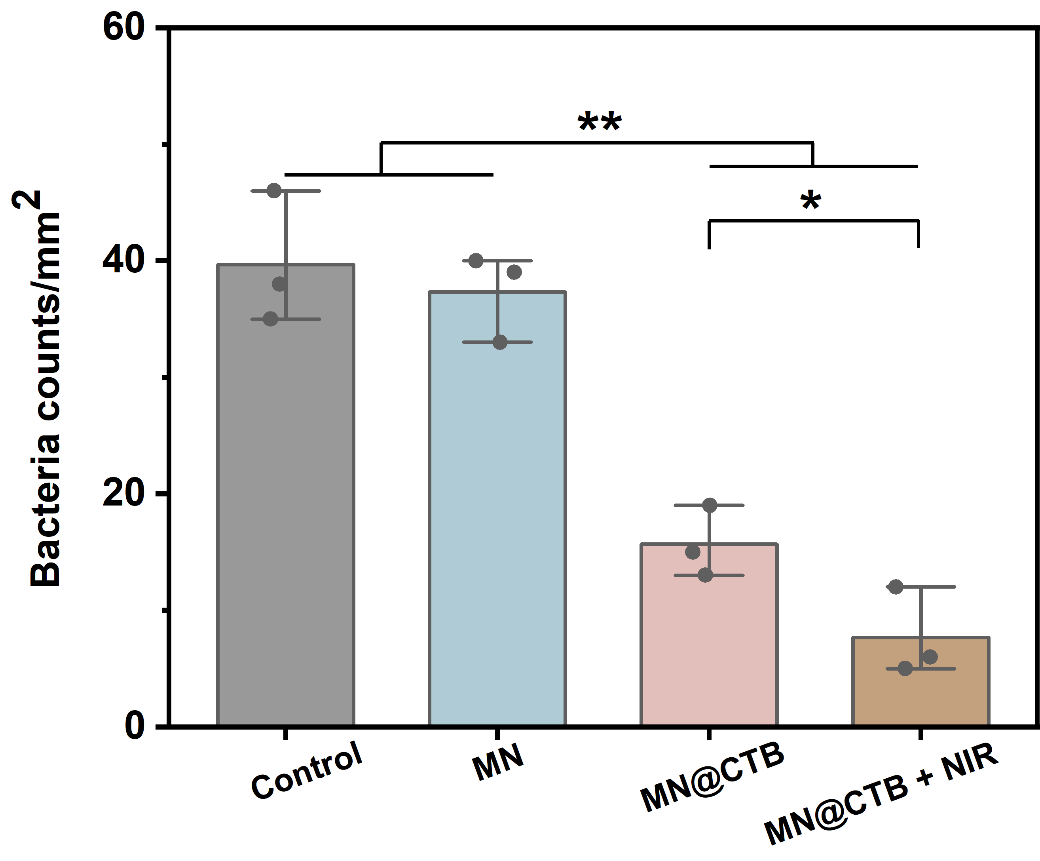


**Figure S32.** Statistical analysis of bacteria number in the wound tissues after different treatments in 3 days (n = 3), **p* < 0.05, ***p* < 0.01.


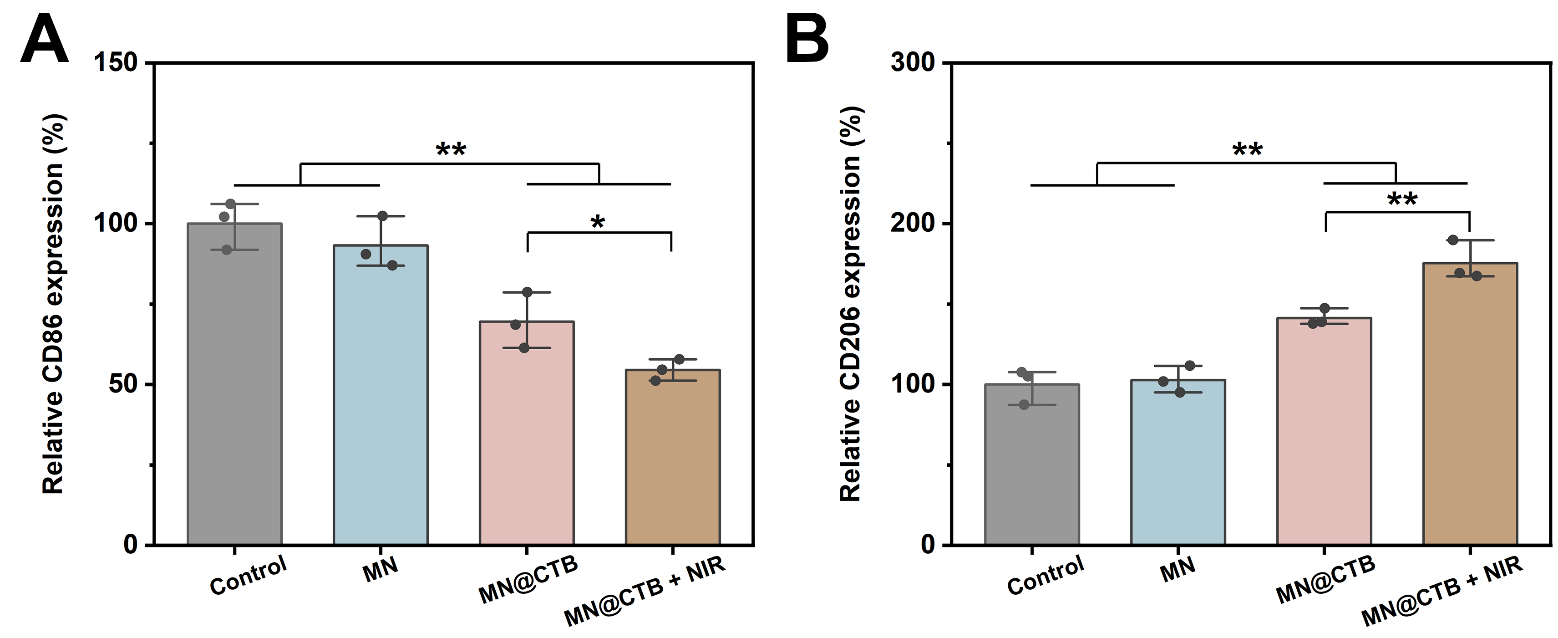


**Figure S33**. Quantitative analysis of CD86 and CD206 expression on day 7 in each group (n = 3), *p < 0.05, **p < 0.01.


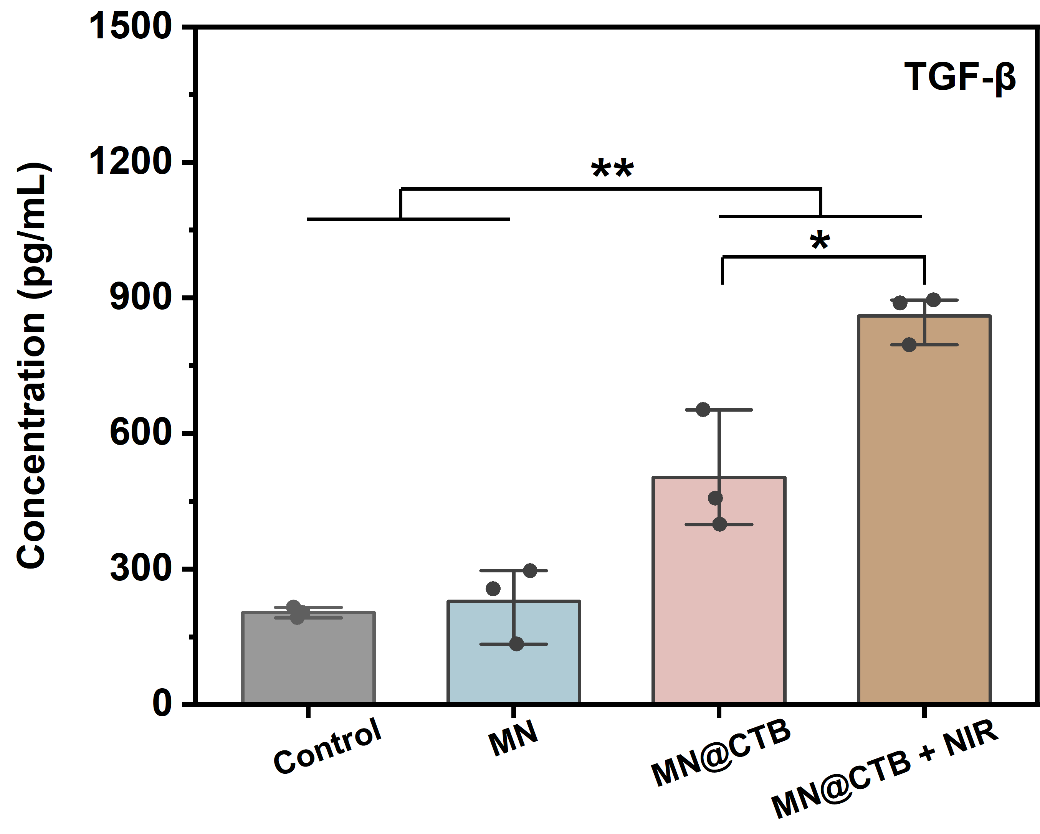


**Figure S34**. ELISA of TGF-β from the infected wounds on days 3 (n = 3), ***p* < 0.01.

**
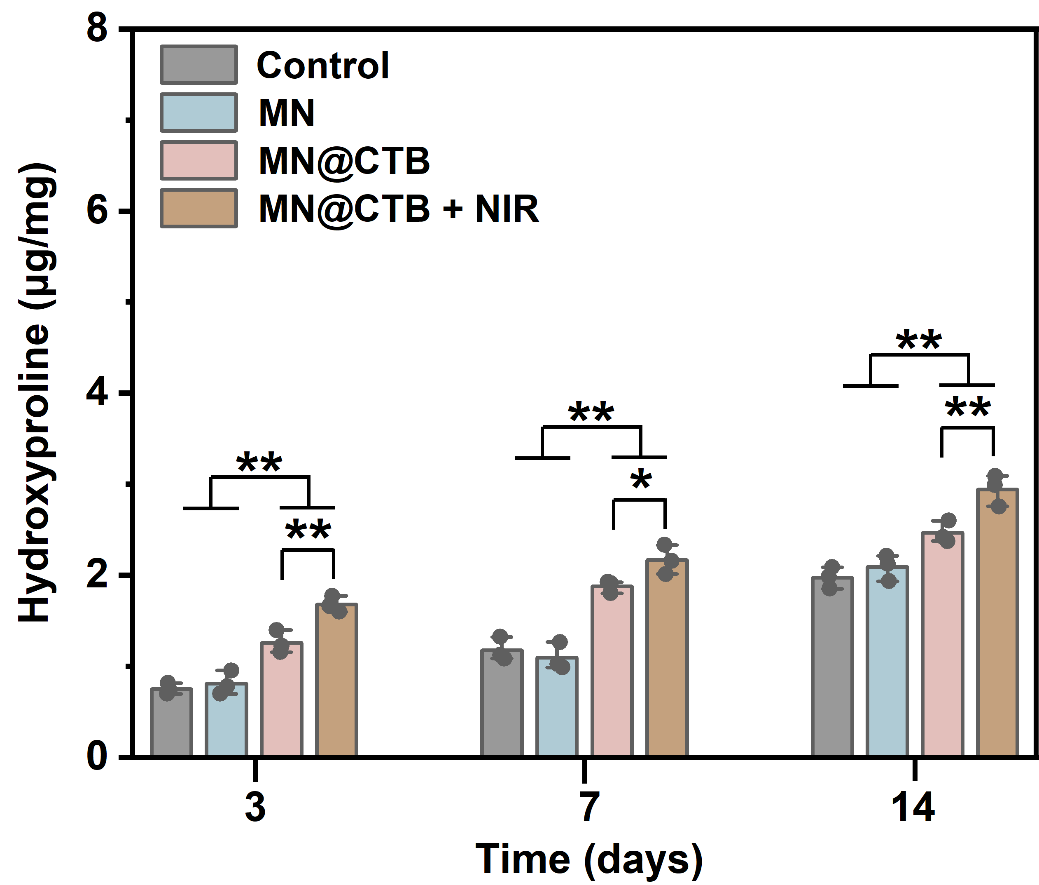
**

**Figure S35.** Quantification of hydroxyproline in the wound tissues after various treatments (n = 3), **p* < 0.05, ***p* < 0.01.

**
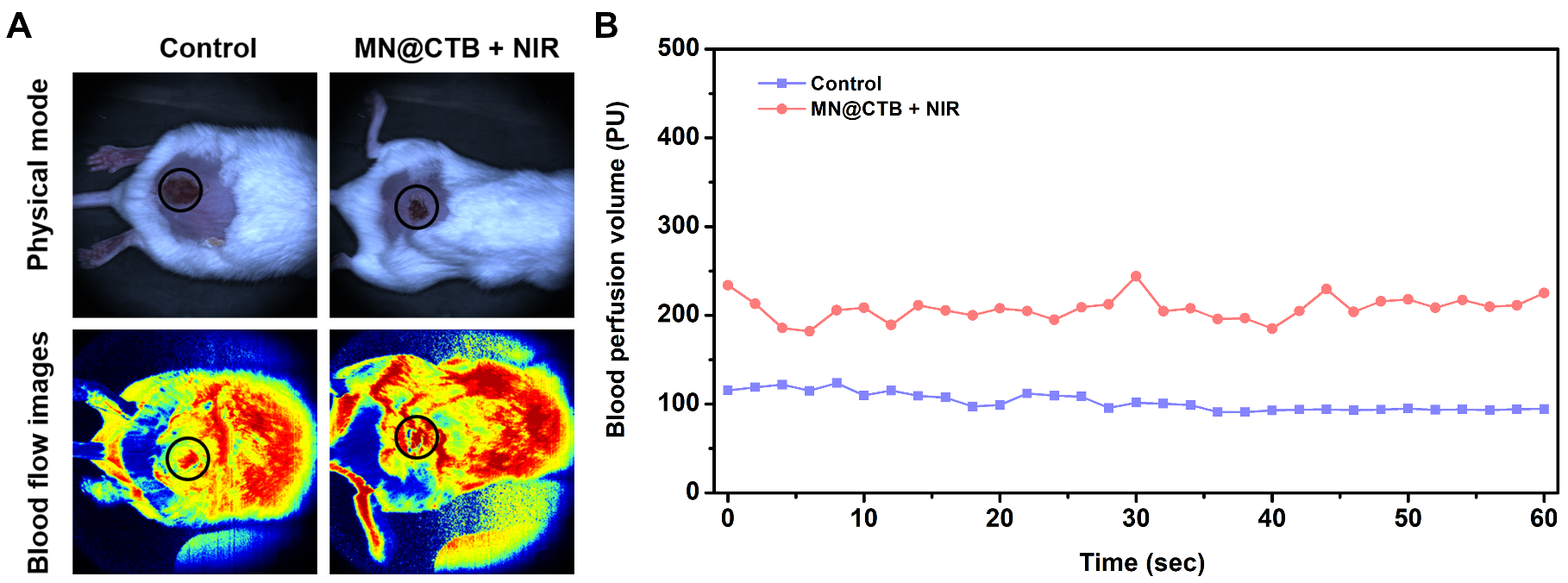
**

**Figure S36.** Evaluation of in vivo vascular integration**.** (A) Representative image of skin flap in physical mode and representative laser Doppler-scanned images of blood flow at wound sites. (B) The quantification of the blood perfusion volume in each group.


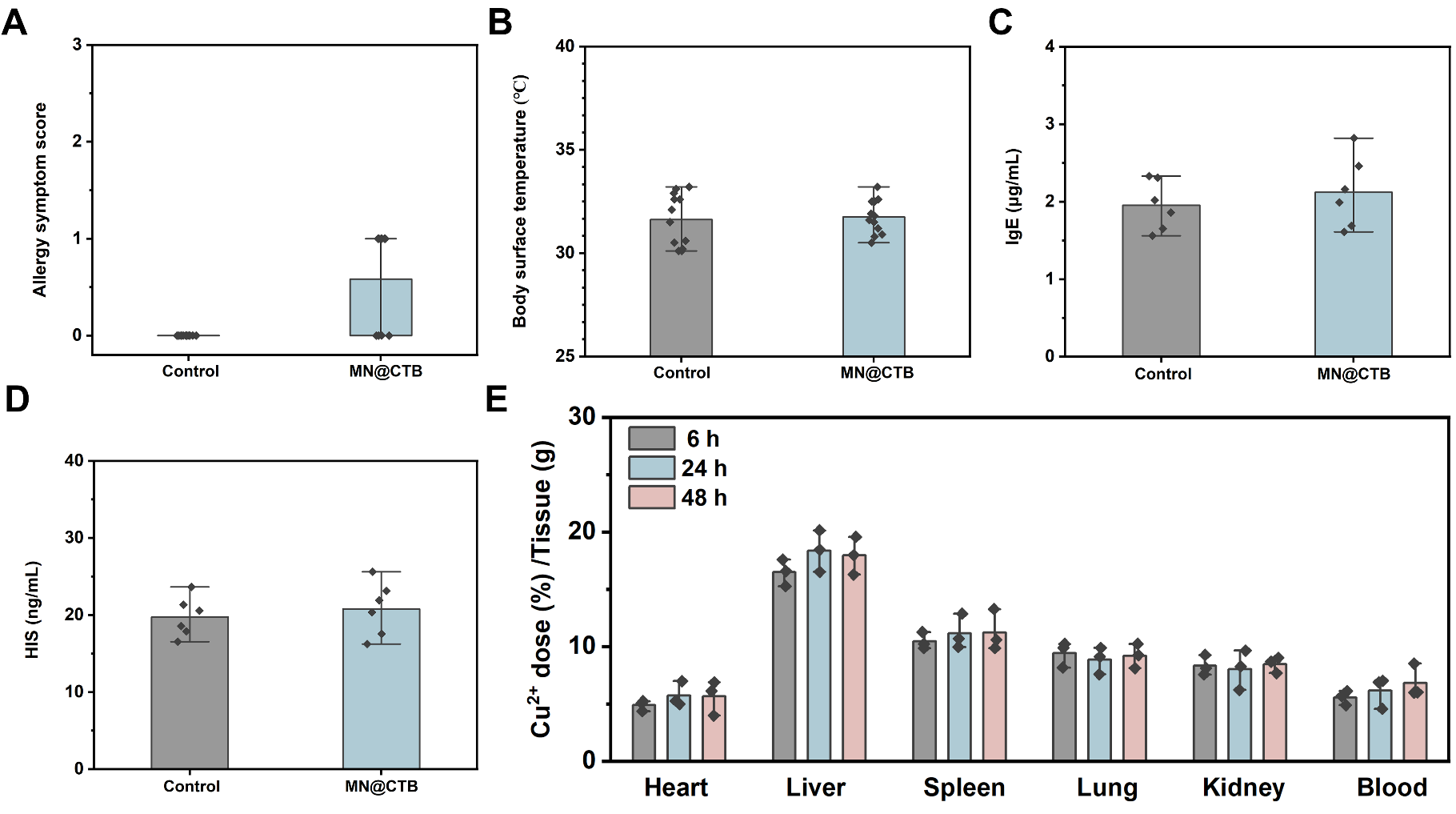


**Figure S37**. The effects of MN@CTB microneedle patches allergic reactions and metabolic processes *in vivo*. (A) Allergy symptom score (n = 12). (B) Body surface temperature (n = 12). (C) IgE (n = 6) and (D) HIS concentration in mice (n = 6). (E) The Cu distribution in the main organs (heart, liver, Spleen, lung, and kidney) and blood of MRSA-infected wounds mice after administration of MN@CTB microneedle patches for 6, 24, and 48 h by ICP assay (n = 3), **p* < 0.05, ***p* < 0.01.


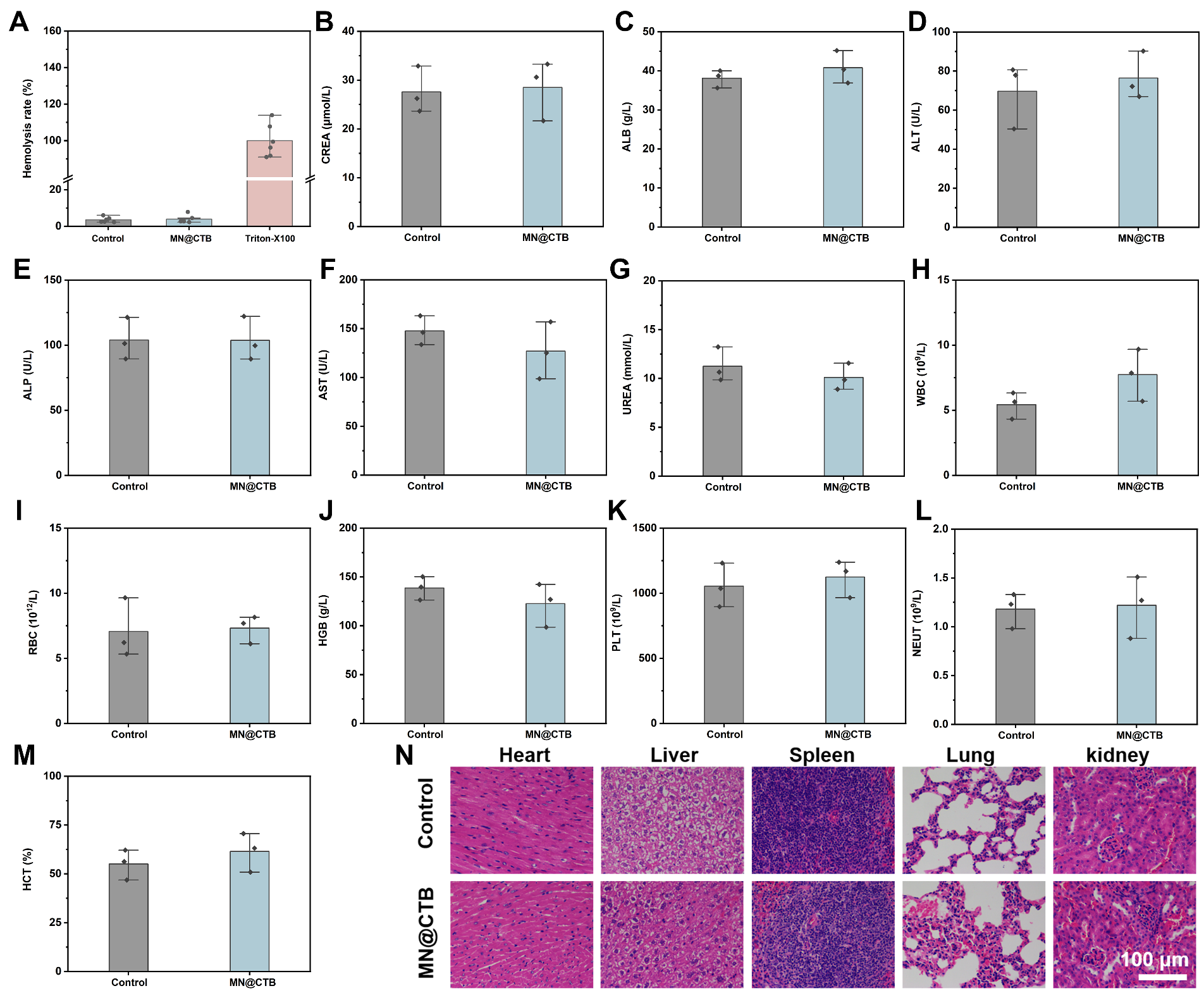


**Figure S38**. Evaluation of the biosafety and translational clinical application potential of MN@CTB microneedle patches *in vivo*. (A) The hemolysis rate (%) of MN@CTB microneedle patches (n = 6). (B-G) Serum biochemistry analysis after 14-day treatment (n = 3). Creatinine (CREA), Albumin (ALB), Alanine aminotransferase (ALT), Alkaline phosphatase (ALP), Aspartate aminotransferase (AST), and Urea (UREA). (H-M) Whole blood biochemical analysis of MN@CTB microneedle patches on day 14 (n = 3). White blood cells (WBC), Red blood cells (RBC), Hemoglobin (HGB), Platelets (PLT), Neutrophil granulocytes (NEUT), and Hematocrit (HCT). (N) H&E staining of the major organs after 14-day treatment, **p* < 0.05, ***p* < 0.01.
